# Deep learning links localized digital pathology phenotypes with transcriptional subtype and patient outcome in glioblastoma

**DOI:** 10.1093/gigascience/giae057

**Published:** 2024-08-26

**Authors:** Thomas Roetzer-Pejrimovsky, Karl-Heinz Nenning, Barbara Kiesel, Johanna Klughammer, Martin Rajchl, Bernhard Baumann, Georg Langs, Adelheid Woehrer

**Affiliations:** Division of Neuropathology and Neurochemistry, Department of Neurology, Medical University of Vienna, 1090 Vienna, Austria; Comprehensive Center for Clinical Neurosciences and Mental Health, Medical University of Vienna, 1090 Vienna, Austria; Center for Biomedical Imaging and Neuromodulation, Nathan Kline Institute, Orangeburg, NY 10962, USA; Computational Imaging Research Lab, Department of Biomedical Imaging and Image-guided Therapy, Medical University of Vienna, 1090 Vienna, Austria; Department of Neurosurgery, Medical University of Vienna, 1090 Vienna, Austria; Gene Center and Department of Biochemistry, Ludwig-Maximilians-Universität München, 80539 Munich, Germany; Department of Computing and Medicine, Imperial College London, London SW7 2AZ, UK; Center for Medical Physics and Biomedical Engineering, Medical University of Vienna, 1090 Vienna, Austria; Computational Imaging Research Lab, Department of Biomedical Imaging and Image-guided Therapy, Medical University of Vienna, 1090 Vienna, Austria; Division of Neuropathology and Neurochemistry, Department of Neurology, Medical University of Vienna, 1090 Vienna, Austria; Comprehensive Center for Clinical Neurosciences and Mental Health, Medical University of Vienna, 1090 Vienna, Austria; Department of Pathology, Neuropathology and Molecular Pathology, Medical University of Innsbruck, 6020 Innsbruck, Austria

**Keywords:** Glioblastoma, deep learning, histology, digital pathology, risk score

## Abstract

**Background:**

Deep learning has revolutionized medical image analysis in cancer pathology, where it had a substantial clinical impact by supporting the diagnosis and prognostic rating of cancer. Among the first available digital resources in the field of brain cancer is glioblastoma, the most common and fatal brain cancer. At the histologic level, glioblastoma is characterized by abundant phenotypic variability that is poorly linked with patient prognosis. At the transcriptional level, 3 molecular subtypes are distinguished with mesenchymal-subtype tumors being associated with increased immune cell infiltration and worse outcome.

**Results:**

We address genotype–phenotype correlations by applying an Xception convolutional neural network to a discovery set of 276 digital hematozylin and eosin (H&E) slides with molecular subtype annotation and an independent The Cancer Genome Atlas–based validation cohort of 178 cases. Using this approach, we achieve high accuracy in H&E-based mapping of molecular subtypes (area under the curve for classical, mesenchymal, and proneural = 0.84, 0.81, and 0.71, respectively; *P* < 0.001) and regions associated with worse outcome (univariable survival model *P* < 0.001, multivariable *P* = 0.01). The latter were characterized by higher tumor cell density (*P* < 0.001), phenotypic variability of tumor cells (*P* < 0.001), and decreased T-cell infiltration (*P* = 0.017).

**Conclusions:**

We modify a well-known convolutional neural network architecture for glioblastoma digital slides to accurately map the spatial distribution of transcriptional subtypes and regions predictive of worse outcome, thereby showcasing the relevance of artificial intelligence–enabled image mining in brain cancer.

## Background

Computer vision has undergone a revolution in recent years, which was in large parts driven by the development of convolutional neural networks (CNNs) [1–3]. In digital pathology, major achievements included the precise segmentation of individual cells [4–7], histologic structures [8, 9], and tumor tissues [10]. In glioma, so far, CNNs have been employed for tumor typing, grading, and prognostic rating [11–13]. Still, the links between histologic phenotypes and underlying genotypes remain insufficiently understood, a gap that could be addressed using CNNs [2, 3].

Glioblastoma is the most common and fatal brain tumor in adults [14]. Prognostic factors include patient age, clinical performance, tumor location and resectability, DNA methylation at the MGMT gene promoter, and receipt of multimodal treatment [15–17]. Furthermore, multiple studies have highlighted the potential for histology-based prognostic biomarkers for gliomas in general and glioblastoma in particular [11, 18–20].

At the histologic level, glioblastoma is characterized by extensive within- and across-tumor variability ranging from small-celled to monstro-cellular and sarcomatous cells with recurrent formation of palisades around necroses and Scherer’s secondary structures at the invasive front. Also, the composition of the microenvironment varies in space and time with bone marrow–derived macrophages being abundant in necrotic regions, brain-resident microglia within and surrounding tumor regions, and scattered lymphocytes in perivascular arrangements.

At the level of tumor biology, glioblastoma is characterized by complex genetic aberrations and transcriptional plasticity with considerable spatial and temporal variability (Supplementary Fig. S1) [21–24]. At the bulk level, 3 transcriptional subtypes were defined (i.e., classical, mesenchymal, and proneural), each being enriched for genetic alterations and microenvironmental factors [25]. Importantly, previous efforts to explore the spatial distribution of the transcriptional subtypes pointed toward associations between the proneural subtype and invasive edges with enhanced neuronal signaling, as well as the mesenchymal subtype and perinecrotic areas with denser immune cell infiltration [26–28]. However, despite their biologic relevance, their translation into routine clinical assessments based on formalin-fixed, paraffin-embedded (FFPE) tissues was largely prevented by the limited availability of FFPE-based spatial transcriptomics technology. Hence, a computational solution that enables their accurate prediction in spatial context based on ubiquitously available, cost-efficient hematoxylin and eosin (H&E) stains would fuel their translation and clinical applicability.

**Figure 1:**
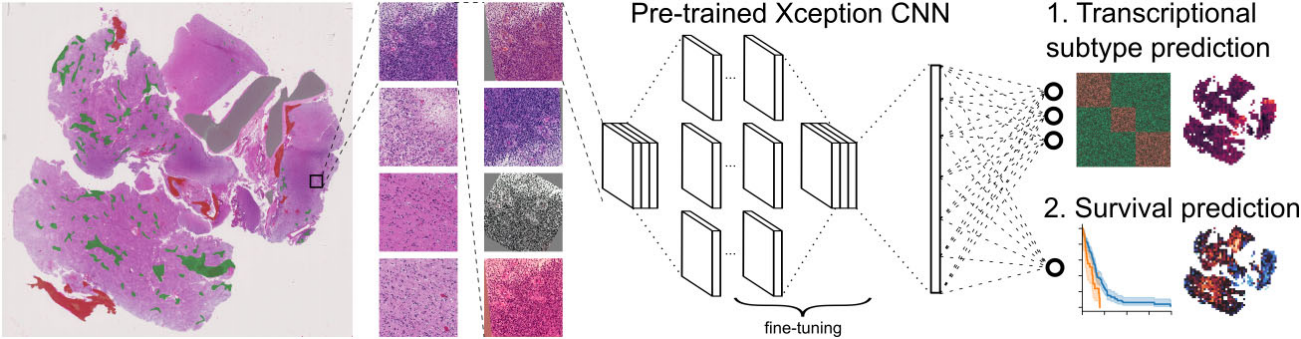
Approach and network architecture. Digital slides were manually segmented (green: necrosis, red: bleeding, gray: preexisting tissue) and smaller image tiles comprising mainly/only tumor tissue and infiltration zone were exported. For model training, extensive data augmentation was performed. The deep learning framework consisted of a pretrained Xception CNN, in which the ultimate layer was replaced by a 3-neuron layer (for transcriptional subtype prediction, TS-CNN) or a 1-neuron layer (for survival prediction, RS-CNN). We used only histological image tiles as input to the model, and no other information was introduced to either model.

Here, we introduce an end-to-end CNN based on a modified Xception architecture that generates a histology-based risk score to estimate patient prognosis (RS-CNN) and maps the spatial distribution of transcriptional subtypes (TS-CNN, Fig. 1).

## Materials and methods

### Patient cohort

We leveraged an existing longitudinal IDH–wild-type glioblastoma patient cohort comprising matched histological and DNA methylation-derived transcriptional subtypes at the time of first and second surgery [22].

A total of 276 patients with digital histology and outcome data were included (Table 1, *discovery cohort*) to train the RS-CNN using overall survival as a label. For 189 tumors, also transcriptional subtype information was available (Table 1, *TS subcohort*), including the admixture of the different subtypes (summing up to 100%), which was used as ground truth for training [22]. Samples with at least 70% contribution by a given subtype were allocated to this subtype (e.g., classical-predominant, proneural-predominant, mesenchymal-predominant). Both the entire discovery cohort and the TS subcohort featured a similar age range and female-to-male ratio. However, the TS cohort was slightly biased toward an increased receipt of temozolomide-based radiochemotherapy and prolonged survival. We ultimately split each cohort into 5 equally large folds with comparable characteristics for internal 5-fold cross-validation.

**Table 1:** Demographics of the discovery cohort and the TS subcohort. The whole discovery cohort was used for risk score prediction. The TS subcohort was used for TS prediction. CNN: convolutional neural network; IQR: interquartile range; TMZ: temozolomide; TS: transcriptional subtype.

		Discovery cohort	TS subcohort
**Number of patients**		276	189
**Median age [IQR]**		63.0 [53.8–70.5] years	62.0 [52.0–68.0] years
**Female/male ratio**		0.62 (106:170)	0.64 (74:115)
**Combined radiochemotherapy (TMZ)**		205 (74.3%)	153 (81.0%)
**Median overall survival**		1.16 years	1.51 years
**Alive at last follow-up**		7 (2.54%)	7 (3.7%)
**TS**	**Classical predominant**	—	34 (17.99%)
	**Mesenchymal predominant**	—	50 (26.46%)
	**Proneural predominant**	—	21 (11.11%)
	**Mixed**	—	84 (44.44%)

### Handling of digital slides

H&E sections were digitized using a Hamamatsu NanoZoomer 2.0 HT slide scanner. On each digital slide, necrosis, preexisting brain parenchyma, bleeding, scar tissue, and deformed tissue had been manually segmented by a board-certified neuropathologist (A.W.) using the ndp.view2 built-in annotation tool. The remaining areas were assigned to tumor areas. Each digital slide was converted to multiple (i.e., 6 to 2,257) 1,024 × 1,024–pixel tiles at 20× magnification (456 pixels/μm) with a 64-pixel overlap using a custom MATLAB script (MATLAB R2017b; MathWorks) [29, 30]. An accompanying spreadsheet contained the coordinates of each tile with the relative areas per segmented region. We defined perinecrotic regions as image tiles containing both tumor tissue and necrosis. Similarly, we defined the infiltration zone as tiles containing both tumor and preexisting tissue. For classifier training, only tiles with >50% tumor tissue were kept. Patients with fewer than 50 different tiles had been excluded from further analysis. For training, we performed random cropping to 512 × 512 pixels and automated data augmentation with the H&E-specific algorithm of Faryna at runtime [31].

### CNN architecture

We used TensorFlow 2.1.0/keras for developing our deep learning pipeline [32]. As a base model, we used an Xception architecture [33] pretrained on ImageNet available via the keras model applications [34]. This architecture was chosen because of its high efficiency with similar or increased performance compared to other more commonly used architectures like ResNet [33, 35]. We refrained from using models that were pretrained with histological data instead of the more generic ImageNet data as they incorporate data from The Cancer Genome Atlas (TCGA) for the training. Since we here use TCGA data for independent external validation, using the same data for pretraining and validation would result in mixing of training and test data.

The input to the Xception network consisted of a (randomly sampled) WSI tile, and no other information was introduced to the model. We froze all weights and added an extra layer depending on the target. For TS prediction, we added a fully connected 3-neuron layer with softmax activation. The TS target consisted of the 3 probabilities for each of the transcriptional subtypes. The mean squared error was backpropagated to update the weights. For risk score (RS) prediction, we added a single 1-neuron Cox regression layer with a linear activation function. The negative log-likelihood was used as a loss function and backpropagated to update the weights in a similar approach as Mobadersany et al. [11]. We adapted keras’ DataFrameIterator such that for each new cycle through the digital slides, a new random image tile was selected per patient, randomly cropped, and augmented. The TS-CNN and RS-CNN were trained independently of each other. Each model was first trained for 25 epochs with a custom 150 steps per epoch (for better performance) and a batch size of 64. We used the Adam optimizer with a learning rate of 0.001 and exponential learning rate decay every 400 steps at a decay rate of 0.9. For fine-tuning, the last 2 convolutional layers (4,741,632 of 20,861,480 parameters) of the Xception model were set trainable, and the model was trained for 10 further epochs with 150 steps per epoch and a batch size of 64. Again, we used the Adam optimizer with a learning rate of 0.0001 and exponential learning rate decay every 400 steps at a decay rate of 0.8. During training, at the start of each fold, 20 random batches were loaded into memory for validation. At the end of each epoch, the mean squared error (for TS prediction) or the c-index (for survival prediction) was calculated for the validation batches to keep track of the model performance.

We used 5-fold cross-validation during model training. For the final validation, we let the trained models predict all validation tiles (with center crop to 512 × 512 pixels and no augmentation). The RS predictions were *z*-scored, the TS predictions were taken as they were, and then all validation set predictions were concatenated into a single spreadsheet for further statistical analysis.

### H&E mapping

To visualize the spatial distribution of the predicted targets directly in the digital slides, we performed the predictions on a set of windows covering the entire digital slide. We then mapped the predictions to the coordinates of those windows. Thereby, heatmaps were plotted in triplets representing the 3 transcriptional subtypes or as a single map depicting the risk score [36].

### Statistical analysis

Statistical analysis was conducted in Python 3.8.5. We performed permutation tests by label shuffling to compare our predicted risk scores to random guesses. To calculate *P*-values determining the significance of the RS and TS predictions, we performed label shuffling to generate a null distribution. Mann–Whitney *U* and Wilcoxon tests were calculated with scipy [37]. Kaplan–Meier survival analysis and Cox proportional hazards models were performed using lifelines [38]. Harrel’s c-index was calculated using sksurv [39]. Figures were drawn using matplotlib [40] and seaborn [36]. The confusion matrix and roc analysis were performed using sklearn [41]. To compare RS with TS scores, we assigned each tile to the subtype displaying the highest predicted score (winner-takes-all). Based on that annotation, we then calculated the mean risk score for each transcriptional subtype.

For Uniform Manifold Approximation and Projection (UMAP) plotting, we first concatenated the outputs of the penultimate CNN layers of all models obtaining 20,480 features for each image tile. We then used the umap package to plot UMAPs.

### Characterization of the tumor microenvironment

We used QuPath 0.3.0 [42] for the following steps. To showcase the within-tumor histological variability, we used the inbuilt “density map” function. We first performed “fast cell counts” on the H&E digital slides to obtain overall cellularity (i.e., cell density) and circularity (i.e., cell *roundness*). The tumor cell proliferation, tumor-associated macrophage (TAM), and tumor-infiltrating lymphocyte (TIL) density maps were calculated from Ki-67-, CD68-, CD163-, HLA-DR-, and CD8-stained digital slides using “positive cell detection.” The immunohistochemical stainings were performed on a Dako autostainer system with the following antibodies: CD3 (Thermo Scientific no. RM-9107-S1, 1:200), CD8 (Dako Cytomation no. M7103, 1:100), CD163 (Novocastra no. NCL-L-CD163, 1:1,000), CD68 (Dako Cytomation no. M0814, 1:5,000), HLA-DR (Dako Cytomation no. M0775, 1:400), Ki-67 (MIB-1) (Dako Cytomation no. M7240, 1:200), and CD34 (Novocastra no. NCL-l-END, 1:100) [22]. To link TAM and TIL densities with transcriptional subtypes and risk tiles, we manually segmented the respective regions on neighboring digital slides (where available and adequate) (Supplementary Table S1). After using “positive cell detection,” we counted all stained cells in each region and divided this count by the respective area to obtain the number of stained cells per mm². For HLA-DR and CD34, we calculated the relative stained area in a similar fashion. Thus, we obtained a quantitative characterization of the tumor microenvironment per slide/patient. We calculated summary statistics on this slide/patient level to compare the different transcriptional subtype regions and high/low-risk regions. The QuPath script with the specific parameters is provided via github [43].

### External validation using TCGA data

After successful internal validation, we retrained our CNN models on our complete discovery dataset using the same parameters as previously stated. We then downloaded the clinical annotation for the TCGA glioblastoma cohort published by Brennan et al. [44] from cBioPortal [45]. We screened the GDC Data Portal for available diagnostic slides and downloaded them using the GDC Data Transfer Tool [46]. To match the inclusion criteria of our training cohort, we excluded slides of suboptimal quality (due to excessive artifacts, poor staining, or non-FFPE H&E slides) and tumors with mutant or unknown IDH status. We manually segmented the tumor tissue and infiltration zone in concordance to the discovery cohort. We then applied the RS- and TS-CNNs to the validation set. We averaged the subtype predictions over all image tiles and let the highest subtype score determine the predicted subtype per sample. We considered samples with a mismatch between predicted subtype and TCGA bulk sequencing-derived subtype as misclassified. Moreover, patients were assigned to 2 risk groups, depending on the fraction of *high risk* (*z*-score >1) tiles (cutoff 25%). High-risk samples of patients who survived >18 months and low-risk samples of patients with <12-month survival were considered misclassified.

## Results

### H&E-based mapping of transcriptional subtypes

**Figure 2:**
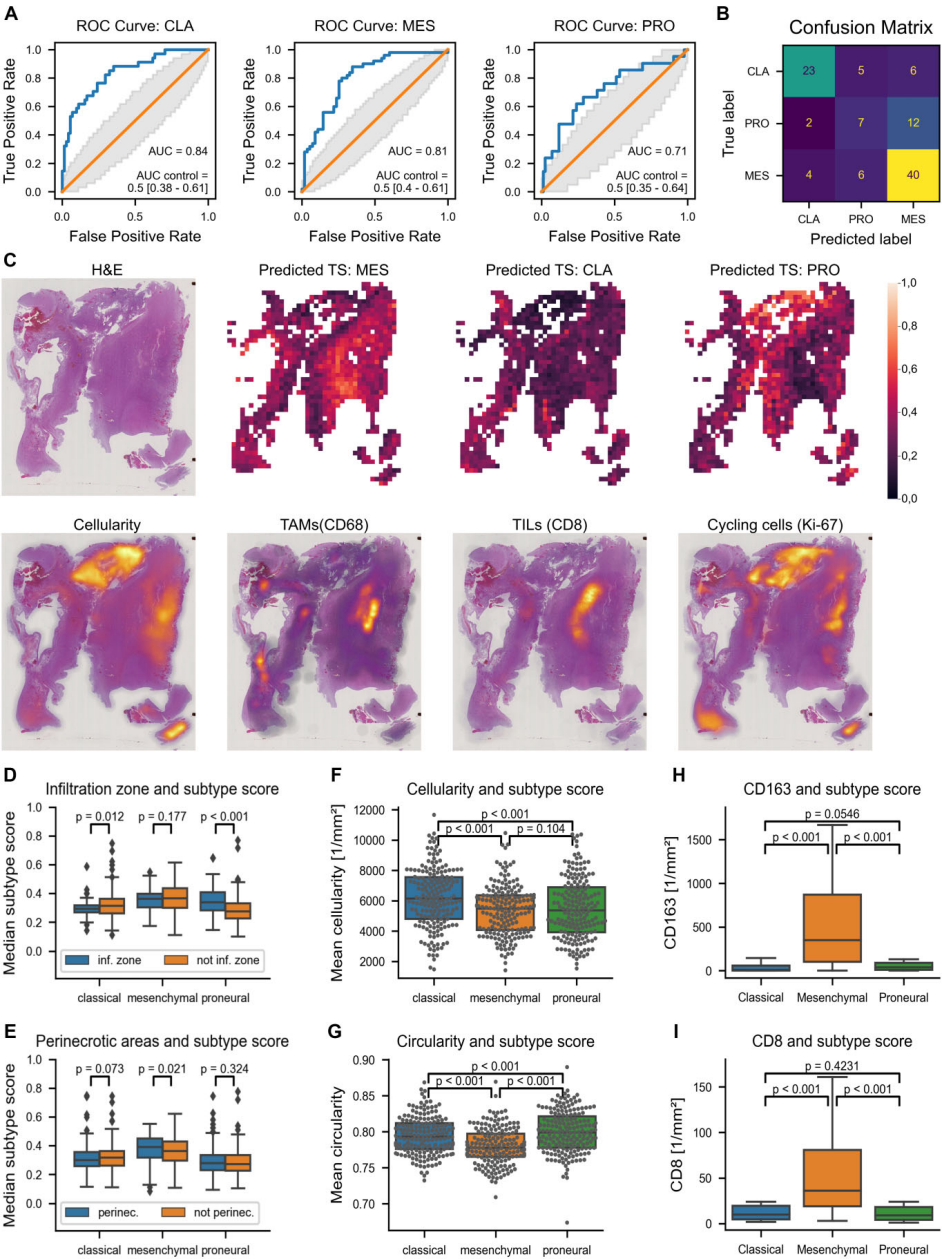
Prediction of the transcriptional subtypes (TS). (A) Receiver operating characteristic (ROC) curves and area under the curve per TS (B) confusion matrix for the prediction of TS in samples with a predominant TS. (C) One representative sample with 3 heatmaps depicting TS scores at the whole slide level and density maps for cell density, CD68^+^ TAMs, CD8^+^ TILs, and Ki67^+^ cycling cells. (D–I) Correlative analyses between different histological aspects and TS scores. Of note, single outliers in panels (I) and (J) are not shown for better illustration.

The accuracy for predicting the predominant subtype was 66.7% as compared to a random guess accuracy of 38.67% ± 0.4% (*P* < 0.001, permutation test, Fig. 2A, B). The mean squared error was 0.08 in the validation folds as compared to 0.11 ± 0.003 for random predictions (*P* < 0.001, permutation test). Overall, the spatial distribution of subtypes aligned well with the segmented tumor regions (Fig. 2C, D) both upon visual inspection of the heatmaps as well as upon quantification at the cohort level. Precisely, median predictive scores were significantly higher for proneural in the infiltration zone (*P* < 0.001, Mann-Whitney U (MWU), Fig. 2E) and for mesenchymal in perinecrotic areas (*P* = 0.021, MWU, Fig. 2F). Likewise (Table 2), a significantly higher cellularity and a tendency to larger fractions of cycling cells were found in classical areas (*P* < 0.001, Wilcoxon test, Fig. 2G, *P* < 0.05, MWU). At the individual cell level, nuclear circularity was highest in proneural and lowest in mesenchymal areas (all *P* < 0.001, Wilcoxon, Fig. 2H). Ultimately, we found increased infiltration by CD68^+^, CD163^+^, and HLA-DR^+^ myeloid cells and CD3^+^, CD8^+^ TILs in mesenchymal regions (all *P* < 0.006, MWU, Fig. 2I). Likewise, areas covered by CD34^+^ vessels were enriched in mesenchymal as compared to proneural (*P* < 0.01, MWU) or classical (*P* = 0.02, MWU) regions.

**Table 2:** Comparison of cellular phenotype and immunohistochemical parameters [median, IQR] between different predicted TS. Given values represent a summary statistic over all slides, and the whole respective subtype region (if present on their digital slide) was evaluated for each patient. The *P*-values were calculated using the Kruskal–Wallis H-test.

	Classical	Mesenchymal	Proneural	*P*-value
**Cellularity (per mm²)**	6146 [4800–7574]	5484 [4046–6353]	5321 [3897–6833]	<0.001
**Circularity**	0.79 [0.78–0.81]	0.78 [0.77–0.8]	0.8 [0.78–0.82]	<0.001
**CD163⁺ cells (per mm²)**	9 [1–59]	348 [101–871]	37 [8–91]	0.027
**CD3⁺ cells (per mm²)**	34 [17–82]	129 [53–310]	25 [15–52]	0.006
**CD68⁺ cells (per mm²)**	96 [23–277]	243 [98–573]	72 [24–194]	<0.001
**CD8⁺ cells (per mm²)**	10 [5–20]	36 [19–81]	9 [4–18]	<0.001
**MIB⁺ cells (per mm²)**	290 [128–630]	108 [59–214]	60 [26–581]	<0.001
**CD34**	4% [3–6]	5% [4–11]	2% [1–4]	<0.001
**HLA-DR**	2% [0–9]	8% [4–18]	1% [0–3]	<0.001

### H&E-based risk score prediction

**Figure 3:**
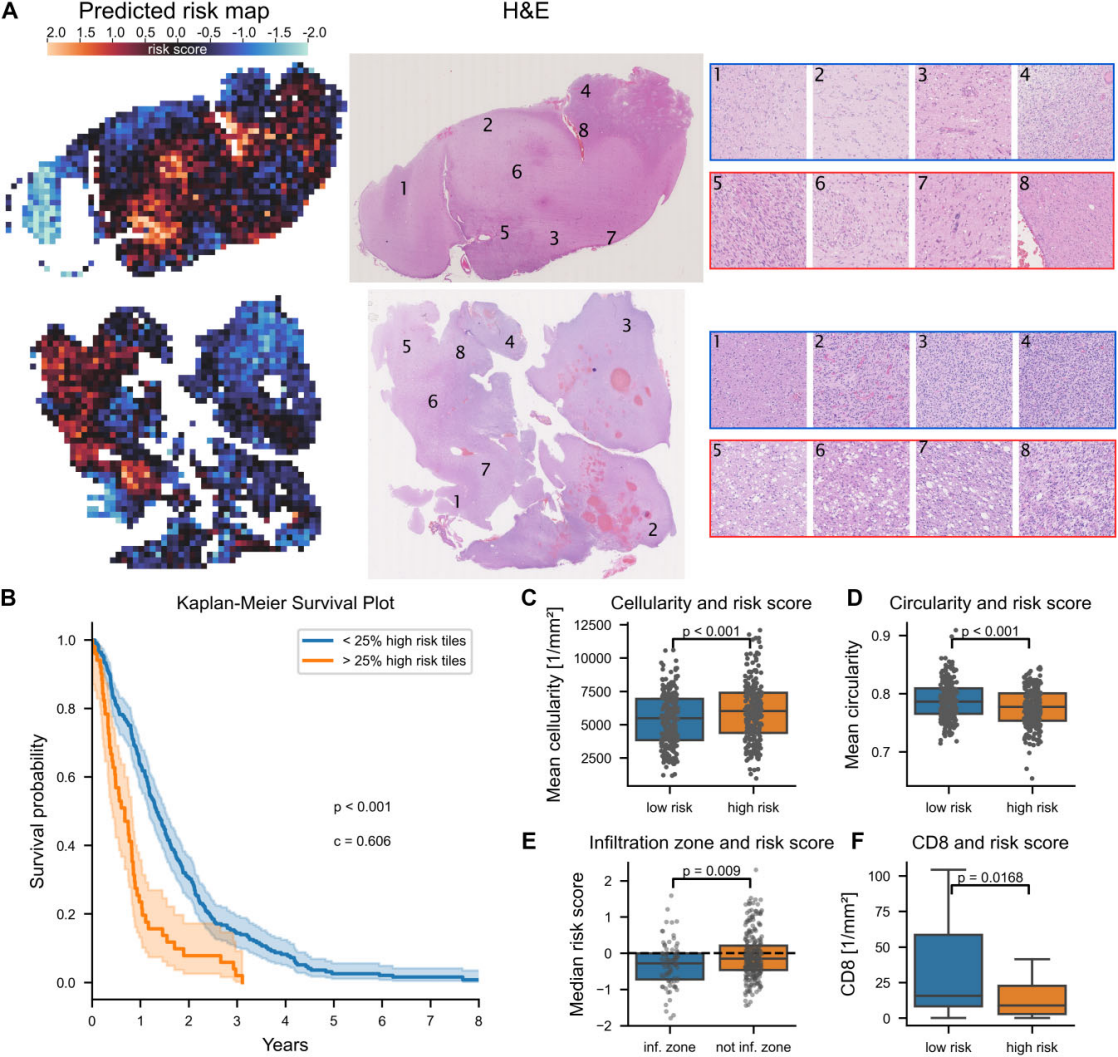
Prediction of the risk score. (A) Risk maps and corresponding H&E slides of 2 representative cases. Higher risk scores are depicted in a red hue and lower risk scores in a blue hue. Higher-magnification images are given for the numbered regions of the WSI (top row: low-risk regions, bottom row: high-risk regions). (B) Kaplan–Meier plot stratified at 25% high-risk tiles (*P* < 0.001, log-rank test, Harrel’s c-index = 0.6). (C–F) Boxplots depicting associations between risk scores and selected histological and immunohistochemical aspects.

The risk score prediction model (RS-CNN) was trained end-to-end on histological images alone using the Cox loss function (negative log-likelihood), which yielded a single risk score as output. To obtain patient-level predictions, the predicted scores per tile were normalized (*z*-scored) across the entire cohort and aggregated using the arithmetic mean. Additionally, the fraction of high-risk tiles (*z*-scored risk >1) was calculated per digital slide and their distribution plotted as a heatmap (Fig. 3A). In the validation folds, the risk scores were strongly associated with survival upon univariable (*P* < 0.001, Fig. 3B) and multivariable analyses (*P* = 0.013, Table 3). Of note, MGMT promoter methylation status was not included in the multivariable model as it was only available for a subset of 41 patients, which would have limited the statistical power for the detection of an association of survival and risk score (power of 0.20 [47]). In this smaller subset, only age and radiochemotherapy remained as statistically significant prognostic factors, while MGMT status and the risk scores failed to reach statistical significance (Supplementary Table S2).

**Table 3:** Cox multivariable survival model. HR for age is calculated for each 1-year increase of patient age.

	HR	*P*-value
**Age**	1.025 (1.015–1.036)	<0.001
**Male sex**	1.14 (0.88–1.48)	0.331
**Radiochemotherapy (TMZ)**	0.43 (0.32–0.58)	<0.001
**RS-CNN**	1.32 (1.06–1.65)	0.013

The median risk score was significantly lower in infiltration zones (Fig. 3E & Table 4, *P* = 0.009, MWU) and not enhanced in perinecrotic areas (*P* = 0.446, MWU). High-risk areas were characterized by higher cellularity (*P* < 0.001, Wilcoxon), decreased nuclear circularity (reflecting polymorphous nuclei, *P* < 0.001, Wilcoxon), fewer CD8^+^ cells (*P* = 0.017, MWU), and a trend toward fewer CD3^+^ cells (*P* = 0.06, MWU). There was no significant difference in CD68^+^, CD163^+^, or HLA-DR^+^ myeloid cell density (*P* = 0.13, 0.435, and 0.25, respectively, MWU), the fraction of cycling cells (*P* = 0.19, MWU), and microvessel density (*P* = 0.31, MWU).

**Table 4:** Comparative analysis between high- and low-risk regions across histological and immunohistochemical parameters [median, IQR]. *P*-values were calculated using the Wilcoxon signed-rank test (Cellularity, Circularity) and the Mann–Whitney *U* test (immunohistochemical stainings), respectively.

	High risk	Low risk	*P*-value
**Cellularity (per mm²)**	5,877 [4,336–7,302]	5,524 [3,885–6,891]	<0.001
**Circularity**	0.78 [0.75–0.8]	0.79 [0.77–0.81]	<0.001
**CD163⁺ cells (per mm²)**	46 [8–234]	27 [7–366]	0.35
**CD3⁺ cells (per mm²)**	33 [16–70]	38 [22–221]	0.063
**CD68⁺ cells (per mm²)**	108 [26–219]	157 [45–274]	0.127
**CD8⁺ cells (per mm²)**	9 [3–23]	16 [8–58]	0.017
**MIB⁺ cells (per mm²)**	248 [45–634]	138 [48–340]	0.191
**CD34**	3 [2–5] %	4 [2–6] %	0.306
**HLA-DR**	3 [0–9] %	1 [0–9] %	0.25

### Integration of risk scores with transcriptional subtypes

**Figure 4:**
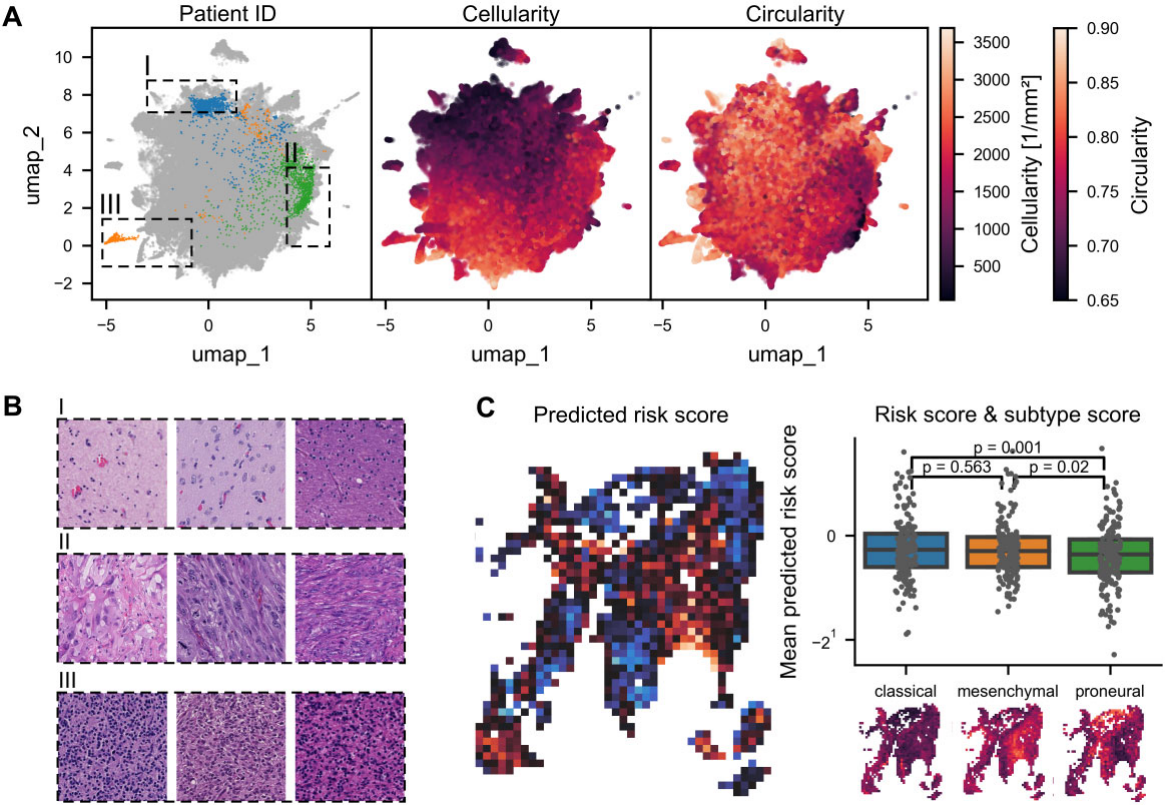
Feature landscape and integration of risk and transcriptional subtype scores. (A) UMAP projection of all features with highlighted regions I to III. In the leftmost UMAP, image tiles from 3 exemplary patients are highlighted in blue, green, and orange, respectively, and all other patients are plotted in gray. (B) Representative histological images corresponding to regions I to III with (I) corresponding to infiltration zone, (II) monstro-sarcomatoid phenotypes, and (III) round-cell and cell-dense regions. (C) Correlation between risk and transcriptional subtype prediction.

Ultimately, we aimed to link predicted risk scores with TS scores. Dimensionality reduction of aggregated TS and RS features resulted in 1 continuous feature space with smaller peripheral clusters that mostly represented individual patients. Still, also regional clusters relating to gross histologic features such as cellularity or nuclear circularity emerged (Fig. 4A, B).

Furthermore, we calculated the mean predicted risk score for each of the transcriptional subtypes per slide, which resulted in significantly higher risk scores in classical and mesenchymal than in proneural areas (Fig 4C, *P* = 0.001 and 0.02, respectively, Wilcoxon).

### External validation in TCGA datasets

**Figure 5:**
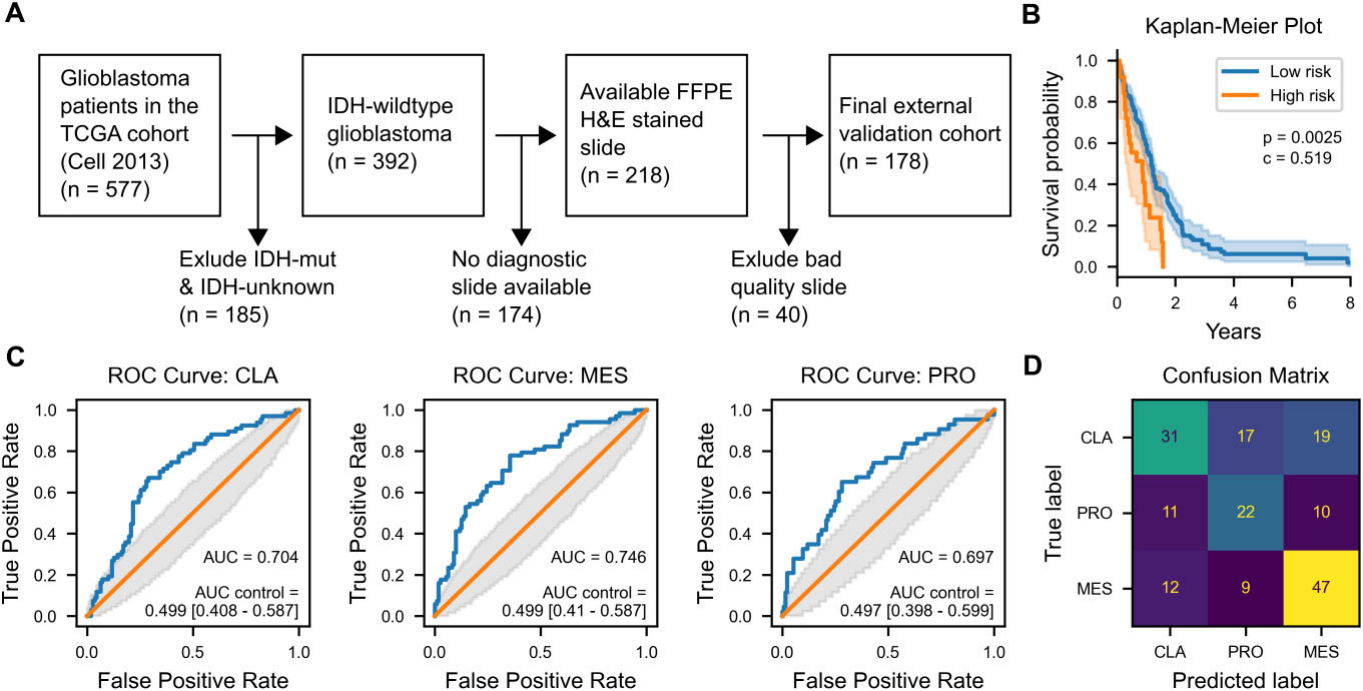
External validation in TCGA data [44]. (A) Inclusion flowchart to match inclusion criteria for the training cohort. (B) Kaplan–Meier plot stratified at content of 25% high-risk tiles (*P* = 0.003, log-rank test). (C) ROC and AUC values per TS. (D) Confusion matrix with TS prediction accuracy.

Finally, we sought to validate the performance of our models in an independent TCGA dataset (Fig. 5A). Applying the previously defined cutoff of 25% high-risk tiles resulted in a statistically significant separation of survival curves (*P* = 0.003, log-rank test, Fig. 5B). Of note, 14% of the validation set was assigned to the high-risk group, as compared to 18% in the discovery cohort. Harrel’s c-index was 0.52, and the mean risk score was not significantly associated with survival (Cox regression univariable hazard ratio [HR] = 1.4 ± 0.25, *P* = 0.16; multivariable HR = 1.2 ± 0.18, *P* = 0.31). In parallel, the accuracy for predicting the transcriptional subtypes was 56.2% compared to a random guess accuracy of 34.3% ± 0.4% in the validation set (*P* < 0.001, permutation test, Fig. 5C, D). Interestingly, the accuracy was highest for predicting the mesenchymal subtype (area under the curve [AUC] = 0.746) as compared to the classical (AUC = 0.704) and proneural (AUC = 0.697) subtypes.

**Figure 6:**
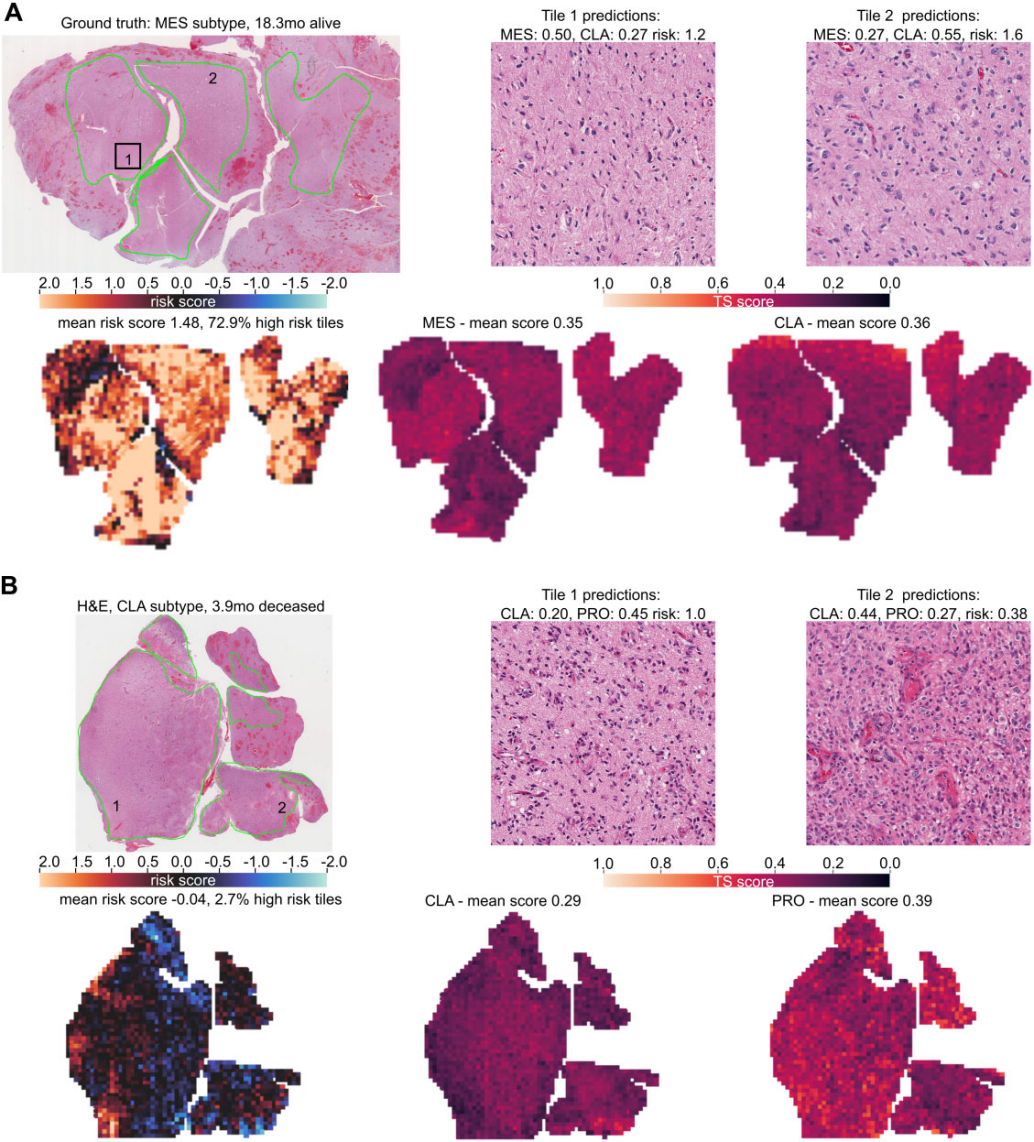
Misclassified TCGA samples. (A) Patient with >18 months of survival and mesenchymal subtype who was misclassified as high risk and classical subtype. (B) Patient with 3.9 months of survival and classical subtype who was misclassified as proneural subtype. Green segmentations on the H&E correspond to the manually segmented tumor tissue, upon which the CNN prediction was based.

To better understand the potential drawbacks and pitfalls of the trained CNNs, we specifically looked at misclassified samples (Fig. 6). Overall, out of 178 total samples, 78 displayed misclassified transcriptional subtypes, 3 were misclassified as high risk, and 57 were misclassified and low risk. For 28 samples, both the transcriptional subtype and survival were misclassified. We found that many (29.5%) subtype misclassifications were “near correct” (i.e., the difference between the true subtype score and the predicted subtype score was <0.01). Upon qualitative assessment of the misclassified cases, we further found that many samples had relatively little tumor tissue.

## Discussion

In the present study, we leverage deep learning on digital glioblastoma slides to address 2 relevant applications: (i) the mining of subvisual histological patterns for prognostic information and (ii) the prediction of molecular information using the transcriptional subtypes as a showcase.

A major strength of our approach is the sample size of the discovery cohort, which is the largest publicly available digital resource for FFPE digital slides in glioblastoma reported to date [22, 44]. This resource comprises 460 GB corresponding to 220,000 individual tiles, including 146,000 tumor tiles. Previous works had already demonstrated the applicability of CNNs for classification and grading of gliomas [11–13]. We here employed a modified and pretrained Xception CNN model, which is relatively lightweight compared to other CNN architectures, while performing on par or better on the ImageNet classification task [33, 48–50]. Recent research efforts have given rise to many different deep learning models that may serve as an efficient backbone for computational pathology tasks [51–53]. The variability of pretrained models can help to find solutions tailored to a wide range of tasks and requirements, and selecting and adapting the most appropriate model typically results in improved performance. While foundation models for computational pathology represent a significant advancement, the full potential of their generalizability across different organs and disease types requires thorough evaluation [54–56].

Our first and foremost result is the identification of a novel histology-based prognostic factor. Even though glioblastoma is known for its extensive inter- and intratumoral heterogeneity at the histological level, as reflected by the term “multiforme” in previous classifications, no histology-based marker had been consistently linked to outcome. Hence, it is exciting to see that the RS-CNN was able to capture clinically meaningful prognostic information in the format of a risk score that can be used to stratify patients into risk groups. At the same time, however, spatial mapping of the risk score allows interpretability in a local micro- and global macroenvironmental context. In our case, high-risk regions were characterized by a simultaneous increase in tumor cell density and decrease in TIL surveillance, both parameters that vary considerably across glioblastoma whole slides and are not easily captured by visual inspections of H&E slides alone [57].

Regarding our second task, the prediction and spatial mapping of transcriptional subtypes, reassuringly, our results grossly support established associations between molecular subtype regions and microenvironmental aspects such as necrosis and TAM infiltration and the mesenchymal subtype [25, 27, 58]. Extending beyond previous work, we demonstrate that also the nuclear morphology and density of the tumor cells differ across subtype-specific regions. Intuitively, cells residing in proneural areas were linked to higher nuclear circularity, potentially reflecting uniform “oligodendroglial or OPC-like” tumor cell shapes and/or admixture of nonneoplastic cells. Likewise, we observed lower cell density in mesenchymal regions that could relate to the presence of necrotic areas or, in the case of proneural regions, to paucicellular infiltration zones. Directly linking histological patterns to these cellular states will be an important next step that requires single-cell transcriptomic data [23].

When ultimately connecting transcriptional subtypes with risk, high-risk regions were only marginally enriched for mesenchymal and classical regions, which is somewhat surprising given that only the mesenchymal subtype had been previously linked with adverse outcome but did not seem to contribute major information to the RS-CNN model [25]. Importantly, however, our TS-CNN was able to predict the presence and distribution of subtypes solely based on H&E slides, which are ubiquitously available as part of any routine diagnostic assessment (also in smaller labs without established molecular workflows), are highly cost-efficient, and save weeks as compared with technically demanding spatially resolved RNA sequencing [59, 60].

We thoroughly validated both the RS-CNN and TS-CNN in an external cohort using unseen digital slides derived from TCGA [44], which resulted in a slightly lower accuracy in the validation set, which was to be expected for 2 reasons. First, the datasets differed in their molecular annotation as for TCGA slides, only the predominant subtype information was available as compared to the subtype-specific probabilities we had for the discovery cohort. Second, in the TCGA cohort, bulk RNA sequencing and digital slides were likely derived from different regions of the same tumor.

Predicting risk and transcriptional subtypes from H&E scans opens up many interesting perspectives for future work. Integrating histological features with spatially resolved transcriptomics will help in understanding how cell identity functionally shapes cell morphology, a concept that can be elegantly extended to further modalities such as spatial proteomics or epigenomics [19, 24, 61–63]. Furthermore, as most spatial molecular profiling techniques are time and cost intensive, they have been mainly applied in research without immediate clinical implications. However, the ability to predict molecular markers directly from H&Es would fuel their translational impact, paving the way toward broad and rapid clinical use of novel biomarkers. Importantly, this concept is not limited to FFPE-derived H&Es but could potentially include H&Es from cryosections as a means to support intraoperative integrated diagnostics [64, 65].

Our study has limitations. First, for internal validation, we performed 5-fold cross-validation instead of using an additional internal test set, which was mostly due to the sample size. Second, even though the high-risk and low-risk groups showed significantly different survival in the external validation cohort, the underlying numerical risk score failed to accurately capture these survival differences upon univariable analysis. Third, the molecular annotation for both cohorts was obtained from bulk sequencing, and it will be important to follow up on our models using datasets that comprise matched H&E slides and spatially resolved sequencing data at single-cell resolution.

## Conclusions

In sum, we present 2 deep learning–based convolutional neural networks that complement the histologic assessment of glioblastoma by adding spatially resolved information on transcriptional subtype and prognostic patient information. The code can be easily adapted to similar problems and is provided under a permissive license.

## Availability of Source Code and Requirements

The code for CNN training is available via GitHub [43]. This includes code for the initial training of CV-folds and corresponding exemplary histological data and clinical annotation. Moreover, we provide a final fully trained predictor as *gbm_predictor.py* that has been trained with the complete discovery dataset and may be used for assessing new digital slides (supported formats are ndpi and svs). Additionally, we also provide QuPath groovy-scripts for the analysis of the tumor microenvironment.

- Project name: GBMatch_CNN
- Project homepage: https://github.com/tovaroe/GBMatch_CNN
- Operating system(s): Platform independent
- Programming language: Python, Groovy (QuPath)
- Other requirements: Python 3.6 or higher, additional dependencies are listed on the project home page; QuPath ≥ 0.3.0
- License: GPL-3.0
- Workflowhub: https://doi.org/10.48546/WORKFLOWHUB.WORKFLOW.883.1
- GBMPredictor is registered as a software application on on SciCrunch (RRID: SCR_025316) and biotools (biotools:gbmpredictor)

## Additional Files

**Supplementary Fig. S1**. Glioblastoma intratumoral heterogeneity. (a) Overview of an H&E-stained section of an FFPE sample. (b) Manual segmentation overlay (necrosis: green overlay, bleeding: red overlay). (c) Cellularity heatmap highlighting heterogeneously distributed, cell-dense tumor areas. (d) Proliferation as measured by Ki-67 antigen expression. (e) Macrophages as CD68-expressing cells surrounding necrosis and in single hotspots. (f) T-lymphocytes (CD8-expressing cells) with similar patchy distribution.

**Supplementary Fig. S2**. (a–e) Boxplots of different immunohistochemical stainings in different predicted TS (all *P*-values calculated with MWU).

**Supplementary Fig. S3**. (a–f) Boxplots of different immunohistochemical stainings by risk class (*P*-values calculated with MWU). (g) Boxplot of the risk scores in perinecrotic/non-perinecrotic tissue (*P*-value calculated with MWU). (h) Kaplan–Meier survival plot using the median risk score of all patients as a cutoff for grouping.

**Supplementary Fig. S4**. UMAPs with different grouping variables.

**Supplementary Table S1**. Number of stained slides included in the analysis of the tumor microenvironment for each region and staining.

**Supplementary Table S2**. Cox multivariable survival model including MGMT status in a subset of 41 patients. HR for age is calculated for each 1-year increase of patient age.

## Abbreviations

CNN: convolutional neural network; FFPE: formalin fixed, paraffin embedded; MWU: Mann-Whitney U; RS-CNN: risk score CNN; TAM: tumor-associated macrophage; TCGA: The Cancer Genome Atlas; TIL: tumor-infiltrating lymphocyte; TS: transcriptional subtype; TS-CNN: transcriptional subtype CNN.

## Author Contributions

Conceptualization: T.R.-P., M.R., B.B., G.L., A.W. Methodology: T.R.-P., K.-H.N., M.R., B.B., G.L., A.W. Formal analysis and investigation: T.R.-P. Writing—original draft preparation: T.R.-P., A.W. Writing—review and editing: all authors. Funding acquisition: T.R.-P., G.L., A.W. Resources: T.R.-P., B.K., J.K., A.W. Supervision: B.B., G.L., A.W.

## Ethics Approval and Consent to Participate

The present study has been approved by the Ethics Committee of the Medical University of Vienna (EK1691-2017) and complies with all relevant ethical, legal, and institutional regulations.

## Supplementary Material

giae057_GIGA-D-23-00317_Original_Submission

giae057_GIGA-D-23-00317_Revision_1

giae057_GIGA-D-23-00317_Revision_2

giae057_Response_to_Reviewer_Comments_Original_Submission

giae057_Response_to_Reviewer_Comments_Revision_1

giae057_Reviewer_1_Report_Original_Submission Hang Chang -- 11/18/2023 Reviewed

giae057_Reviewer_1_Report_Revision_1 Hang Chang -- 5/28/2024 Reviewed

giae057_Reviewer_2_Report_Original_Submission Chris Armit -- 11/20/2023 Reviewed

giae057_Reviewer_3_Report_Original_Submission Junhan Zhao -- 2/13/2024 Reviewed

giae057_Reviewer_3_Report_Revision_1 Junhan Zhao -- 5/24/2024 Reviewed

giae057_Supplemental_Files

## Data Availability

Following recent efforts to make all raw and intermediate annotations publicly available for easy reuse [66], the complete slide scan library, including H&E-stained slides and intermediate annotations such as corresponding tissue segmentations as well as immunohistochemically stained slides, is available online via the GBMatch supplementary website [22, 67]. All preselected image tiles used for training with their corresponding annotations and segmentations for the immunohistochemically stained slides are available via an accompanying Zenodo repository [68]. The external TCGA validation dataset is available via cBioPortal [45] and the GDC Data Portal [46]. Snapshots of our code and other data further supporting this work are openly available in the *GigaScience* repository, GigaDB [69].
