# Supplementary material for: Deep learning links localized digital pathology phenotypes with transcriptional subtype and patient outcome in glioblastoma
Source: Gigascience. 2024 Aug 26;13:giae057. doi: 10.1093/gigascience/giae057 (PMC11345537; doi:10.1093/gigascience/giae057)
Supplement: giae057_GIGA-D-23-00317_Original_Submission [file giae057_giga-d-23-00317_original_submission.pdf]

GigaScience

Deep learning links localized digital pathology phenotypes with transcriptional subtype and patient outcome in glioblastoma

--Manuscript Draft--

**Manuscript Number:**	GIGA-D-23-00317
**Full Title:**	Deep learning links localized digital pathology phenotypes with transcriptional subtype and patient outcome in glioblastoma
**Article Type:**	Research
**Funding Information:**	Österreichischen Akademie der Wissenschaften (DOC 25262) — Mr. Thomas Roetzer-Pejrimovsky<br>Austrian Science Fund (KLI394) — Mrs. Adelheid Woehrer<br>Austrian Science Fund (TAI98B) — Mrs. Adelheid Woehrer<br>Vienna Science and Technology Fund (LS20-034) — Mrs. Adelheid Woehrer<br>Vienna Science and Technology Fund (LS20-065) — Mr. Georg Langs

Abstract:

Background

Deep-learning has revolutionized medical image analysis in cancer pathology, where it had a substantial clinical impact by supporting the diagnosis and prognostic rating of cancer. Among the first available digital resources in the field of brain cancer is glioblastoma, the most common and fatal brain cancer. At the histologic level, glioblastoma is characterized by abundant phenotypic variability that is poorly linked with patient prognosis. At the transcriptional level, three molecular subtypes are distinguished with mesenchymal-subtype tumors being associated with increased immune cell infiltration and worse outcome.

Results

We address genotype-phenotype correlations by applying an Xception convolutional neural network to a discovery set of 276 digital H&E slides with molecular subtype annotation, and an independent TCGA-based validation cohort of 178 cases. Using this approach, we achieve high accuracy in H&E-based mapping of molecular subtypes (AUC for classical, mesenchymal, proneural = 0.84, 0.81, and 0.71, respectively; $p < 0.001$) and regions associated with worse outcome (univariable survival model $p < 0.001$, multivariable $p = 0.01$). The latter were characterized by higher tumor cell density ($p < 0.001$), phenotypic variability of tumor cells ($p < 0.001$), and decreased T-cell infiltration ($p = 0.017$).

Conclusions

We introduce a novel CNN architecture for glioblastoma digital slides that accurately maps the spatial distribution of transcriptional subtypes and regions predictive of worse outcome, thereby showcasing the relevance of AI-enabled image mining in brain cancer.

**Corresponding Author:**	Thomas Roetzer-Pejrimovsky, MD<br>Medical University of Vienna<br>Vienna, Vienna AUSTRIA
**Corresponding Author Secondary Information:**	
**Corresponding Author's Institution:**	Medical University of Vienna
**Corresponding Author's Secondary Institution:**	

Powered by Editorial Manager® and ProduXion Manager® from Aries Systems Corporation

**First Author:**	Thomas Roetzer-Pejrimovsky, MD
**First Author Secondary Information:**	
**Order of Authors:**	Thomas Roetzer-Pejrimovsky, MD
	Karl-Heinz Nenning
	Barbara Kiesel
	Johanna Klughammer
	Martin Rajchl
	Bernhard Baumann
	Georg Langs
	Adelheid Woehrer
**Order of Authors Secondary Information:**	
**Additional Information:**	
**Question**	**Response**
Are you submitting this manuscript to a special series or article collection?	No
**Experimental design and statistics**<br><br>Full details of the experimental design and statistical methods used should be given in the Methods section, as detailed in our Minimum Standards Reporting Checklist. Information essential to interpreting the data presented should be made available in the figure legends.<br><br>Have you included all the information requested in your manuscript?	Yes
**Resources**<br><br>A description of all resources used, including antibodies, cell lines, animals and software tools, with enough information to allow them to be uniquely identified, should be included in the Methods section. Authors are strongly encouraged to cite Research Resource Identifiers (RRIDs) for antibodies, model organisms and tools, where possible.<br><br>Have you included the information requested as detailed in our Minimum Standards Reporting Checklist?	Yes

Powered by Editorial Manager® and ProduXion Manager® from Aries Systems Corporation

**Availability of data and materials**<br><br>All datasets and code on which the conclusions of the paper rely must be either included in your submission or deposited in publicly available repositories (where available and ethically appropriate), referencing such data using a unique identifier in the references and in the "Availability of Data and Materials" section of your manuscript.<br><br>Have you have met the above requirement as detailed in our Minimum Standards Reporting Checklist?	Yes

Powered by Editorial Manager® and ProduXion Manager® from Aries Systems Corporation

Manuscript
Click here to access/download;Manuscript;Manuscript.docx

Deep learning links localized digital pathology phenotypes with transcriptional subtype and patient outcome in glioblastoma

Thomas Roetzer-Pejrimovsky[1,2], Karl-Heinz Nenning[3,4], Barbara Kiesel[5], Johanna Klughammer[6], Martin Rajchl[7], Bernhard Baumann[8], Georg Langs[4], Adelheid Woehrer[1,2]

1 Division of Neuropathology and Neurochemistry, Department of Neurology, Medical University of Vienna, Vienna, Austria.

2 Comprehensive Center for Clinical Neurosciences and Mental Health, Medical University of Vienna, Vienna, Austria

3 Center for Biomedical Imaging and Neuromodulation, Nathan Kline Institute, Orangeburg, NY, USA

4 Department of Biomedical Imaging and Image-Guided Therapy, Computational Imaging Research Lab, Medical University of Vienna, Vienna, Austria.

5 Department of Neurosurgery, Medical University of Vienna, Vienna, Austria.

6 Gene Center and Department of Biochemistry, Ludwig-Maximilians-Universität München, Munich, Germany.

7 Department of Computing and Medicine, Imperial College London, London, U.K.

8 Center for Medical Physics and Biomedical Engineering, Medical University of Vienna, Vienna, Austria.

Corresponding author:

Georg Langs: georg.langs@meduniwien.ac.at

Abstract

Background:

Deep-learning has revolutionized medical image analysis in cancer pathology, where it had a substantial clinical impact by supporting the diagnosis and prognostic rating of cancer. Among the first available digital resources in the field of brain cancer is glioblastoma, the most common and fatal brain cancer. At the histologic level, glioblastoma is characterized by abundant phenotypic variability that is poorly linked with patient prognosis. At the transcriptional level, three molecular subtypes are distinguished with mesenchymal-subtype tumors being associated with increased immune cell infiltration and worse outcome.

Results:

We address genotype-phenotype correlations by applying an Xception convolutional neural network to a discovery set of 276 digital H&E slides with molecular subtype annotation, and an independent TCGA-based validation cohort of 178 cases. Using this approach, we achieve high accuracy in H&E-based mapping of molecular subtypes (AUC for classical, mesenchymal, proneural = 0.84, 0.81, and 0.71, respectively; $p < 0.001$) and regions associated with worse outcome (univariable survival model $p < 0.001$, multivariable $p = 0.01$). The latter were characterized by higher tumor cell density ($p < 0.001$), phenotypic variability of tumor cells ($p < 0.001$), and decreased T-cell infiltration ($p = 0.017$).

Conclusions:

We introduce a novel CNN architecture for glioblastoma digital slides that accurately maps the spatial distribution of transcriptional subtypes and regions predictive of worse outcome, thereby showcasing the relevance of AI-enabled image mining in brain cancer.

Key words

Glioblastoma, deep learning, histology, digital pathology, risk score

Background

Computer vision has undergone a revolution in recent years, which was in large parts driven by the development of convolutional neural networks (CNNs) [1–3]. In digital pathology, major achievements included the precise segmentation of individual cells [4–7], histologic structures [8,9] and tumor tissues [10]. In glioma, so far, CNNs have been employed for tumor typing, grading, and prognostic rating [11–13]. Still, the links between histologic phenotypes and underlying genotypes remain insufficiently understood; a gap, which could be addressed using CNNs [2,3].

Glioblastoma is the most common and fatal brain tumor in adults [14]. Prognostic factors include patient age, clinical performance, tumor location and resectability, DNA methylation at the MGMT gene promoter, and receipt of multimodal treatment [15–17]. So far, no histology-based prognostic biomarker is available.

At the histologic level, glioblastoma is characterized by extensive within- and across-tumor variability ranging from small-celled to monstro-cellular and sarcomatous cells with recurrent formation of palisades around necroses and Scherer's secondary structures at the invasive front. Also, the composition of the microenvironment varies in space and time with bone marrow-derived macrophages being abundant in necrotic regions, brain-resident microglia within and surrounding tumor regions, as well as scattered lymphocytes in perivascular arrangements.

At the level of tumor biology, glioblastoma is characterized by complex genetic aberrations and transcriptional plasticity with considerable spatial and temporal variability (Figure S1) [18–20]. At the bulk-level, three transcriptional subtypes were defined, i.e., classical, mesenchymal and proneural, each being enriched for genetic alterations and microenvironmental factors [21]. Importantly, previous efforts to explore the spatial distribution of the transcriptional subtypes pointed towards associations between the proneural subtype and invasive edges with enhanced neuronal signaling, as well as the

mesenchymal subtype and perinecrotic areas with denser immune cell infiltration [22–24]. However, despite their biologic relevance, their translation into routine clinical assessments based on formalin-fixed paraffin-embedded (FFPE) tissues was largely prevented by the limited availability of FFPE-based spatial transcriptomics technology. Hence, a computational solution that enables their accurate prediction in spatial context based on ubiquitously available, cost-efficient H&E-stains would fuel their translation and clinical applicability.

Here, we introduce an end-to-end CNN that generates a histology-based risk score to estimate patient prognosis (RS-CNN) and maps the spatial distribution of transcriptional subtypes (TS-CNN, Fig. 1).

Materials and methods

Patient cohort

We leveraged an existing longitudinal glioblastoma patient cohort comprising matched histological and DNA methylation-derived transcriptional subtypes at time of first and second surgery [19].

A total of 276 patients with digital histology and outcome data were included (table 1, *discovery cohort*) to train the *RS-CNN* using overall survival as a label. For 189 tumors, also transcriptional subtype information was available (table 1, *TS subcohort*), including the admixture of the different subtypes (summing up to 100%) which was used as ground truth for training [19]. Samples with at least 70% contribution by a given subtype were allocated to this subtype (e.g. classical-predominant, proneural-predominant, mesenchymal-predominant). Both the entire discovery cohort and the TS subcohort featured a similar age range and female-to-male ratio. However, the TS cohort was slightly biased towards an increased receipt of temozolomide-based radiochemotherapy and prolonged survival. We

ultimately split each cohort into five equally large folds with comparable characteristics for internal 5-fold cross validation.

		Discovery cohort	TS subcohort
**Number of patients**		276	189
**Median Age [IQR]**		63.0 [53.8 - 70.5] years	62.0 [52.0 - 68.0] years
**F:m ratio**		0.62 (106:170)	0.64 (74:115)
**Combined radiochemotherapy (TMZ)**		205 (74.3 %)	153 (81.0 %)
**Median overall survival**		1.16 years	1.51 years
**Alive at last follow-up**		7 (2.54 %)	7 (3.7 %)
**TS**	**Classical predominant**	-	34 (17.99 %)
	**Mesenchymal predominant**	-	50 (26.46 %)
	**Proneural predominant**	-	21 (11.11 %)
	**Mixed**	-	84 (44.44 %)

Table 1. Demographics of the discovery cohort and the TS subcohort. The whole discovery cohort was used for risk score prediction. The TS subcohort was used for TS prediction. CNN: convolutional neural network, IQR: interquartile range, TMZ: temozolomide, TS: Transcriptional subtype

Handling of digital slides

H&E sections were digitized using a Hamamatsu NanoZoomer 2.0 HT slide scanner. On each digital slide, necrosis, preexisting brain parenchyma, bleeding, scar tissue and deformed tissue had been manually segmented by a board-certified neuropathologist (A.W.) using the ndp.view2-built-in annotation tool. The remaining areas were assigned to tumor areas. Each digital slide was converted to multiple (i.e. 6 to 2257) 1024x1024 pixel tiles at 20x magnification (456 px / µm) with 64px overlap using a custom MATLAB script (MATLAB R2017b, MathWorks) [25,26]. An accompanying spreadsheet contained the coordinates of each tile with the relative areas per segmented region. We defined perinecrotic regions as image tiles containing both tumor tissue and necrosis. Similarly, we defined the infiltration

zone as tiles containing both tumor and preexisting tissue. For classifier training, only tiles with > 50% tumor tissue were kept. Patients with less than 50 different tiles had been excluded from further analysis. For training, we performed random cropping to 512x512 px and automated data augmentation with the H&E-specific algorithm of Faryna at runtime [27].

CNN architecture

We used TensorFlow 2.1.0 / keras for developing our deep learning pipeline [28]. As a base model, we used an Xception model [29] pre-trained on ImageNet available via the keras model applications [30]. The input consisted of a (randomly sampled) WSI tile and no other information was introduced to the model. We froze all weights and added an extra layer depending on the target. For TS prediction, we added a fully connected 3-neuron layer with softmax activation. The TS target consisted of the three probabilities for each of the transcriptional subtypes. The mean squared error was backpropagated to update the weights. For risk score prediction, we added a single one-neuron layer with a linear activation function. The negative log likelihood was used as a loss function and was backpropagated to update the weights in a similar approach as Mobadersany et al. [11]. We adapted keras' DataFrameIterator such that for each new cycle through the digital slides, a new random image tile was selected per patient, randomly cropped and augmented. The TS-CNN and RS-CNN were trained independently of each other. Each model was first trained for 25 epochs with a custom 150 steps per epoch (for better performance) and a batch size of 64. We used the Adam optimizer with a learning rate of 0.001 and exponential learning rate decay every 400 steps at a decay rate of 0.9. For finetuning, the last 2 convolutional layers (4,741,632 of 20,861,480 parameters) of the Xception model were set trainable and the model was trained for 10 further epochs with 150 steps per epoch and a batch size of 64. Again, we used the Adam optimizer with a learning rate of 0.0001 and exponential learning rate decay every 400 steps at a decay rate of 0.8. During training, at the start of each fold 20 random batches were loaded into memory for validation. At the end of

each epoch, the mean squared error (for TS prediction) or the c-index (for survival prediction) were calculated for the validation batches to keep track of the model performance.

We used 5-fold cross validation during model training. For the final validation, we let the trained models predict all validation tiles (with center crop to 512x512 px and no augmentation). The RS predictions were z-scored, the TS predictions were taken as they were, then all validation set predictions were concatenated into a single spreadsheet for further statistical analysis.

H&E mapping

To visualize the spatial distribution of the predicted targets directly in the digital slides, we performed the predictions on a set of windows covering the entire digital slide. We then mapped the predictions to the coordinates of those windows. Thereby, heatmaps were plotted in triplets representing the three transcriptional subtypes, or as a single map depicting the risk score [31].

Statistical analysis

Statistical analysis was conducted in Python 3.8.5. We performed permutation tests by label shuffling to compare our predicted risk scores to random guesses. To calculate p-values determining the significance of the RS and TS predictions, we performed label shuffling to generate a null distribution. Mann-Whitney-U and Wilcoxon tests were calculated with scipy [32]. Kaplan-Meier survival analysis and Cox proportional hazards models were performed using lifelines [33]. Harrel's c-index was calculated using sksurv [34]. Figures were drawn using matplotlib [35] and seaborn [31]. The confusion matrix and roc analysis were performed using sklearn [36]. To compare RS with TS scores, we assigned each tile to the subtype displaying the highest predicted score (winner-takes-all). Based on that annotation, we then calculated the mean risk score for each transcriptional subtype.

For UMAP plotting, we first concatenated the outputs of the penultimate CNN layers of all models obtaining 20,480 features for each image tile. We then used the umap package to plot UMAPs.

Characterization of the tumor microenvironment

We used QuPath 0.3.0 [37] for the following steps. To showcase the within-tumor histological variability, we used the inbuilt “density map” function. We first performed “fast cell counts” on the H&E digital slides to obtain overall cellularity (i.e., cell density) and circularity (i.e., cell *roundness*). The tumor cell proliferation, tumor-associated macrophages (TAM) and lymphocytes (TIL) density maps were calculated from Ki-67-, CD68-, CD163-, HLA-DR- and CD8-stained digital slides using “positive cell detection”. The immunohistochemical stainings were performed on a Dako autostainer system with the following antibodies: CD3 (Thermo Scientific no. RM-9107-S1, 1:200), CD8 (Dako Cytomation no. M7103, 1:100), CD163 (Novocastra no. NCL-L-CD163, 1:1000), CD68 (Dako Cytomation no. M0814, 1:5000), HLA-DR (Dako Cytomation no. M0775, 1:400), Ki-67 (MIB-1) (Dako Cytomation no. M7240, 1:200), and CD34 (Novocastra no. NCL-l-END, 1:100). [19] To link TAM and TIL densities with transcriptional subtypes and risk tiles, we manually segmented the respective regions on neighboring digital slides (where available and adequate) (table S1). After using “positive cell detection”, we counted all stained cells in each region and divided this count by the respective area to obtain the number of stained cells per mm^2. For HLA-DR and CD34 we calculated the relative stained area in a similar fashion. Thus, we obtained a quantitative characterization of the tumor microenvironment per slide/patient. We calculated summary statistics on this slide/patient level to compare the different transcriptional subtype regions and high/low risk regions. The QuPath script with the specific parameters is provided in the appendix.

External validation using TCGA data

After successful internal validation, we re-trained our CNN models on our complete discovery dataset using the same parameters as previously stated. We then downloaded the clinical annotation for the TCGA glioblastoma cohort published by Brennan et al. [38] from cBioPortal [39]. We screened the GDC Data Portal for available diagnostic slides and downloaded them using the GDC Data Transfer Tool [40]. To match the inclusion criteria of our training cohort, we excluded slides of suboptimal quality (due to excessive artifacts, poor staining, or non-FFPE H&E slides) and tumors with mutant or unknown IDH status. We manually segmented the tumor tissue and infiltration zone in concordance to the discovery cohort. We then applied the RS and TS CNNs to the validation set. We averaged the subtype predictions over all image tiles and let the highest subtype score determine the predicted subtype per sample. We considered samples with a mismatch between predicted subtype and TCGA bulk sequencing derived subtype as misclassified. Moreover, patients were assigned to two risk groups, depending on the fraction of *high risk* (z-score > 1) tiles (cut-off 25%). High-risk samples of patients who survived > 18 months and low-risk samples of patients with < 12 months survival were considered misclassified.

Analyses

H&E-based mapping of transcriptional subtypes

The accuracy for predicting the predominant subtype was 66.7 % as compared to a random guess accuracy of 38.67 % [+/- 0.4 %] (p < 0.001, permutation test, Figure 2a, b). The mean squared error was 0.08 in the validation folds as compared to 0.11 [+/- 0.003] for random predictions (p < 0.001, permutation test). Overall, the spatial distribution of subtypes aligned well with the segmented tumor regions (Figure 2c and 2d) both upon visual inspection of the heatmaps as well as upon quantification at the cohort-level. Precisely, median predictive

scores were significantly higher for proneural in the infiltration zone ($p < 0.001$, MWU, Figure 2e), and for mesenchymal in perinecrotic areas ($p = 0.021$, MWU Figure 2f). Likewise, a significantly higher cellularity and tendency to larger fractions of cycling cells were found in classical areas ($p < 0.001$, Wilcoxon test, Figure 2g & $p < 0.05$, MWU). At the individual cell level, nuclear circularity was highest in proneural and lowest in mesenchymal areas (all $p < 0.001$, Wilcoxon, Figure 2h). Ultimately, we found increased infiltration by CD68+, CD163+ and HLA-DR+ myeloid cells and CD3+, CD8+ TILs in mesenchymal regions (all $p < 0.006$, MWU, Figure 2i). Likewise, areas covered by CD34+ vessels were enriched in mesenchymal as compared to proneural ($p < 0.01$, MWU) or classical ($p = 0.02$, MWU) regions.

	Classical	Mesenchymal	Proneural	p-value
**Cellularity (per mm²)**	6146 [4800 - 7574]	5484 [4046 - 6353]	5321 [3897 - 6833]	p < 0.001
**Circularity**	0.79 [0.78 - 0.81]	0.78 [0.77 - 0.8]	0.8 [0.78 - 0.82]	p < 0.001
**CD163^{+} cells (per mm²)**	9 [1 - 59]	348 [101 - 871]	37 [8 - 91]	p = 0.027
**CD3^{+} cells (per mm²)**	34 [17 - 82]	129 [53 - 310]	25 [15 - 52]	p = 0.006
**CD68^{+} cells (per mm²)**	96 [23 - 277]	243 [98 - 573]	72 [24 - 194]	p < 0.001
**CD8^{+} cells (per mm²)**	10 [5 - 20]	36 [19 - 81]	9 [4 - 18]	p < 0.001
**MIB^{+} cells (per mm²)**	290 [128 - 630]	108 [59 - 214]	60 [26 - 581]	p < 0.001
**CD34**	4 % [3 - 6]	5 % [4 - 11]	2 % [1 - 4]	p < 0.001
**HLA-DR**	2 % [0 - 9]	8 % [4 - 18]	1 % [0 - 3]	p < 0.001

Table 2. Comparison of cellular phenotype and immunohistochemical parameters [median, IQR] between different predicted TS. Given values represent a summary statistic over all slides and the whole respective subtype region (if present on their digital slide) was evaluated for each patient. The p-values were calculated using the Kruskal-Wallis H-test.

H&E-based risk score prediction

The risk score prediction model (*RS-CNN*) was trained end-to-end on histological images alone using the Cox loss function (negative log-likelihood), which yielded a single risk score as output. To obtain patient-level predictions, the predicted scores per tile were normalized (z-scored) across the entire cohort and aggregated using the arithmetic mean. Additionally, the fraction of high-risk tiles (z-scored risk > 1) was calculated per digital slide and their distribution plotted as a heatmap (Fig. 3a). In the validation folds, the risk scores were strongly associated with survival upon univariable ($p < 0.001$, Fig. 3b) and multivariable analyses ($p = 0.013$, table 3).

	**HR**	**p-value**
**Age**	1.025 (1.015 - 1.036)	< 0.001
**Male sex**	1.14 (0.88 - 1.48)	0.331
**Radiochemotherapy (TMZ)**	0.43 (0.32 - 0.58)	< 0.001
**RS CNN**	1.32 (1.06 - 1.65)	0.013

Table 3. Cox multivariable survival model. HR for age is calculated for each 1-year increase of patient age.

The median risk score was significantly lower in infiltration zones (Fig. 3e, $p = 0.009$, MWU) and not enhanced in perinecrotic areas ($p=0.446$, MWU). High-risk areas were characterized by higher cellularity ($p < 0.001$, Wilcoxon), decreased nuclear circularity (reflecting polymorphous nuclei, $p < 0.001$, Wilcoxon), fewer CD8+ cells ($p = 0.017$, MWU), and a trend towards fewer CD3+ cells ($p = 0.06$, MWU). There was no significant difference in CD68+, CD163+ or HLA-DR+ myeloid cell density (p = 0.13, 0.435, and 0.25, respectively, MWU), the fraction of cycling cells ($p = 0.19$, MWU), and microvessel density ($p = 0.31$, MWU).

	High risk	Low risk	p-value
**Cellularity (per mm²)**	5877 [4336 - 7302]	5524 [3885 - 6891]	$p < 0.001$
**Circularity**	0.78 [0.75 - 0.8]	0.79 [0.77 - 0.81]	$p < 0.001$
**$CD163^+$ cells (per mm²)**	46 [8 - 234]	27 [7 - 366]	$p = 0.35$
**$CD3^+$ cells (per mm²)**	33 [16 - 70]	38 [22 - 221]	$p = 0.063$
**$CD68^+$ cells (per mm²)**	108 [26 - 219]	157 [45 - 274]	$p = 0.127$
**$CD8^+$ cells (per mm²)**	9 [3 - 23]	16 [8 - 58]	$p = 0.017$
**MIB^+ cells (per mm²)**	248 [45 - 634]	138 [48 - 340]	$p = 0.191$
**CD34**	3 [2 - 5] %	4 [2 - 6] %	$p = 0.306$
**HLA-DR**	3 [0 - 9] %	1 [0 - 9] %	$p = 0.25$

Table 4. Comparative analysis between high- and low-risk regions across histological and immunohistochemical parameters [median, IQR]. P-values were calculated using the Wilcoxon signed-rank test (Cellularity, Circularity) and the Mann-Whitney U test (immunohistochemical stainings), respectively.

Integration of risk scores with transcriptional subtypes

Ultimately, we aimed to link predicted risk scores with TS scores. Dimensionality reduction of aggregated TS and RS features resulted in one continuous feature space with smaller peripheral clusters that mostly represented individual patients. Still, also regional clusters relating to gross histologic features such as cellularity or nuclear circularity emerged (Fig 4a&b).

Furthermore, we calculated the mean predicted risk score for each of the transcriptional subtypes per slide, which resulted in signficantly higher risk scores in classical and mesenchymal than in proneural areas (Fig 4c, $p = 0.001$ and 0.02, respectively, Wilcoxon).

External validation in TCGA datasets

Finally, we sought to validate the performance of our models in an independent TCGA dataset (Fig 5a). Applying the previously defined cut-off of 25% high risk tiles, resulted in a statistically significant separation of survival curves (p = 0.003, logrank test, Figure 5b). Of note, 14% of the validation set were assigned to the high-risk group, as compared to 18% in the discovery cohort. Harrel's c-index was 0.52 and the mean risk score was not significantly associated with survival (Cox regression univariable HR = 1.4 +/- 0.25, p = 0.16; multivariable HR = 1.2 +/- 0.18, p = 0.31). In parallel, the accuracy for predicting the transcriptional subtypes was 56.2% compared to a random guess accuracy of 34.3% [+/- 0.4 %] in the validation set (p < 0.001, permutation test, Figure 5c & d). Interestingly, the accuracy was highest for predicting the mesenchymal subtype (AUC = 0.746) as compared to the classical (AUC = 0.704) and proneural (AUC = 0.697) subtypes.

To better understand the potential drawbacks and pitfalls of the trained CNNs, we specifically looked at misclassified samples (Figure 6). Overall, out of 178 total samples, 78 displayed misclassified transcriptional subtypes, 3 were misclassified as high-risk and 57 were misclassified and low-risk.For 28 samples, both the transcriptional subtype and survival were misclassified. We found that many (29.5%) subtype misclassifications were "near correct", i.e., the difference between the true subtype score and the predicted subtype score was < 0.01. Upon qualitative assessment of the misclassified cases, we further found that many samples had relatively little tumor tissue.

Discussion

In the present study, we leverage deep learning on digital glioblastoma slides to address two relevant applications: 1. the mining of subvisual histological patterns for prognostic information, and 2. the prediction of molecular information using the transcriptional subtypes as a showcase.

A major strength of our approach is the sample size of the discovery cohort, which is the largest publicly available digital resource for FFPE digital slides in glioblastoma reported to date [19,38]. This resource comprises 460GB corresponding to 220,000 individual tiles including 146,000 tumor tiles. Previous works had already demonstrated the applicability of CNNs for classification and grading of gliomas [11–13]. We here used a pre-trained Xception CNN model, which is relatively lightweight compared to other CNN architectures while performing on par or better on the ImageNet classification task [29,41–43].

Our first and foremost result is the identification of a novel histology-based prognostic factor. Even though glioblastoma is known for its extensive inter- and intratumoral heterogeneity at the histological level as reflected by the term “multiforme” in previous classifications, no histology-based marker had been consistently linked to outcome. Hence, it is exciting to see that the RS-CNN was able to capture clinically meaningful prognostic information in the format of a risk score that can be used to stratify patients into risk groups. At the same time, however, spatial mapping of the risk score allows interpretability in local micro- and global macroenvironmental context. In our case, high-risk regions were characterized by a simultaneous increase in tumor cell density and decrease in TIL surveillance, both parameters that vary considerably across glioblastoma whole slides and are not easily captured by visual inspections of H&E slides alone [44].

Regarding our second task, the prediction and spatial mapping of transcriptional subtypes, reassuringly, our results grossly support established associations between molecular subtype regions and microenvironmental aspects such as necrosis and TAM infiltration and the mesenchymal subtype [21,23,45]. Extending beyond previous work, we demonstrate

that also the nuclear morphology and density of the tumor cells differ across subtype-specific regions. Intuitively, cells residing in proneural areas were linked to higher nuclear circularity potentially reflecting uniform “oligodendroglial or OPC-like” tumor cell shapes and/or admixture of non-neoplastic cells. Likewise, we observed lower cell density in mesenchymal regions that could relate to the presence of necrotic areas or in case of proneural regions to paucicellular infiltration zones. Directly linking histological patterns to these cellular states will be an important next step that requires single-cell transcriptomic data [20].

When ultimately connecting transcriptional subtypes with risk, high risk regions were only marginally enriched for mesenchymal and classical regions, which is somewhat surprising given that only the mesenchymal subtype had been previously linked with adverse outcome but did not seem to contribute major information to the RS CNN model [21]. Importantly, however, our TS CNN was able to predict the presence and distribution of subtypes solely based on H&E slides, which are ubiquitously available as part of any routine diagnostic assessment (also in smaller labs without established molecular workflows), highly cost-efficient, and save weeks as compared with technically demanding spatially-resolved RNA-sequencing [46,47].

We thoroughly validated both the RS-CNN and TS-CNN in an external cohort using unseen digital slides derived from TCGA [38], which resulted in a slightly lower accuracy in the validation set, which was to be expected for two reasons. First, the datasets differed in their molecular annotation as for TCGA slides only the predominant subtype information was available as compared to the subtype-specific probabilities we had for the discovery cohort. Second, in the TCGA cohort, bulk RNA-sequencing and digital slides were likely derived from different regions of the same tumor.

Our study has limitations. First, for internal validation we performed 5-fold cross validation instead of using an additional internal test set, which was mostly due to the sample size. Second, even though the high risk and low risk groups showed significantly different survival in the external validation cohort, the underlying numerical risk score failed to accurately capture these survival differences upon univariable analysis. Third, the molecular annotation

for both cohorts was obtained from bulk sequencing, and it will be important to follow up on our models using datasets that comprise matched H&E slides and spatially-resolved sequencing data at single cell resolution.

Conclusions

In sum, we present two deep learning-based convolutional neural networks that complement the histologic assessment of glioblastoma by adding spatially-resolved information on transcriptional subtype and prognostic patient information. The code can be easily adapted to similar problems and is provided under a permissive license.

Availability of Source Code and Requirements

The code for CNN training is available via github [48]. This includes code for the initial training of CV-folds and corresponding exemplary histological data and clinical annotation. Moreover, we provide a final fully trained predictor as *gbm_predictor.py* that has been trained with the complete discovery dataset and may be used for assessing new digital slides (supported formats are ndpi and svs). Additionally, we also provide QuPath groovy-scripts for the analysis of the tumor microenvironment.

- Project name: GBMatch_CNN
- Project home page: https://github.com/tovaroe/GBMatch_CNN
- Operating system(s): Platform independent
- Programming language: Python, Groovy (QuPath)
- Other requirements: Python 3.6 or higher, additional dependencies are listed on the project home page; QuPath >= 0.3.0
- License: GPL-3.0

Data Availability

The complete slide scan library, including H&E stained slides and corresponding tissue segmentations as well as immunohistochemically stained slides, is available online via the GBMatch supplementary website [19,49]. All pre-selected image tiles used for training and segmentations for the immunohistochemically stained slides are available via an accompanying zenodo repository [50]. The external TCGA validation dataset is available via cBioPortal [39] and the GDC Data Portal [40].

Figure Captions

Fig. 1 Approach & network architecture. Digital slides were manually segmented (green: necrosis, red: bleeding, gray: pre-existing tissue) and smaller image tiles comprising mainly/only tumor tissue and infiltration zone were exported. For model training, extensive data augmentation was performed. The deep learning framework consisted of a pre-trained Xception CNN, in which the ultimate layer was replaced by a 3-neuron-layer (for transcriptional subtype prediction, *TS-CNN*) or a 1-neuron-layer (for survival prediction, *RS-CNN*). We used only histological image tiles as input to the model and no other information was introduced to either model.

Fig. 2 Prediction of the transcriptional subtypes (TS). (a) Receiver operating characteristic (ROC) curves and area-under-the-curve per TS (b) confusion matrix for the prediction of TS in samples with a predominant TS. (c) One representative sample with three heat-maps depicting TS scores at the whole slide-level and density maps for cell density, CD68+ TAMs, CD8+ TILs, and Ki67+ cycling cells (d)-(i) Correlative analyses between different histological aspects and TS scores. Of note, single outliers in panels (i) and (j) are not shown for better illustration.

Fig. 3 Prediction of the risk score. (a) Risk maps and corresponding H&E slides of two representative cases. Higher risk scores are depicted in a red hue and lower risk scores in a blue hue. Higher-magnification images are given for the numbered regions of the WSI (top row: low-risk regions,

bottom row: high-risk regions). (b) Kaplan-Meier plot stratified at 25% high-risk tiles ($p < 0.001$, log rank test, Harrel's c-index = 0.6) (c)-(f) Box plots depicting associations between risk scores and selected histological and immunohistochemical aspects.

Fig. 4 Feature landscape and integration of risk and transcriptional subtype scores. (a) UMAP projection of all features with highlighted regions I-III. In the leftmost UMAP, image tiles from three exemplary patients are highlighted in blue, green and orange, respectively and all other patients are plotted in gray. (b) Representative histological images corresponding to regions I-III with (I) corresponding to infiltration zone, (II) monstro-sarcomatoid phenotypes and (III) round-cell & cell-dense regions. (c) Correlation between risk and transcriptional subtype prediction.

Fig. 5 External validation in TCGA data [38]. (a) Inclusion flowchart to match inclusion criteria for the training cohort. (b) Kaplan-Meier plot stratified at content of 25% high-risk tiles ($p = 0.003$, logrank test), (c) ROC and AUC values per TS, (d) confusion matrix with TS prediction accuracy.

Fig. 6 Misclassified TCGA samples. (a) Patient with > 18 months survival and mesenchymal subtype, that was misclassified as high risk and classical subtype. (b) Patient with 3.9 months survival and classical subtype that was misclassified as proneural subtype.
Green segmentations on the H&E correspond to the manually segmented tumor tissue, upon which the CNN prediction was based.

Declarations

List of abbreviations

CNN: Convolutional neural network

FFPE: Formalin-fixed paraffin-embedded

RS-CNN: Risk score CNN

TAM: Tumor-associated macrophages

TCGA: The Cancer Genome Atlas

TIL: Tumor-infiltrating lymphocytes

TS: Transcriptional subtype

TS-CNN: Transcriptional subtype CNN

Ethics approval and consent to participate

The present study has been approved by the Ethics Committee of the Medical University of Vienna (EK1691-2017) and complies with all relevant ethical, legal and institutional regulations.

Competing interests

GL is chief scientist at contextflow GmbH. The other authors declare no competing interests.

Funding

This work was supported by the Austrian Science Fund projects KLI394 and TAI98B to AW. Thomas Roetzer-Pejrimovsky is a recipient of a DOC Fellowship (25262) of the Austrian Academy of Sciences at the Division of Neuropathology and Neurochemistry, Department of Neurology, Medical University of Vienna. Parts of the computational work and digital resources were supported by the Vienna Science and Technology Fund (WWTF) Project No. LS20-034 to AW and Project No. LS20-065 to GL.

Authors’ contributions

Conceptualization: TRP, MR, BB, GL, AW; Methodology: TRP, KHN, MR, BB, GL, AW; Formal analysis and investigation: TRP; Writing - original draft preparation: TRP, AW; Writing - review and editing: all authors; Funding acquisition: TRP, GL, AW; Resources: TRP, BK, JK, AW; Supervision: BB, GL, AW.

Acknowledgements

We thank Christoph Bock for data support. We thank NVIDIA for the donation of a TITAN Xp GPU.

References

1. LeCun Y, Bengio Y, Hinton G. Deep learning. *Nature*. 2015; doi: 10.1038/nature14539.

2. Jiang Y, Yang M, Wang S, Li X, Sun Y. Emerging role of deep learning-based artificial intelligence in tumor pathology. *Cancer Commun*. 2020; doi: 10.1002/cac2.12012.

3. Chen RJ, Lu MY, Williamson DFK, Chen TY, Lipkova J, Noor Z, et al.. Pan-cancer integrative histology-genomic analysis via multimodal deep learning. *Cancer Cell*. 2022; doi: 10.1016/j.ccell.2022.07.004.

4. Lal S, Das D, Alabhya K, Kanfade A, Kumar A, Kini J. NucleiSegNet: Robust deep learning architecture for the nuclei segmentation of liver cancer histopathology images. *Comput Biol Med*. 2021; doi: 10.1016/j.compbiomed.2020.104075.

5. Falk T, Mai D, Bensch R, Çiçek Ö, Abdulkadir A, Marrakchi Y, et al.. U-Net: deep learning for cell counting, detection, and morphometry. *Nat Methods*. 2019; doi: 10.1038/s41592-018-0261-2.

6. Sirinukunwattana K, Ahmed Raza SE, Yee-Wah Tsang, Snead DRJ, Cree IA, Rajpoot NM. Locality Sensitive Deep Learning for Detection and Classification of Nuclei in Routine Colon Cancer Histology Images. *IEEE Trans Med Imaging*. 2016; doi: 10.1109/TMI.2016.2525803.

7. Naylor P, Lae M, Reyal F, Walter T. Segmentation of Nuclei in Histopathology Images by Deep Regression of the Distance Map. *IEEE Trans Med Imaging*. 2019; doi: 10.1109/TMI.2018.2865709.

8. Hermsen M, de Bel T, den Boer M, Steenbergen EJ, Kers J, Florquin S, et al.. Deep Learning-Based Histopathologic Assessment of Kidney Tissue. *J Am Soc Nephrol*. 2019; doi: 10.1681/ASN.2019020144.

9. Graham S, Chen H, Gamper J, Dou Q, Heng P-A, Snead D, et al.. MILD-Net: Minimal information loss dilated network for gland instance segmentation in colon histology images. *Med Image Anal*. 2019; doi: 10.1016/j.media.2018.12.001.

10. Ehteshami Bejnordi B, Veta M, Johannes van Diest P, van Ginneken B, Karssemeijer N, Litjens G, et al.. Diagnostic Assessment of Deep Learning Algorithms for Detection of Lymph Node Metastases in Women With Breast Cancer. *JAMA*. 2017; doi: 10.1001/jama.2017.14585.

11. Mobadersany P, Yousefi S, Amgad M, Gutman DA, Barnholtz-Sloan JS, Velázquez Vega JE, et al.. Predicting cancer outcomes from histology and genomics using convolutional networks. *Proc Natl Acad Sci U S A*. 2018; doi: 10.1073/pnas.1717139115.

12. Chunduru P, Phillips JJ, Molinaro AM. Prognostic Risk Stratification of Gliomas Using Deep Learning in Digital Pathology Images. *Neuro Oncol Adv*. Oxford University Press; 2022; doi: 10.1093/noajnl/vdac111.

13. Ertosun MG, Rubin DL. Automated Grading of Gliomas using Deep Learning in Digital Pathology Images: A modular approach with ensemble of convolutional neural networks. *AMIA Annu Symp Proc*. 2015:1899–9082015;

14. Ostrom QT, Price M, Neff C, Cioffi G, Waite KA, Kruchko C, et al.. CBTRUS Statistical Report: Primary Brain and Other Central Nervous System Tumors Diagnosed in the United States in 2015-2019. *Neuro Oncol*. 2022; doi: 10.1093/neuonc/noac202.

15. Weller M, van den Bent M, Preusser M, Le Rhun E, Tonn JC, Minniti G, et al.. EANO guidelines on the diagnosis and treatment of diffuse gliomas of adulthood. *Nat Rev Clin Oncol*. 2021; doi: 10.1038/s41571-020-00447-z.

16. Roux A, Roca P, Edjlali M, Sato K, Zanello M, Dezamis E, et al.. MRI Atlas of IDH Wild-Type Supratentorial Glioblastoma: Probabilistic Maps of Phenotype, Management, and Outcomes. *Radiology*. 2019; doi: 10.1148/radiol.2019190491.

17. Ellingson BM, Abrey LE, Nelson SJ, Kaufmann TJ, Garcia J, Chinot O, et al.. Validation of postoperative residual contrast-enhancing tumor volume as an independent prognostic factor for overall survival in newly diagnosed glioblastoma. *Neuro Oncol*. Neuro Oncol; 2018; doi: 10.1093/neuonc/noy053.

18. Puchalski RB, Shah N, Miller J, Dalley R, Nomura SR, Yoon J-G, et al.. An anatomic transcriptional atlas of human glioblastoma. *Science*. 2018; doi: 10.1126/science.aaf2666.

19. Klughammer J, Kiesel B, Roetzer T, Fortelny N, Nemc A, Nenning K-H, et al.. The DNA methylation landscape of glioblastoma disease progression shows extensive heterogeneity in time and space. *Nat Med*. 2018; doi: 10.1038/s41591-018-0156-x.

20. Neftel C, Laffy J, Filbin MG, Hara T, Shore ME, Rahme GJ, et al.. An Integrative Model of Cellular States, Plasticity, and Genetics for Glioblastoma. *Cell*. 2019; doi: 10.1016/j.cell.2019.06.024.

21. Wang Q, Hu B, Hu X, Kim H, Squatrito M, Scarpace L, et al.. Tumor Evolution of Glioma-Intrinsic Gene Expression Subtypes Associates with Immunological Changes in the Microenvironment. *Cancer Cell*. 2017; doi: 10.1016/j.ccell.2017.06.003.

22. Kaffes I, Szulzewsky F, Chen Z, Herting CJ, Gabanic B, Velázquez Vega JE, et al.. Human Mesenchymal glioblastomas are characterized by an increased immune cell presence compared to Proneural and Classical tumors. *Oncoimmunology*. 2019; doi: 10.1080/2162402X.2019.1655360.

23. Prabhu A, Kesarwani P, Kant S, Graham SF, Chinnaiyan P. Histologically defined intratumoral sequencing uncovers evolutionary cues into conserved molecular events driving gliomagenesis. *Neuro Oncol*. 2017; doi: 10.1093/neuonc/nox100.

24. Varn FS, Johnson KC, Martinek J, Huse JT, Nasrallah MP, Wesseling P, et al.. Glioma progression is shaped by genetic evolution and microenvironment interactions. *Cell*. 2022; doi: 10.1016/j.cell.2022.04.038.

25. Roetzer-Pejrimovsky T, Moser A-C, Atli B, Vogel CC, Mercea PA, Prihoda R, et al.. The Digital Brain Tumour Atlas, an open histopathology resource. *Sci Data*. 2022; doi: 10.1038/s41597-022-01157-0.

26. Roetzer T: WSI_histology. https://github.com/tovaroe/WSI_histology Accessed 2022 Apr 6.

27. Faryna K, van der Laak J, Litjens G. Tailoring automated data augmentation to H&E-stained histopathology. In: Heinrich M, Dou Q, de Bruijne M, Lellmann J, Schläfer A, Ernst F, editors. *Proceedings of the Fourth Conference on Medical Imaging with Deep Learning*. PMLR; p. 168–78.

28. Abadi M, Barham P, Chen J, Chen Z, Davis A, Dean J, et al.. TensorFlow: A System for Large-Scale Machine Learning. *12th USENIX symposium on operating systems design and implementation (OSDI 16)*. p. 265–83.

29. Chollet F. Xception: Deep Learning with Depthwise Separable Convolutions. *arXiv*. 2016; doi: 10.48550/arXiv.1610.02357.

30. Chollet FAO: Keras. https://keras.io (2015). Accessed 2022 Dec 21.

31. Waskom M. seaborn: statistical data visualization. *J Open Source Softw*. The Open Journal; 2021; doi: 10.21105/joss.03021.

32. Virtanen P, Gommers R, Oliphant TE, Haberland M, Reddy T, Cournapeau D, et al.. SciPy 1.0: fundamental algorithms for scientific computing in Python. *Nat Methods*. 2020; doi: 10.1038/s41592-019-0686-2.

33. Davidson-Pilon C. lifelines: survival analysis in Python. *J Open Source Softw*. The Open Journal; 2019; doi: 10.21105/joss.01317.

34. Pölsterl S. scikit-survival: A Library for Time-to-Event Analysis Built on Top of scikit-learn. *J Mach Learn Res*. 21:1–62020;

35. Hunter JD. Matplotlib: A 2D Graphics Environment. *Computing in Science Engineering*. 2007; doi: 10.1109/MCSE.2007.55.

36. Pedregosa F, Varoquaux G, Gramfort A, Michel V, Thirion B, Grisel O, et al.. Scikit-learn: Machine Learning in Python. *J Mach Learn Res*. 12:2825–302011;

37. Bankhead P, Loughrey MB, Fernández JA, Dombrowski Y, McArt DG, Dunne PD, et al.. QuPath: Open source software for digital pathology image analysis. *Sci Rep*. 2017; doi: 10.1038/s41598-017-17204-5.

38. Brennan CW, Verhaak RGW, McKenna A, Campos B, Noushmehr H, Salama SR, et al.. The somatic genomic landscape of glioblastoma. *Cell*. 2013; doi: 10.1016/j.cell.2013.09.034.

39. : cBioPortal for Cancer Genomics. https://www.cbioportal.org/ Accessed 2023 Aug 3.

40. : GDC. https://portal.gdc.cancer.gov/ Accessed 2023 Aug 3.

41. Bhowal P, Sen S, Velasquez JD, Sarkar R. Fuzzy ensemble of deep learning models using choquet fuzzy integral, coalition game and information theory for breast cancer histology classification. *Expert Syst Appl*. 2022; doi: 10.1016/j.eswa.2021.116167.

42. Xue D, Zhou X, Li C, Yao Y, Rahaman MM, Zhang J, et al.. An Application of Transfer Learning and Ensemble Learning Techniques for Cervical Histopathology Image Classification. *IEEE Access*. 2020; doi: 10.1109/ACCESS.2020.2999816.

43. Shaban M, Awan R, Fraz MM, Azam A, Tsang Y-W, Snead D, et al.. Context-Aware Convolutional Neural Network for Grading of Colorectal Cancer Histology Images. *IEEE*

Trans Med Imaging. 2020; doi: 10.1109/TMI.2020.2971006.

44. Becker AP, Sells BE, Haque SJ, Chakravarti A. Tumor Heterogeneity in Glioblastomas: From Light Microscopy to Molecular Pathology. *Cancers* . 2021; doi: 10.3390/cancers13040761.

45. Engler JR, Robinson AE, Smirnov I, Hodgson JG, Berger MS, Gupta N, et al.. Increased microglia/macrophage gene expression in a subset of adult and pediatric astrocytomas. *PLoS One*. 2012; doi: 10.1371/journal.pone.0043339.

46. Rao A, Barkley D, França GS, Yanai I. Exploring tissue architecture using spatial transcriptomics. *Nature*. 2021; doi: 10.1038/s41586-021-03634-9.

47. Moses L, Pachter L. Museum of spatial transcriptomics. *Nat Methods*. 2022; doi: 10.1038/s41592-022-01409-2.

48. Roetzer-Pejrimovsky T: GBMatch_CNN: Predicting TS & risk from glioblastoma WSI. https://github.com/tovaroe/GBMatch_CNN Accessed 2023 Aug 24.

49. : GBMatch Supplementary Website. The DNA methylation landscape of glioblastoma disease progression shows extensive heterogeneity in time and space - Supplementary Website. https://www.medical-epigenomics.org/papers/GBMatch/ Accessed 2023 Aug 24.

50. Roetzer-Pejrimovsky T: GBMatch_CNN - additional data. https://zenodo.org/record/8358673. 2023; doi: 10.5281/zenodo.8358673

Manuscript including figures for smoother review
Click here to access/download;Manuscript;Manuscript incl figures.pdf

Deep learning links localized digital pathology phenotypes with transcriptional subtype and patient outcome in glioblastoma

Thomas Roetzer-Pejrimovsky[1,2], Karl-Heinz Nenning[3,4], Barbara Kiesel[5], Johanna Klughammer[6], Martin Rajchl[7], Bernhard Baumann[8], Georg Langs[4], Adelheid Woehrer[1,2]

1 Division of Neuropathology and Neurochemistry, Department of Neurology, Medical University of Vienna, Vienna, Austria.

2 Comprehensive Center for Clinical Neurosciences and Mental Health, Medical University of Vienna, Vienna, Austria

3 Center for Biomedical Imaging and Neuromodulation, Nathan Kline Institute, Orangeburg, NY, USA

4 Department of Biomedical Imaging and Image-Guided Therapy, Computational Imaging Research Lab, Medical University of Vienna, Vienna, Austria.

5 Department of Neurosurgery, Medical University of Vienna, Vienna, Austria.

6 Gene Center and Department of Biochemistry, Ludwig-Maximilians-Universität München, Munich, Germany.

7 Department of Computing and Medicine, Imperial College London, London, U.K.

8 Center for Medical Physics and Biomedical Engineering, Medical University of Vienna, Vienna, Austria.

Corresponding author:

Georg Langs: georg.langs@meduniwien.ac.at

Abstract

Background:

Deep-learning has revolutionized medical image analysis in cancer pathology, where it had a substantial clinical impact by supporting the diagnosis and prognostic rating of cancer. Among the first available digital resources in the field of brain cancer is glioblastoma, the most common and fatal brain cancer. At the histologic level, glioblastoma is characterized by abundant phenotypic variability that is poorly linked with patient prognosis. At the transcriptional level, three molecular subtypes are distinguished with mesenchymal-subtype tumors being associated with increased immune cell infiltration and worse outcome.

Results:

We address genotype-phenotype correlations by applying an Xception convolutional neural network to a discovery set of 276 digital H&E slides with molecular subtype annotation, and an independent TCGA-based validation cohort of 178 cases. Using this approach, we achieve high accuracy in H&E-based mapping of molecular subtypes (AUC for classical, mesenchymal, proneural = 0.84, 0.81, and 0.71, respectively; $p < 0.001$) and regions associated with worse outcome (univariable survival model $p < 0.001$, multivariable $p = 0.01$). The latter were characterized by higher tumor cell density ($p < 0.001$), phenotypic variability of tumor cells ($p < 0.001$), and decreased T-cell infiltration ($p = 0.017$).

Conclusions:

We introduce a novel CNN architecture for glioblastoma digital slides that accurately maps the spatial distribution of transcriptional subtypes and regions predictive of worse outcome, thereby showcasing the relevance of AI-enabled image mining in brain cancer.

Key words

Glioblastoma, deep learning, histology, digital pathology, risk score

Background

Computer vision has undergone a revolution in recent years, which was in large parts driven by the development of convolutional neural networks (CNNs) [1–3]. In digital pathology, major achievements included the precise segmentation of individual cells [4–7], histologic structures [8,9] and tumor tissues [10]. In glioma, so far, CNNs have been employed for tumor typing, grading, and prognostic rating [11–13]. Still, the links between histologic phenotypes and underlying genotypes remain insufficiently understood; a gap, which could be addressed using CNNs [2,3].

Glioblastoma is the most common and fatal brain tumor in adults [14]. Prognostic factors include patient age, clinical performance, tumor location and resectability, DNA methylation at the MGMT gene promoter, and receipt of multimodal treatment [15–17]. So far, no histology-based prognostic biomarker is available.

At the histologic level, glioblastoma is characterized by extensive within- and across-tumor variability ranging from small-celled to monstro-cellular and sarcomatous cells with recurrent formation of palisades around necroses and Scherer's secondary structures at the invasive front. Also, the composition of the microenvironment varies in space and time with bone marrow-derived macrophages being abundant in necrotic regions, brain-resident microglia within and surrounding tumor regions, as well as scattered lymphocytes in perivascular arrangements.

At the level of tumor biology, glioblastoma is characterized by complex genetic aberrations and transcriptional plasticity with considerable spatial and temporal variability (Figure S1) [18–20]. At the bulk-level, three transcriptional subtypes were defined, i.e., classical, mesenchymal and proneural, each being enriched for genetic alterations and microenvironmental factors [21]. Importantly, previous efforts to explore the spatial distribution of the transcriptional subtypes pointed towards associations between the proneural subtype and invasive edges with enhanced neuronal signaling, as well as the

mesenchymal subtype and perinecrotic areas with denser immune cell infiltration [22–24]. However, despite their biologic relevance, their translation into routine clinical assessments based on formalin-fixed paraffin-embedded (FFPE) tissues was largely prevented by the limited availability of FFPE-based spatial transcriptomics technology. Hence, a computational solution that enables their accurate prediction in spatial context based on ubiquitously available, cost-efficient H&E-stains would fuel their translation and clinical applicability.

Here, we introduce an end-to-end CNN that generates a histology-based risk score to estimate patient prognosis (RS-CNN) and maps the spatial distribution of transcriptional subtypes (TS-CNN, Fig. 1).

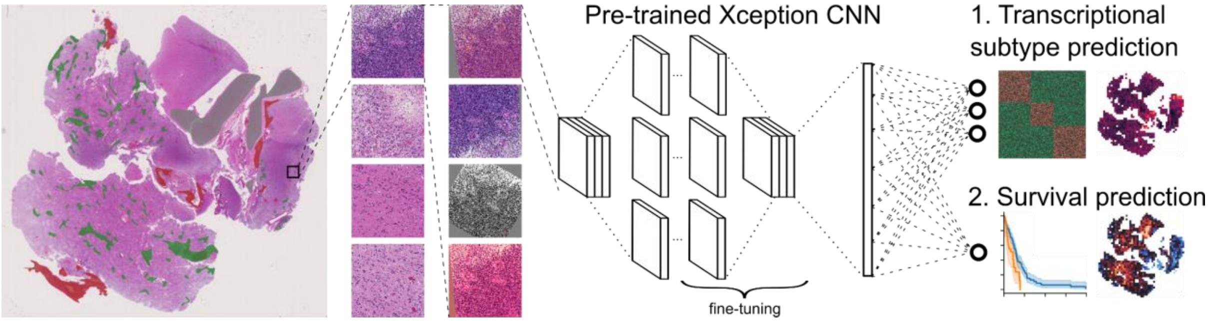

Fig. 1 Approach & network architecture. Digital slides were manually segmented (green: necrosis, red: bleeding, gray: pre-existing tissue) and smaller image tiles comprising mainly/only tumor tissue and infiltration zone were exported. For model training, extensive data augmentation was performed. The deep learning framework consisted of a pre-trained Xception CNN, in which the ultimate layer was replaced by a 3-neuron-layer (for transcriptional subtype prediction, *TS-CNN*) or a 1-neuron-layer (for survival prediction, *RS-CNN*). We used only histological image tiles as input to the model and no other information was introduced to either model.

Materials and methods

Patient cohort

We leveraged an existing longitudinal glioblastoma patient cohort comprising matched histological and DNA methylation-derived transcriptional subtypes at time of first and second surgery [19].

A total of 276 patients with digital histology and outcome data were included (table 1, *discovery cohort*) to train the *RS-CNN* using overall survival as a label. For 189 tumors, also transcriptional subtype information was available (table 1, *TS subcohort*), including the admixture of the different subtypes (summing up to 100%) which was used as ground truth for training [19]. Samples with at least 70% contribution by a given subtype were allocated to this subtype (e.g. classical-predominant, proneural-predominant, mesenchymal-predominant). Both the entire discovery cohort and the TS subcohort featured a similar age range and female-to-male ratio. However, the TS cohort was slightly biased towards an increased receipt of temozolomide-based radiochemotherapy and prolonged survival. We ultimately split each cohort into five equally large folds with comparable characteristics for internal 5-fold cross validation.

		**Discovery cohort**	**TS subcohort**
**Number of patients**		276	189
**Median Age [IQR]**		63.0 [53.8 - 70.5] years	62.0 [52.0 - 68.0] years
**F:m ratio**		0.62 (106:170)	0.64 (74:115)
**Combined radiochemotherapy (TMZ)**		205 (74.3 %)	153 (81.0 %)
**Median overall survival**		1.16 years	1.51 years
**Alive at last follow-up**		7 (2.54 %)	7 (3.7 %)
**TS**	**Classical predominant**	-	34 (17.99 %)

	**Mesenchymal predominant**	-	50 (26.46 %)
	**Proneural predominant**	-	21 (11.11 %)
	**Mixed**	-	84 (44.44 %)

Table 1. Demographics of the discovery cohort and the TS subcohort. The whole discovery cohort was used for risk score prediction. The TS subcohort was used for TS prediction. CNN: convolutional neural network, IQR: interquartile range, TMZ: temozolomide, TS: Transcriptional subtype

Handling of digital slides

H&E sections were digitized using a Hamamatsu NanoZoomer 2.0 HT slide scanner. On each digital slide, necrosis, preexisting brain parenchyma, bleeding, scar tissue and deformed tissue had been manually segmented by a board-certified neuropathologist (A.W.) using the ndp.view2-built-in annotation tool. The remaining areas were assigned to tumor areas. Each digital slide was converted to multiple (i.e. 6 to 2257) 1024x1024 pixel tiles at 20x magnification (456 px / μm) with 64px overlap using a custom MATLAB script (MATLAB R2017b, MathWorks) [25,26]. An accompanying spreadsheet contained the coordinates of each tile with the relative areas per segmented region. We defined perinecrotic regions as image tiles containing both tumor tissue and necrosis. Similarly, we defined the infiltration zone as tiles containing both tumor and preexisting tissue. For classifier training, only tiles with > 50% tumor tissue were kept. Patients with less than 50 different tiles had been excluded from further analysis. For training, we performed random cropping to 512x512 px and automated data augmentation with the H&E-specific algorithm of Faryna at runtime [27].

CNN architecture

We used TensorFlow 2.1.0 / keras for developing our deep learning pipeline [28]. As a base model, we used an Xception model [29] pre-trained on ImageNet available via the keras

model applications [30]. The input consisted of a (randomly sampled) WSI tile and no other information was introduced to the model. We froze all weights and added an extra layer depending on the target. For TS prediction, we added a fully connected 3-neuron layer with softmax activation. The TS target consisted of the three probabilities for each of the transcriptional subtypes. The mean squared error was backpropagated to update the weights. For risk score prediction, we added a single one-neuron layer with a linear activation function. The negative log likelihood was used as a loss function and was backpropagated to update the weights in a similar approach as Mobadersany et al. [11]. We adapted keras' DataFrameIterator such that for each new cycle through the digital slides, a new random image tile was selected per patient, randomly cropped and augmented. The TS-CNN and RS-CNN were trained independently of each other. Each model was first trained for 25 epochs with a custom 150 steps per epoch (for better performance) and a batch size of 64. We used the Adam optimizer with a learning rate of 0.001 and exponential learning rate decay every 400 steps at a decay rate of 0.9. For finetuning, the last 2 convolutional layers (4,741,632 of 20,861,480 parameters) of the Xception model were set trainable and the model was trained for 10 further epochs with 150 steps per epoch and a batch size of 64. Again, we used the Adam optimizer with a learning rate of 0.0001 and exponential learning rate decay every 400 steps at a decay rate of 0.8. During training, at the start of each fold 20 random batches were loaded into memory for validation. At the end of each epoch, the mean squared error (for TS prediction) or the c-index (for survival prediction) were calculated for the validation batches to keep track of the model performance.

We used 5-fold cross validation during model training. For the final validation, we let the trained models predict all validation tiles (with center crop to 512x512 px and no augmentation). The RS predictions were z-scored, the TS predictions were taken as they were, then all validation set predictions were concatenated into a single spreadsheet for further statistical analysis.

H&E mapping

To visualize the spatial distribution of the predicted targets directly in the digital slides, we performed the predictions on a set of windows covering the entire digital slide. We then mapped the predictions to the coordinates of those windows. Thereby, heatmaps were plotted in triplets representing the three transcriptional subtypes, or as a single map depicting the risk score [31].

Statistical analysis

Statistical analysis was conducted in Python 3.8.5. We performed permutation tests by label shuffling to compare our predicted risk scores to random guesses. To calculate p-values determining the significance of the RS and TS predictions, we performed label shuffling to generate a null distribution. Mann-Whitney-U and Wilcoxon tests were calculated with scipy [32]. Kaplan-Meier survival analysis and Cox proportional hazards models were performed using lifelines [33]. Harrel's c-index was calculated using sksurv [34]. Figures were drawn using matplotlib [35] and seaborn [31]. The confusion matrix and roc analysis were performed using sklearn [36]. To compare RS with TS scores, we assigned each tile to the subtype displaying the highest predicted score (winner-takes-all). Based on that annotation, we then calculated the mean risk score for each transcriptional subtype.

For UMAP plotting, we first concatenated the outputs of the penultimate CNN layers of all models obtaining 20,480 features for each image tile. We then used the umap package to plot UMAPs.

Characterization of the tumor microenvironment

We used QuPath 0.3.0 [37] for the following steps. To showcase the within-tumor histological variability, we used the inbuilt "density map" function. We first performed "fast

cell counts” on the H&E digital slides to obtain overall cellularity (i.e., cell density) and circularity (i.e., cell *roundness*). The tumor cell proliferation, tumor-associated macrophages (TAM) and lymphocytes (TIL) density maps were calculated from Ki-67-, CD68-, CD163-, HLA-DR- and CD8-stained digital slides using “positive cell detection”. The immunohistochemical stainings were performed on a Dako autostainer system with the following antibodies: CD3 (Thermo Scientific no. RM-9107-S1, 1:200), CD8 (Dako Cytomation no. M7103, 1:100), CD163 (Novocastra no. NCL-L-CD163, 1:1000), CD68 (Dako Cytomation no. M0814, 1:5000), HLA-DR (Dako Cytomation no. M0775, 1:400), Ki-67 (MIB-1) (Dako Cytomation no. M7240, 1:200), and CD34 (Novocastra no. NCL-l-END, 1:100). [19] To link TAM and TIL densities with transcriptional subtypes and risk tiles, we manually segmented the respective regions on neighboring digital slides (where available and adequate) (table S1). After using “positive cell detection”, we counted all stained cells in each region and divided this count by the respective area to obtain the number of stained cells per mm^2. For HLA-DR and CD34 we calculated the relative stained area in a similar fashion. Thus, we obtained a quantitative characterization of the tumor microenvironment per slide/patient. We calculated summary statistics on this slide/patient level to compare the different transcriptional subtype regions and high/low risk regions. The QuPath script with the specific parameters is provided in the appendix.

External validation using TCGA data

After successful internal validation, we re-trained our CNN models on our complete discovery dataset using the same parameters as previously stated. We then downloaded the clinical annotation for the TCGA glioblastoma cohort published by Brennan et al. [38] from cBioPortal [39]. We screened the GDC Data Portal for available diagnostic slides and downloaded them using the GDC Data Transfer Tool [40]. To match the inclusion criteria of our training cohort, we excluded slides of suboptimal quality (due to excessive artifacts, poor

staining, or non-FFPE H&E slides) and tumors with mutant or unknown IDH status. We manually segmented the tumor tissue and infiltration zone in concordance to the discovery cohort. We then applied the RS and TS CNNs to the validation set. We averaged the subtype predictions over all image tiles and let the highest subtype score determine the predicted subtype per sample. We considered samples with a mismatch between predicted subtype and TCGA bulk sequencing derived subtype as misclassified. Moreover, patients were assigned to two risk groups, depending on the fraction of *high risk* (z-score > 1) tiles (cut-off 25%). High-risk samples of patients who survived > 18 months and low-risk samples of patients with < 12 months survival were considered misclassified.

Analyses

H&E-based mapping of transcriptional subtypes

The accuracy for predicting the predominant subtype was 66.7 % as compared to a random guess accuracy of 38.67 % [+/- 0.4 %] ($p < 0.001$, permutation test, Figure 2a, b). The mean squared error was 0.08 in the validation folds as compared to 0.11 [+/- 0.003] for random predictions ($p < 0.001$, permutation test). Overall, the spatial distribution of subtypes aligned well with the segmented tumor regions (Figure 2c and 2d) both upon visual inspection of the heatmaps as well as upon quantification at the cohort-level. Precisely, median predictive scores were significantly higher for proneural in the infiltration zone ($p < 0.001$, MWU, Figure 2e), and for mesenchymal in perinecrotic areas ($p = 0.021$, MWU Figure 2f). Likewise, a significantly higher cellularity and tendency to larger fractions of cycling cells were found in classical areas ($p < 0.001$, Wilcoxon test, Figure 2g & $p < 0.05$, MWU). At the individual cell level, nuclear circularity was highest in proneural and lowest in mesenchymal areas (all $p < 0.001$, Wilcoxon, Figure 2h). Ultimately, we found increased infiltration by CD68+, CD163+ and HLA-DR+ myeloid cells and CD3+, CD8+ TILs in mesenchymal regions (all $p < 0.006$,

MWU, Figure 2i). Likewise, areas covered by CD34+ vessels were enriched in mesenchymal as compared to proneural ($p < 0.01$, MWU) or classical ($p = 0.02$, MWU) regions.

	**Classical**	**Mesenchymal**	**Proneural**	**p-value**
**Cellularity (per mm²)**	6146<br>[4800 - 7574]	5484<br>[4046 - 6353]	5321<br>[3897 - 6833]	p < 0.001
**Circularity**	0.79<br>[0.78 - 0.81]	0.78<br>[0.77 - 0.8]	0.8<br>[0.78 - 0.82]	p < 0.001
**CD163⁺ cells (per mm²)**	9<br>[1 - 59]	348<br>[101 - 871]	37<br>[8 - 91]	p = 0.027
**CD3⁺ cells (per mm²)**	34<br>[17 - 82]	129<br>[53 - 310]	25<br>[15 - 52]	p = 0.006
**CD68⁺ cells (per mm²)**	96<br>[23 - 277]	243<br>[98 - 573]	72<br>[24 - 194]	p < 0.001
**CD8⁺ cells (per mm²)**	10<br>[5 - 20]	36<br>[19 - 81]	9<br>[4 - 18]	p < 0.001
**MIB⁺ cells (per mm²)**	290<br>[128 - 630]	108<br>[59 - 214]	60<br>[26 - 581]	p < 0.001
**CD34**	4 %<br>[3 - 6]	5 %<br>[4 - 11]	2 %<br>[1 - 4]	p < 0.001
**HLA-DR**	2 %<br>[0 - 9]	8 %<br>[4 - 18]	1 %<br>[0 - 3]	p < 0.001

Table 2. Comparison of cellular phenotype and immunohistochemical parameters [median, IQR] between different predicted TS. Given values represent a summary statistic over all slides and the whole respective subtype region (if present on their digital slide) was evaluated for each patient. The p-values were calculated using the Kruskal-Wallis H-test.

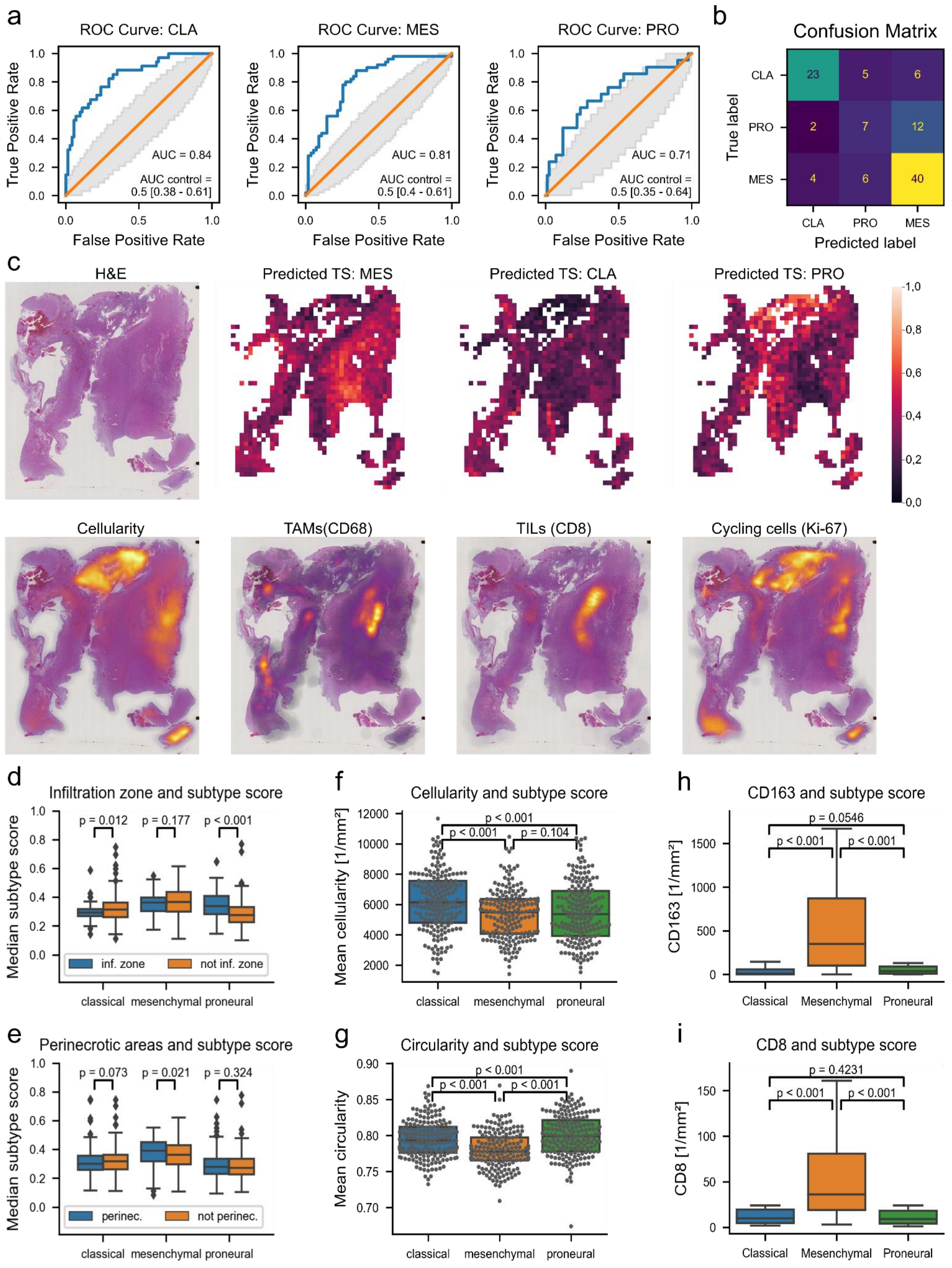

Fig. 2 Prediction of the transcriptional subtypes (TS). (a) Receiver operating characteristic (ROC) curves and area-under-the-curve per TS (b) confusion matrix for the prediction of TS in samples with a predominant TS. (c) One representative sample with three heat-maps depicting TS scores at the whole slide-level and density maps for cell density, CD68+ TAMs, CD8+ TILs, and Ki67+ cycling cells

(d)-(i) Correlative analyses between different histological aspects and TS scores. Of note, single outliers in panels (i) and (j) are not shown for better illustration.

H&E-based risk score prediction

The risk score prediction model (*RS-CNN*) was trained end-to-end on histological images alone using the Cox loss function (negative log-likelihood), which yielded a single risk score as output. To obtain patient-level predictions, the predicted scores per tile were normalized (z-scored) across the entire cohort and aggregated using the arithmetic mean. Additionally, the fraction of high-risk tiles (z-scored risk > 1) was calculated per digital slide and their distribution plotted as a heatmap (Fig. 3a). In the validation folds, the risk scores were strongly associated with survival upon univariable ($p < 0.001$, Fig. 3b) and multivariable analyses ($p = 0.013$, table 3).

	**HR**	**p-value**
**Age**	1.025 (1.015 - 1.036)	< 0.001
**Male sex**	1.14 (0.88 - 1.48)	0.331
**Radiochemotherapy (TMZ)**	0.43 (0.32 - 0.58)	< 0.001
**RS CNN**	1.32 (1.06 - 1.65)	0.013

Table 3. Cox multivariable survival model. HR for age is calculated for each 1-year increase of patient age.

The median risk score was significantly lower in infiltration zones (Fig. 3e, $p = 0.009$, MWU) and not enhanced in perinecrotic areas ($p=0.446$, MWU). High-risk areas were characterized by higher cellularity ($p < 0.001$, Wilcoxon), decreased nuclear circularity (reflecting polymorphous nuclei, $p < 0.001$, Wilcoxon), fewer CD8+ cells ($p = 0.017$, MWU), and a trend towards fewer CD3+ cells ($p = 0.06$, MWU). There was no significant difference in CD68+,

CD163+ or HLA-DR+ myeloid cell density (p = 0.13, 0.435, and 0.25, respectively, MWU), the fraction of cycling cells (p = 0.19, MWU), and microvessel density (p = 0.31, MWU).

	High risk	Low risk	p-value
**Cellularity (per mm²)**	5877 [4336 - 7302]	5524 [3885 - 6891]	$p < 0.001$
**Circularity**	0.78 [0.75 - 0.8]	0.79 [0.77 - 0.81]	$p < 0.001$
**CD163$^+$ cells (per mm²)**	46 [8 - 234]	27 [7 - 366]	$p = 0.35$
**CD3$^+$ cells (per mm²)**	33 [16 - 70]	38 [22 - 221]	$p = 0.063$
**CD68$^+$ cells (per mm²)**	108 [26 - 219]	157 [45 - 274]	$p = 0.127$
**CD8$^+$ cells (per mm²)**	9 [3 - 23]	16 [8 - 58]	$p = 0.017$
**MIB$^+$ cells (per mm²)**	248 [45 - 634]	138 [48 - 340]	$p = 0.191$
**CD34**	3 [2 - 5] %	4 [2 - 6] %	$p = 0.306$
**HLA-DR**	3 [0 - 9] %	1 [0 - 9] %	$p = 0.25$

Table 4. Comparative analysis between high- and low-risk regions across histological and immunohistochemical parameters [median, IQR]. P-values were calculated using the Wilcoxon signed-rank test (Cellularity, Circularity) and the Mann-Whitney U test (immunohistochemical stainings), respectively.

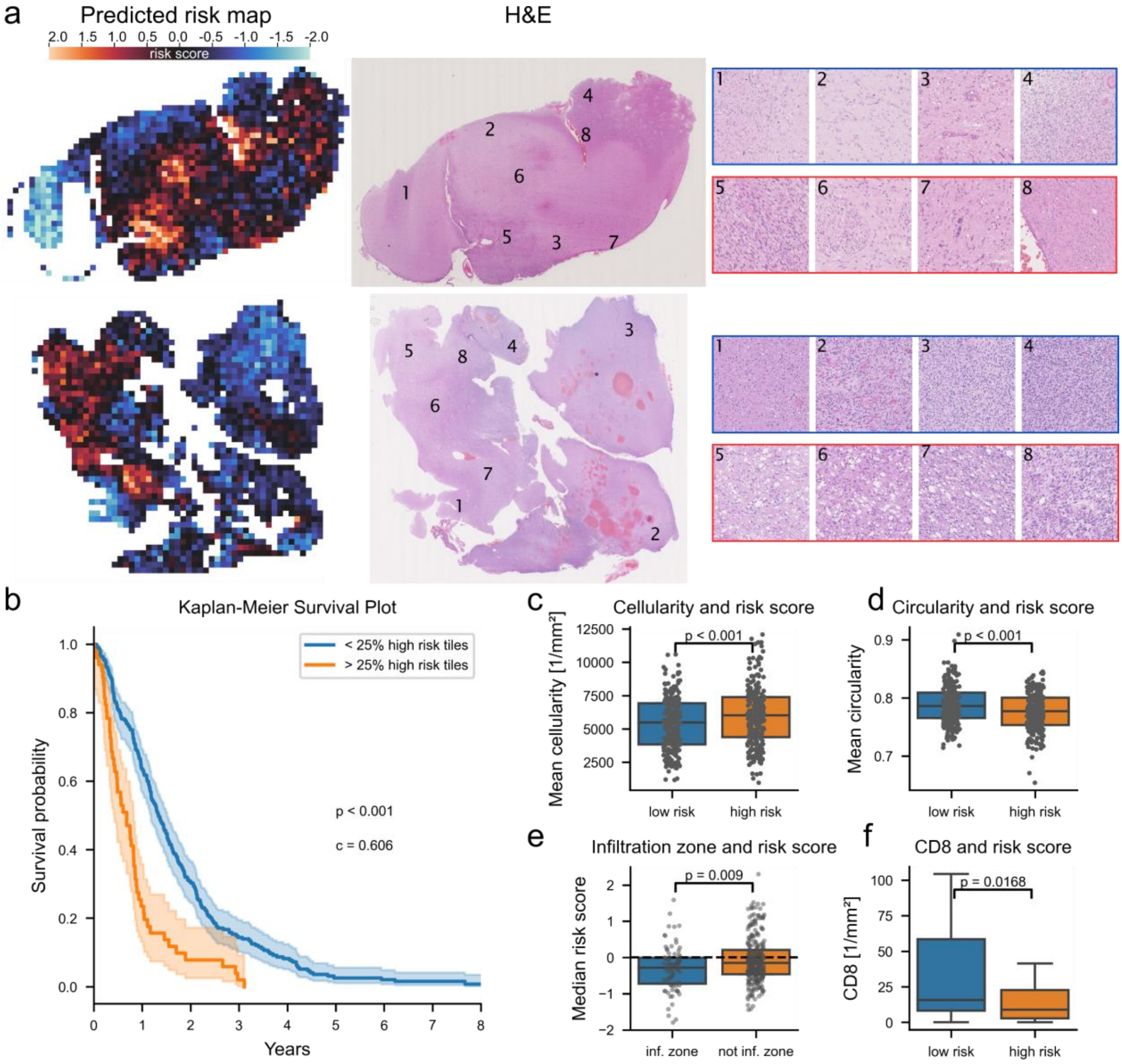

Fig. 3 Prediction of the risk score. (a) Risk maps and corresponding H&E slides of two representative cases. Higher risk scores are depicted in a red hue and lower risk scores in a blue hue. Higher-magnification images are given for the numbered regions of the WSI (top row: low-risk regions, bottom row: high-risk regions). (b) Kaplan-Meier plot stratified at 25% high-risk tiles ($p < 0.001$, log rank test, Harrel's c-index = 0.6) (c)-(f) Box plots depicting associations between risk scores and selected histological and immunohistochemical aspects.

Integration of risk scores with transcriptional subtypes

Ultimately, we aimed to link predicted risk scores with TS scores. Dimensionality reduction of aggregated TS and RS features resulted in one continuous feature space with smaller peripheral clusters that mostly represented individual patients. Still, also regional clusters relating to gross histologic features such as cellularity or nuclear circularity emerged (Fig 4a&b).

Furthermore, we calculated the mean predicted risk score for each of the transcriptional subtypes per slide, which resulted in signficantly higher risk scores in classical and mesenchymal than in proneural areas (Fig 4c, p = 0.001 and 0.02, respectively, Wilcoxon).

External validation in TCGA datasets

Finally, we sought to validate the performance of our models in an independent TCGA dataset (Fig 5a). Applying the previously defined cut-off of 25% high risk tiles, resulted in a statistically significant separation of survival curves (p = 0.003, logrank test, Figure 5b). Of note, 14% of the validation set were assigned to the high-risk group, as compared to 18% in the discovery cohort. Harrel's c-index was 0.52 and the mean risk score was not significantly associated with survival (Cox regression univariable HR = 1.4 +/- 0.25, p = 0.16; multivariable HR = 1.2 +/- 0.18, p = 0.31). In parallel, the accuracy for predicting the transcriptional subtypes was 56.2% compared to a random guess accuracy of 34.3% [+/- 0.4 %] in the validation set (p < 0.001, permutation test, Figure 5c & d). Interestingly, the accuracy was highest for predicting the mesenchymal subtype (AUC = 0.746) as compared to the classical (AUC = 0.704) and proneural (AUC = 0.697) subtypes.

To better understand the potential drawbacks and pitfalls of the trained CNNs, we specifically looked at misclassified samples (Figure 6). Overall, out of 178 total samples, 78 displayed misclassified transcriptional subtypes, 3 were misclassified as high-risk and 57 were misclassified and low-risk.For 28 samples, both the transcriptional subtype and survival

were misclassified. We found that many (29.5%) subtype misclassifications were “near correct”, i.e., the difference between the true subtype score and the predicted subtype score was < 0.01. Upon qualitative assessment of the misclassified cases, we further found that many samples had relatively little tumor tissue.

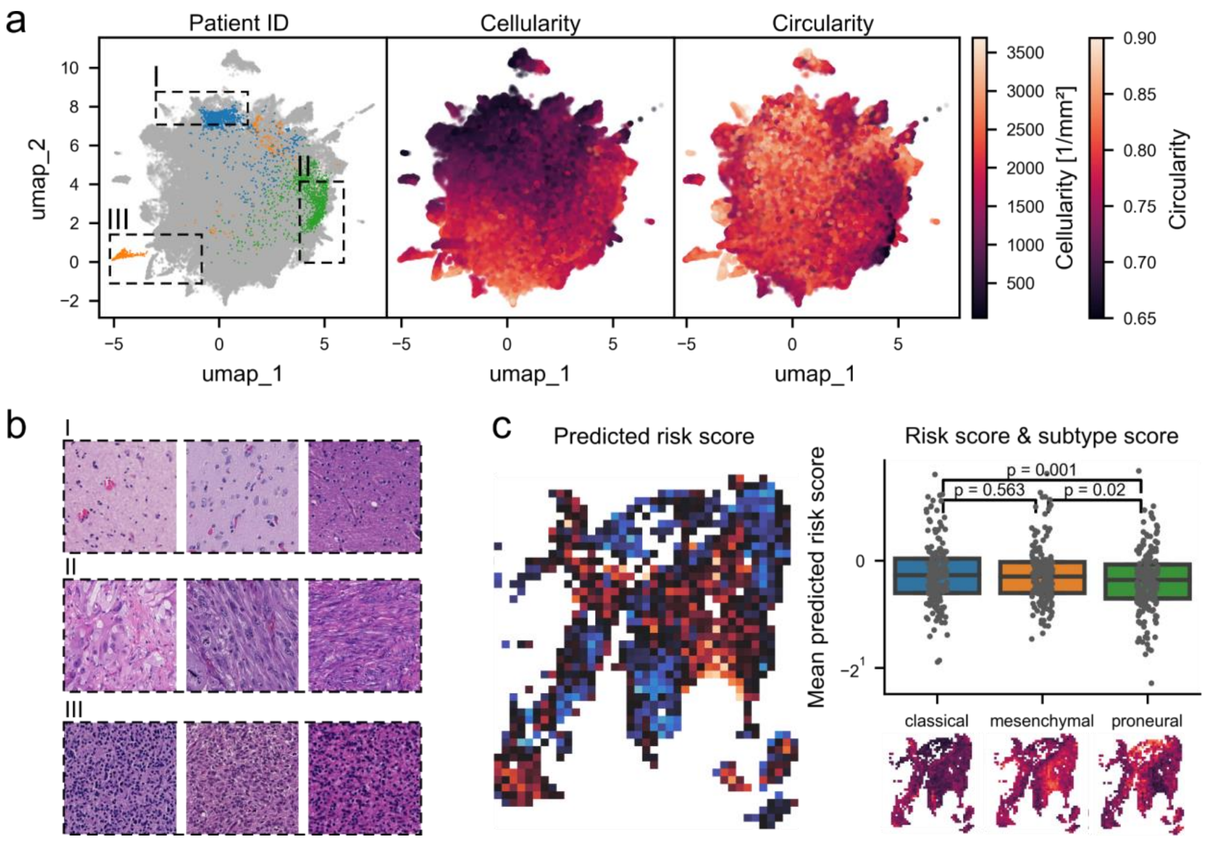

Fig. 4 Feature landscape and integration of risk and transcriptional subtype scores. (a) UMAP projection of all features with highlighted regions I-III. In the leftmost UMAP, image tiles from three exemplary patients are highlighted in blue, green and orange, respectively and all other patients are plotted in gray. (b) Representative histological images corresponding to regions I-III with (I) corresponding to infiltration zone, (II) monstro-sarcomatoid phenotypes and (III) round-cell & cell-dense regions. (c) Correlation between risk and transcriptional subtype prediction.

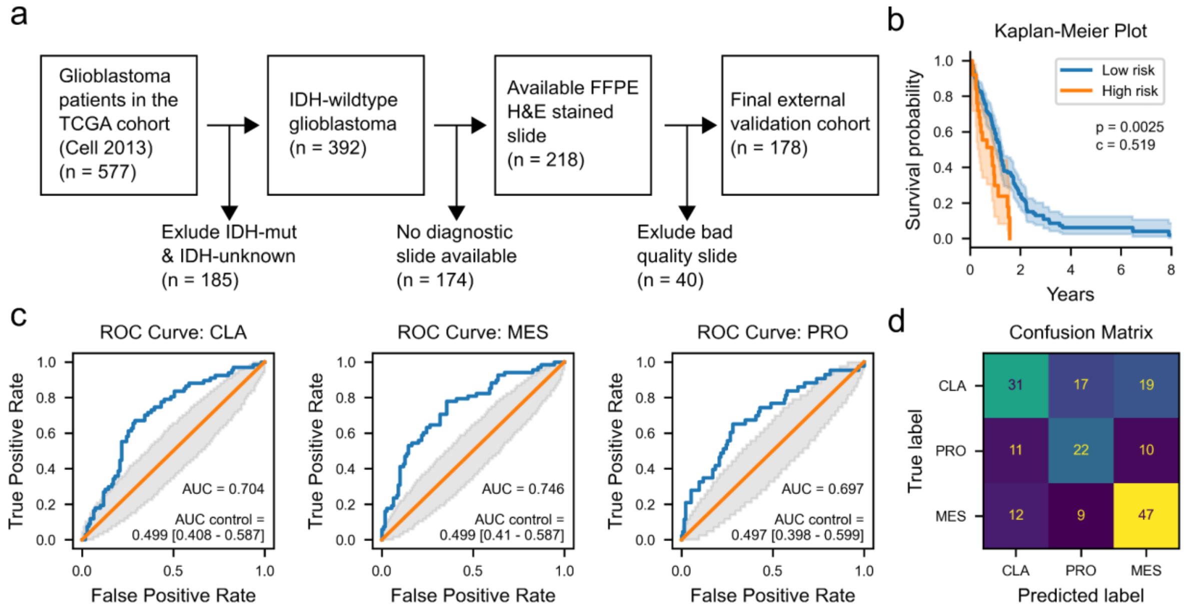

Fig. 5 External validation in TCGA data [38]. (a) Inclusion flowchart to match inclusion criteria for the training cohort. (b) Kaplan-Meier plot stratified at content of 25% high-risk tiles (p = 0.003, logrank test), (c) ROC and AUC values per TS, (d) confusion matrix with TS prediction accuracy.

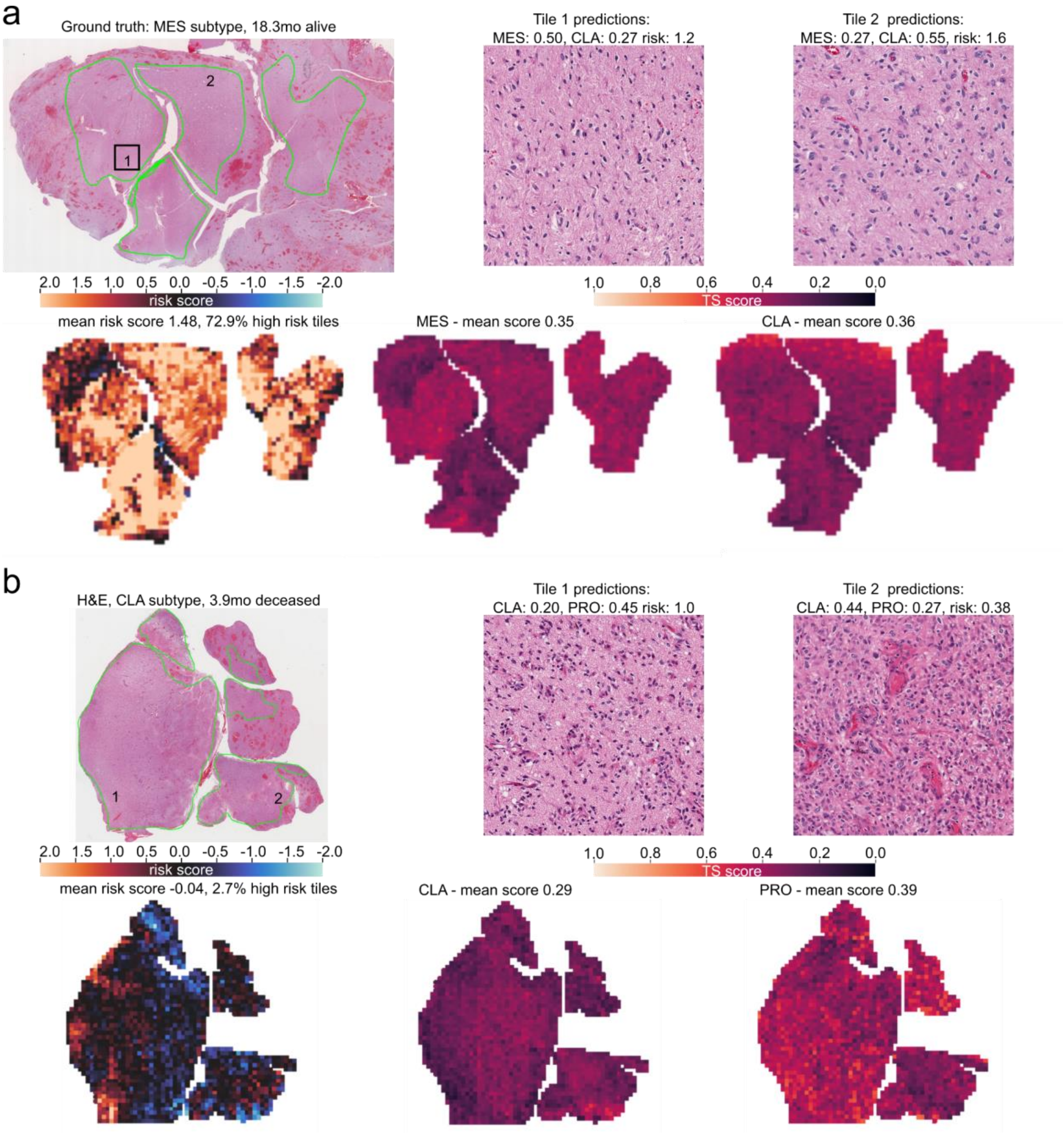

Fig. 6 Misclassified TCGA samples. (a) Patient with > 18 months survival and mesenchymal subtype, that was misclassified as high risk and classical subtype. (b) Patient with 3.9 months survival and classical subtype that was misclassified as proneural subtype.

Green segmentations on the H&E correspond to the manually segmented tumor tissue, upon which the CNN prediction was based.

Discussion

In the present study, we leverage deep learning on digital glioblastoma slides to address two relevant applications: 1. the mining of subvisual histological patterns for prognostic information, and 2. the prediction of molecular information using the transcriptional subtypes as a showcase.

A major strength of our approach is the sample size of the discovery cohort, which is the largest publicly available digital resource for FFPE digital slides in glioblastoma reported to date [19,38]. This resource comprises 460GB corresponding to 220,000 individual tiles including 146,000 tumor tiles. Previous works had already demonstrated the applicability of CNNs for classification and grading of gliomas [11–13]. We here used a pre-trained Xception CNN model, which is relatively lightweight compared to other CNN architectures while performing on par or better on the ImageNet classification task [29,41–43].

Our first and foremost result is the identification of a novel histology-based prognostic factor. Even though glioblastoma is known for its extensive inter- and intratumoral heterogeneity at the histological level as reflected by the term “multiforme” in previous classifications, no histology-based marker had been consistently linked to outcome. Hence, it is exciting to see that the RS-CNN was able to capture clinically meaningful prognostic information in the format of a risk score that can be used to stratify patients into risk groups. At the same time, however, spatial mapping of the risk score allows interpretability in local micro- and global macroenvironmental context. In our case, high-risk regions were characterized by a simultaneous increase in tumor cell density and decrease in TIL surveillance, both parameters that vary considerably across glioblastoma whole slides and are not easily captured by visual inspections of H&E slides alone [44].

Regarding our second task, the prediction and spatial mapping of transcriptional subtypes, reassuringly, our results grossly support established associations between molecular subtype regions and microenvironmental aspects such as necrosis and TAM infiltration and the mesenchymal subtype [21,23,45]. Extending beyond previous work, we demonstrate

that also the nuclear morphology and density of the tumor cells differ across subtype-specific regions. Intuitively, cells residing in proneural areas were linked to higher nuclear circularity potentially reflecting uniform “oligodendroglial or OPC-like” tumor cell shapes and/or admixture of non-neoplastic cells. Likewise, we observed lower cell density in mesenchymal regions that could relate to the presence of necrotic areas or in case of proneural regions to paucicellular infiltration zones. Directly linking histological patterns to these cellular states will be an important next step that requires single-cell transcriptomic data [20].

When ultimately connecting transcriptional subtypes with risk, high risk regions were only marginally enriched for mesenchymal and classical regions, which is somewhat surprising given that only the mesenchymal subtype had been previously linked with adverse outcome but did not seem to contribute major information to the RS CNN model [21]. Importantly, however, our TS CNN was able to predict the presence and distribution of subtypes solely based on H&E slides, which are ubiquitously available as part of any routine diagnostic assessment (also in smaller labs without established molecular workflows), highly cost-efficient, and save weeks as compared with technically demanding spatially-resolved RNA-sequencing [46,47].

We thoroughly validated both the RS-CNN and TS-CNN in an external cohort using unseen digital slides derived from TCGA [38], which resulted in a slightly lower accuracy in the validation set, which was to be expected for two reasons. First, the datasets differed in their molecular annotation as for TCGA slides only the predominant subtype information was available as compared to the subtype-specific probabilities we had for the discovery cohort. Second, in the TCGA cohort, bulk RNA-sequencing and digital slides were likely derived from different regions of the same tumor.

Our study has limitations. First, for internal validation we performed 5-fold cross validation instead of using an additional internal test set, which was mostly due to the sample size. Second, even though the high risk and low risk groups showed significantly different survival in the external validation cohort, the underlying numerical risk score failed to accurately capture these survival differences upon univariable analysis. Third, the molecular annotation

for both cohorts was obtained from bulk sequencing, and it will be important to follow up on our models using datasets that comprise matched H&E slides and spatially-resolved sequencing data at single cell resolution.

Conclusions

In sum, we present two deep learning-based convolutional neural networks that complement the histologic assessment of glioblastoma by adding spatially-resolved information on transcriptional subtype and prognostic patient information. The code can be easily adapted to similar problems and is provided under a permissive license.

Availability of Source Code and Requirements

The code for CNN training is available via github [48]. This includes code for the initial training of CV-folds and corresponding exemplary histological data and clinical annotation. Moreover, we provide a final fully trained predictor as *gbm_predictor.py* that has been trained with the complete discovery dataset and may be used for assessing new digital slides (supported formats are ndpi and svs). Additionally, we also provide QuPath groovy-scripts for the analysis of the tumor microenvironment.

- Project name: GBMatch_CNN
- Project home page: https://github.com/tovaroe/GBMatch_CNN
- Operating system(s): Platform independent
- Programming language: Python, Groovy (QuPath)
- Other requirements: Python 3.6 or higher, additional dependencies are listed on the project home page; QuPath >= 0.3.0

- License: GPL-3.0

Data Availability

The complete slide scan library, including H&E stained slides and corresponding tissue segmentations as well as immunohistochemically stained slides, is available online via the GBMatch supplementary website [19,49]. All pre-selected image tiles used for training and segmentations for the immunohistochemically stained slides are available via an accompanying zenodo repository [50]. The external TCGA validation dataset is available via cBioPortal [39] and the GDC Data Portal [40].

Declarations

List of abbreviations

CNN: Convolutional neural network

FFPE: Formalin-fixed paraffin-embedded

RS-CNN: Risk score CNN

TAM: Tumor-associated macrophages

TCGA: The Cancer Genome Atlas

TIL: Tumor-infiltrating lymphocytes

TS: Transcriptional subtype

TS-CNN: Transcriptional subtype CNN

Ethics approval and consent to participate

The present study has been approved by the Ethics Committee of the Medical University of Vienna (EK1691-2017) and complies with all relevant ethical, legal and institutional regulations.

Competing interests

GL is chief scientist at contextflow GmbH. The other authors declare no competing interests.

Funding

This work was supported by the Austrian Science Fund projects KLI394 and TAI98B to AW. Thomas Roetzer-Pejrimovsky is a recipient of a DOC Fellowship (25262) of the Austrian Academy of Sciences at the Division of Neuropathology and Neurochemistry, Department of Neurology, Medical University of Vienna. Parts of the computational work and digital resources were supported by the Vienna Science and Technology Fund (WWTF) Project No. LS20-034 to AW and Project No. LS20-065 to GL.

Authors' contributions

Conceptualization: TRP, MR, BB, GL, AW; Methodology: TRP, KHN, MR, BB, GL, AW; Formal analysis and investigation: TRP; Writing - original draft preparation: TRP, AW; Writing - review and editing: all authors; Funding acquisition: TRP, GL, AW; Resources: TRP, BK, JK, AW; Supervision: BB, GL, AW.

Acknowledgements

We thank Christoph Bock for data support. We thank NVIDIA for the donation of a TITAN Xp GPU.

References

1. LeCun Y, Bengio Y, Hinton G. Deep learning. *Nature*. 2015; doi: 10.1038/nature14539.

2. Jiang Y, Yang M, Wang S, Li X, Sun Y. Emerging role of deep learning-based artificial intelligence in tumor pathology. *Cancer Commun*. 2020; doi: 10.1002/cac2.12012.

3. Chen RJ, Lu MY, Williamson DFK, Chen TY, Lipkova J, Noor Z, et al.. Pan-cancer integrative histology-genomic analysis via multimodal deep learning. *Cancer Cell*. 2022; doi: 10.1016/j.ccell.2022.07.004.

4. Lal S, Das D, Alabhya K, Kanfade A, Kumar A, Kini J. NucleiSegNet: Robust deep learning architecture for the nuclei segmentation of liver cancer histopathology images. *Comput Biol Med*. 2021; doi: 10.1016/j.compbiomed.2020.104075.

5. Falk T, Mai D, Bensch R, Çiçek Ö, Abdulkadir A, Marrakchi Y, et al.. U-Net: deep learning for cell counting, detection, and morphometry. *Nat Methods*. 2019; doi: 10.1038/s41592-018-0261-2.

6. Sirinukunwattana K, Ahmed Raza SE, Yee-Wah Tsang, Snead DRJ, Cree IA, Rajpoot NM. Locality Sensitive Deep Learning for Detection and Classification of Nuclei in Routine Colon Cancer Histology Images. *IEEE Trans Med Imaging*. 2016; doi: 10.1109/TMI.2016.2525803.

7. Naylor P, Lae M, Reyal F, Walter T. Segmentation of Nuclei in Histopathology Images by Deep Regression of the Distance Map. *IEEE Trans Med Imaging*. 2019; doi: 10.1109/TMI.2018.2865709.

8. Hermsen M, de Bel T, den Boer M, Steenbergen EJ, Kers J, Florquin S, et al.. Deep Learning-Based Histopathologic Assessment of Kidney Tissue. *J Am Soc Nephrol*. 2019; doi: 10.1681/ASN.2019020144.

9. Graham S, Chen H, Gamper J, Dou Q, Heng P-A, Snead D, et al.. MILD-Net: Minimal information loss dilated network for gland instance segmentation in colon histology images. *Med Image Anal*. 2019; doi: 10.1016/j.media.2018.12.001.

10. Ehteshami Bejnordi B, Veta M, Johannes van Diest P, van Ginneken B, Karssemeijer N, Litjens G, et al.. Diagnostic Assessment of Deep Learning Algorithms for Detection of Lymph Node Metastases in Women With Breast Cancer. *JAMA*. 2017; doi: 10.1001/jama.2017.14585.

11. Mobadersany P, Yousefi S, Amgad M, Gutman DA, Barnholtz-Sloan JS, Velázquez Vega JE, et al.. Predicting cancer outcomes from histology and genomics using convolutional networks. *Proc Natl Acad Sci U S A*. 2018; doi: 10.1073/pnas.1717139115.

12. Chunduru P, Phillips JJ, Molinaro AM. Prognostic Risk Stratification of Gliomas Using Deep Learning in Digital Pathology Images. *Neuro Oncol Adv*. Oxford University Press; 2022; doi: 10.1093/noajnl/vdac111.

13. Ertosun MG, Rubin DL. Automated Grading of Gliomas using Deep Learning in Digital Pathology Images: A modular approach with ensemble of convolutional neural networks. *AMIA Annu Symp Proc*. 2015:1899–9082015;

14. Ostrom QT, Price M, Neff C, Cioffi G, Waite KA, Kruchko C, et al.. CBTRUS Statistical Report: Primary Brain and Other Central Nervous System Tumors Diagnosed in the United States in 2015-2019. *Neuro Oncol*. 2022; doi: 10.1093/neuonc/noac202.

15. Weller M, van den Bent M, Preusser M, Le Rhun E, Tonn JC, Minniti G, et al.. EANO guidelines on the diagnosis and treatment of diffuse gliomas of adulthood. *Nat Rev Clin Oncol*. 2021; doi: 10.1038/s41571-020-00447-z.

16. Roux A, Roca P, Edjlali M, Sato K, Zanello M, Dezamis E, et al.. MRI Atlas of IDH Wild-Type Supratentorial Glioblastoma: Probabilistic Maps of Phenotype, Management, and Outcomes. *Radiology*. 2019; doi: 10.1148/radiol.2019190491.

17. Ellingson BM, Abrey LE, Nelson SJ, Kaufmann TJ, Garcia J, Chinot O, et al.. Validation of postoperative residual contrast-enhancing tumor volume as an independent prognostic factor for overall survival in newly diagnosed glioblastoma. *Neuro Oncol*. Neuro Oncol; 2018; doi: 10.1093/neuonc/noy053.

18. Puchalski RB, Shah N, Miller J, Dalley R, Nomura SR, Yoon J-G, et al.. An anatomic transcriptional atlas of human glioblastoma. *Science*. 2018; doi: 10.1126/science.aaf2666.

19. Klughammer J, Kiesel B, Roetzer T, Fortelny N, Nemc A, Nenning K-H, et al.. The DNA methylation landscape of glioblastoma disease progression shows extensive heterogeneity in time and space. *Nat Med*. 2018; doi: 10.1038/s41591-018-0156-x.

20. Neftel C, Laffy J, Filbin MG, Hara T, Shore ME, Rahme GJ, et al.. An Integrative Model of Cellular States, Plasticity, and Genetics for Glioblastoma. *Cell*. 2019; doi: 10.1016/j.cell.2019.06.024.

21. Wang Q, Hu B, Hu X, Kim H, Squatrito M, Scarpace L, et al.. Tumor Evolution of Glioma-Intrinsic Gene Expression Subtypes Associates with Immunological Changes in the Microenvironment. *Cancer Cell*. 2017; doi: 10.1016/j.ccell.2017.06.003.

22. Kaffes I, Szulzewsky F, Chen Z, Herting CJ, Gabanic B, Velázquez Vega JE, et al.. Human Mesenchymal glioblastomas are characterized by an increased immune cell presence compared to Proneural and Classical tumors. *Oncoimmunology*. 2019; doi: 10.1080/2162402X.2019.1655360.

23. Prabhu A, Kesarwani P, Kant S, Graham SF, Chinnaiyan P. Histologically defined intratumoral sequencing uncovers evolutionary cues into conserved molecular events driving gliomagenesis. *Neuro Oncol*. 2017; doi: 10.1093/neuonc/nox100.

24. Varn FS, Johnson KC, Martinek J, Huse JT, Nasrallah MP, Wesseling P, et al.. Glioma progression is shaped by genetic evolution and microenvironment interactions. *Cell*. 2022; doi: 10.1016/j.cell.2022.04.038.

25. Roetzer-Pejrimovsky T, Moser A-C, Atli B, Vogel CC, Mercea PA, Prihoda R, et al.. The Digital Brain Tumour Atlas, an open histopathology resource. *Sci Data*. 2022; doi: 10.1038/s41597-022-01157-0.

26. Roetzer T: WSI_histology. https://github.com/tovaroe/WSI_histology Accessed 2022 Apr 6.

27. Faryna K, van der Laak J, Litjens G. Tailoring automated data augmentation to H&E-stained histopathology. In: Heinrich M, Dou Q, de Bruijne M, Lellmann J, Schläfer A, Ernst F, editors. *Proceedings of the Fourth Conference on Medical Imaging with Deep Learning*. PMLR; p. 168–78.

28. Abadi M, Barham P, Chen J, Chen Z, Davis A, Dean J, et al.. TensorFlow: A System for Large-Scale Machine Learning. *12th USENIX symposium on operating systems design and implementation (OSDI 16)*. p. 265–83.

29. Chollet F. Xception: Deep Learning with Depthwise Separable Convolutions. *arXiv*. 2016; doi: 10.48550/arXiv.1610.02357.

30. Chollet FAO: Keras. https://keras.io (2015). Accessed 2022 Dec 21.

31. Waskom M. seaborn: statistical data visualization. *J Open Source Softw*. The Open Journal; 2021; doi: 10.21105/joss.03021.

32. Virtanen P, Gommers R, Oliphant TE, Haberland M, Reddy T, Cournapeau D, et al.. SciPy 1.0: fundamental algorithms for scientific computing in Python. *Nat Methods*. 2020; doi: 10.1038/s41592-019-0686-2.

33. Davidson-Pilon C. lifelines: survival analysis in Python. *J Open Source Softw*. The Open Journal; 2019; doi: 10.21105/joss.01317.

34. Pölsterl S. scikit-survival: A Library for Time-to-Event Analysis Built on Top of scikit-learn. *J Mach Learn Res*. 21:1–62020;

35. Hunter JD. Matplotlib: A 2D Graphics Environment. *Computing in Science Engineering*. 2007; doi: 10.1109/MCSE.2007.55.

36. Pedregosa F, Varoquaux G, Gramfort A, Michel V, Thirion B, Grisel O, et al.. Scikit-learn: Machine Learning in Python. *J Mach Learn Res*. 12:2825–302011;

37. Bankhead P, Loughrey MB, Fernández JA, Dombrowski Y, McArt DG, Dunne PD, et al.. QuPath: Open source software for digital pathology image analysis. *Sci Rep*. 2017; doi: 10.1038/s41598-017-17204-5.

38. Brennan CW, Verhaak RGW, McKenna A, Campos B, Noushmehr H, Salama SR, et al.. The somatic genomic landscape of glioblastoma. *Cell*. 2013; doi: 10.1016/j.cell.2013.09.034.

39. : cBioPortal for Cancer Genomics. https://www.cbioportal.org/ Accessed 2023 Aug 3.

40. : GDC. https://portal.gdc.cancer.gov/ Accessed 2023 Aug 3.

41. Bhowal P, Sen S, Velasquez JD, Sarkar R. Fuzzy ensemble of deep learning models using choquet fuzzy integral, coalition game and information theory for breast cancer histology classification. *Expert Syst Appl*. 2022; doi: 10.1016/j.eswa.2021.116167.

42. Xue D, Zhou X, Li C, Yao Y, Rahaman MM, Zhang J, et al.. An Application of Transfer Learning and Ensemble Learning Techniques for Cervical Histopathology Image Classification. *IEEE Access*. 2020; doi: 10.1109/ACCESS.2020.2999816.

43. Shaban M, Awan R, Fraz MM, Azam A, Tsang Y-W, Snead D, et al.. Context-Aware Convolutional Neural Network for Grading of Colorectal Cancer Histology Images. *IEEE*

Trans Med Imaging. 2020; doi: 10.1109/TMI.2020.2971006.

44. Becker AP, Sells BE, Haque SJ, Chakravarti A. Tumor Heterogeneity in Glioblastomas: From Light Microscopy to Molecular Pathology. *Cancers* . 2021; doi: 10.3390/cancers13040761.

45. Engler JR, Robinson AE, Smirnov I, Hodgson JG, Berger MS, Gupta N, et al.. Increased microglia/macrophage gene expression in a subset of adult and pediatric astrocytomas. *PLoS One*. 2012; doi: 10.1371/journal.pone.0043339.

46. Rao A, Barkley D, França GS, Yanai I. Exploring tissue architecture using spatial transcriptomics. *Nature*. 2021; doi: 10.1038/s41586-021-03634-9.

47. Moses L, Pachter L. Museum of spatial transcriptomics. *Nat Methods*. 2022; doi: 10.1038/s41592-022-01409-2.

48. Roetzer-Pejrimovsky T: GBMatch_CNN: Predicting TS & risk from glioblastoma WSI. https://github.com/tovaroe/GBMatch_CNN Accessed 2023 Aug 24.

49. : GBMatch Supplementary Website. The DNA methylation landscape of glioblastoma disease progression shows extensive heterogeneity in time and space - Supplementary Website. https://www.medical-epigenomics.org/papers/GBMatch/ Accessed 2023 Aug 24.

50. Roetzer-Pejrimovsky T: GBMatch_CNN - additional data. https://zenodo.org/record/8358673. 2023; doi: 10.5281/zenodo.8358673

Supplement

	CD163	CD3	CD34	CD68	CD8	HLA-DR	MIB
Classical	26	27	29	26	27	24	28
Mesenchymal	46	45	50	45	44	43	46
Proneural	31	36	35	34	34	31	29
high risk	36	42	39	40	39	39	39
low risk	46	49	51	46	47	47	46

Table S1. Number of stained slides included in the analysis of the tumor microenvironment for each region and staining.

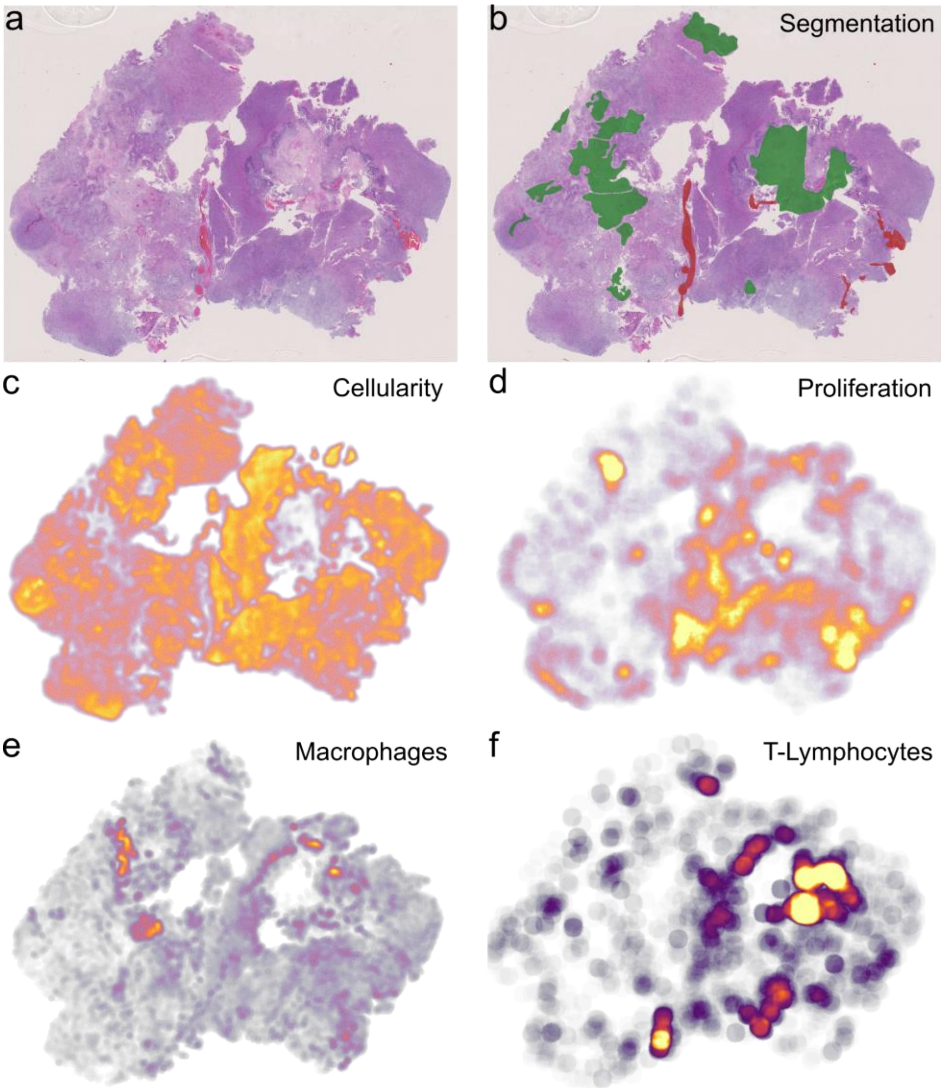

Fig. S1 Glioblastoma intratumoral heterogeneity. (a) Overview of an H&E stained section of a FFPE sample. (b) Manual segmentation overlay (necrosis: green overlay, bleeding: red overlay). (c) Cellularity heatmap highlighting heterogeneously distributed, cell-dense tumor areas. (d) Proliferation as measured by Ki-67 antigen expression (e) Macrophages as CD68-expressing cells surrounding necrosis and in single hot-spots. (f) T-Lymphocytes (CD8-expressing cells) with similar patchy distribution.

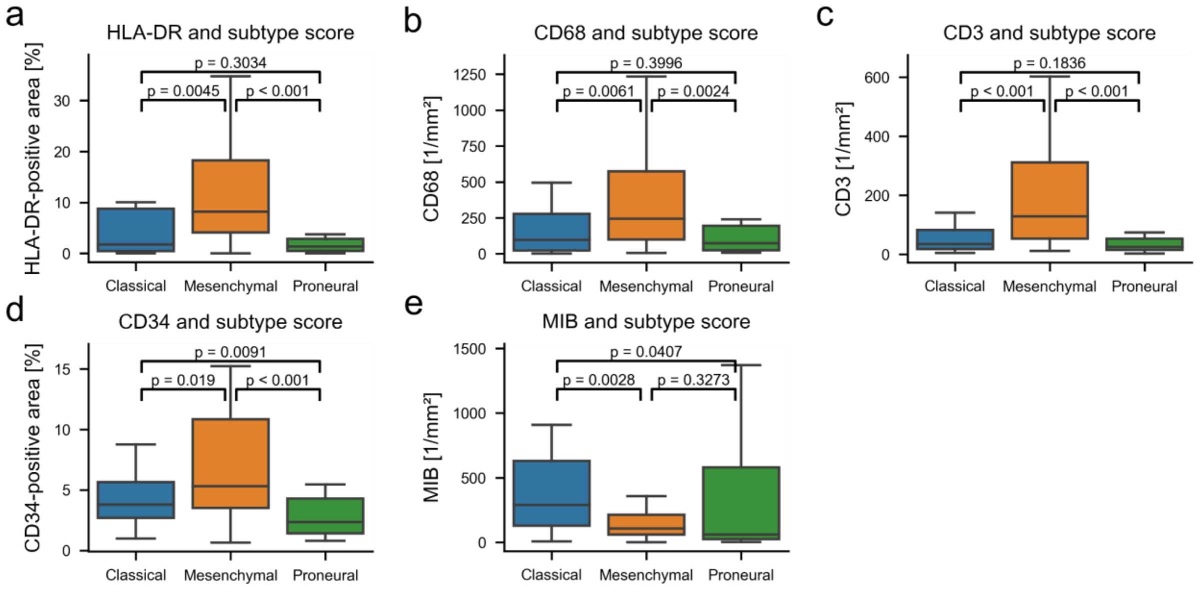

Fig. S2 (a-e) Boxplots of different immunohistochemical stainings in different predicted TS (all p-values calculated with MWU).

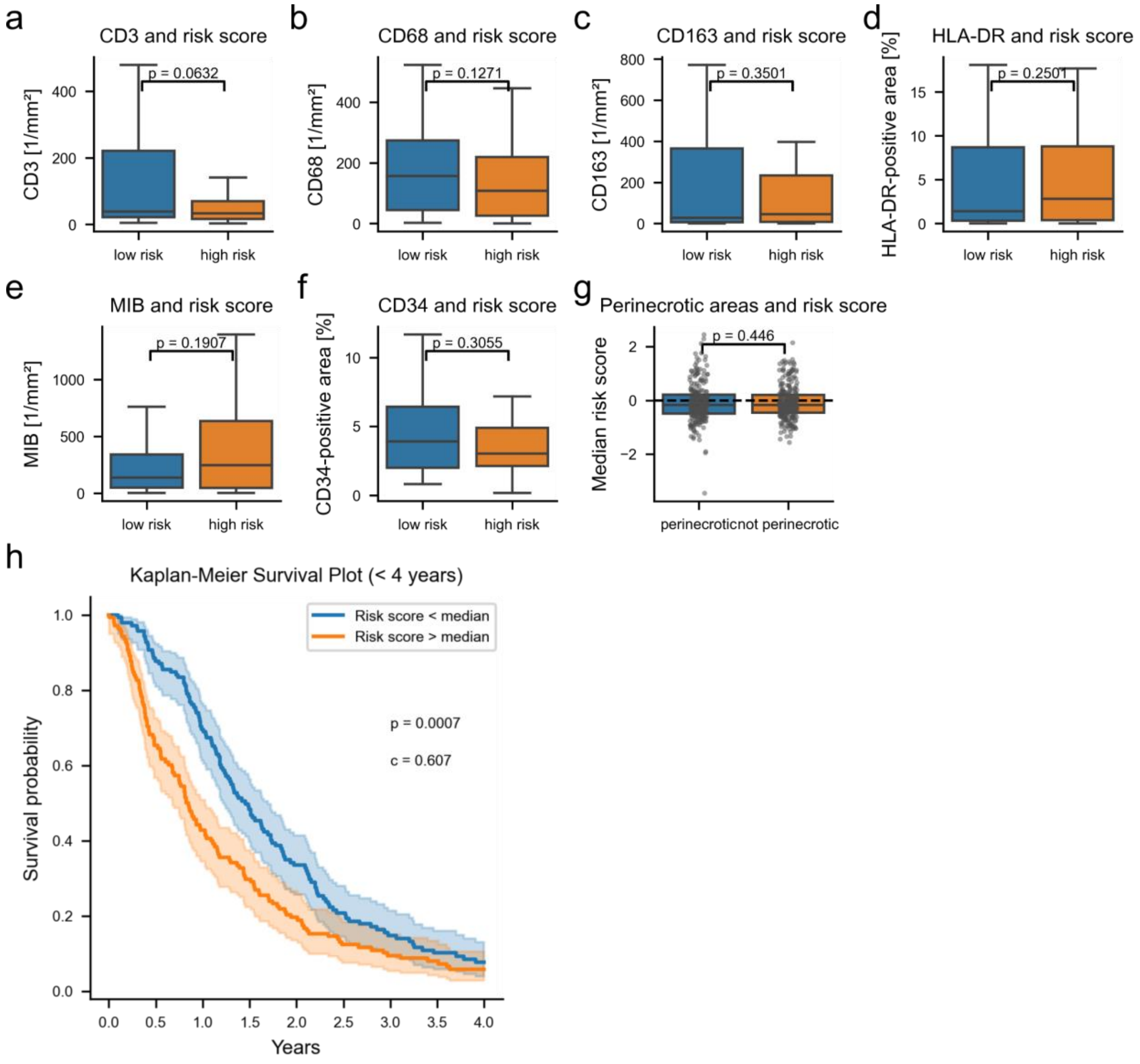

Fig. S3 (a-f) Boxplots of different immunohistochemical stainings by risk class (p-values calculated with MWU). (g) Boxplot of the risk scores in perinecrotic / non-perinecrotic tissue (p-value calculated with MWU). (h) Kaplan-Meier survival plot using the median risk score of all patients as a cut-off for

grouping.

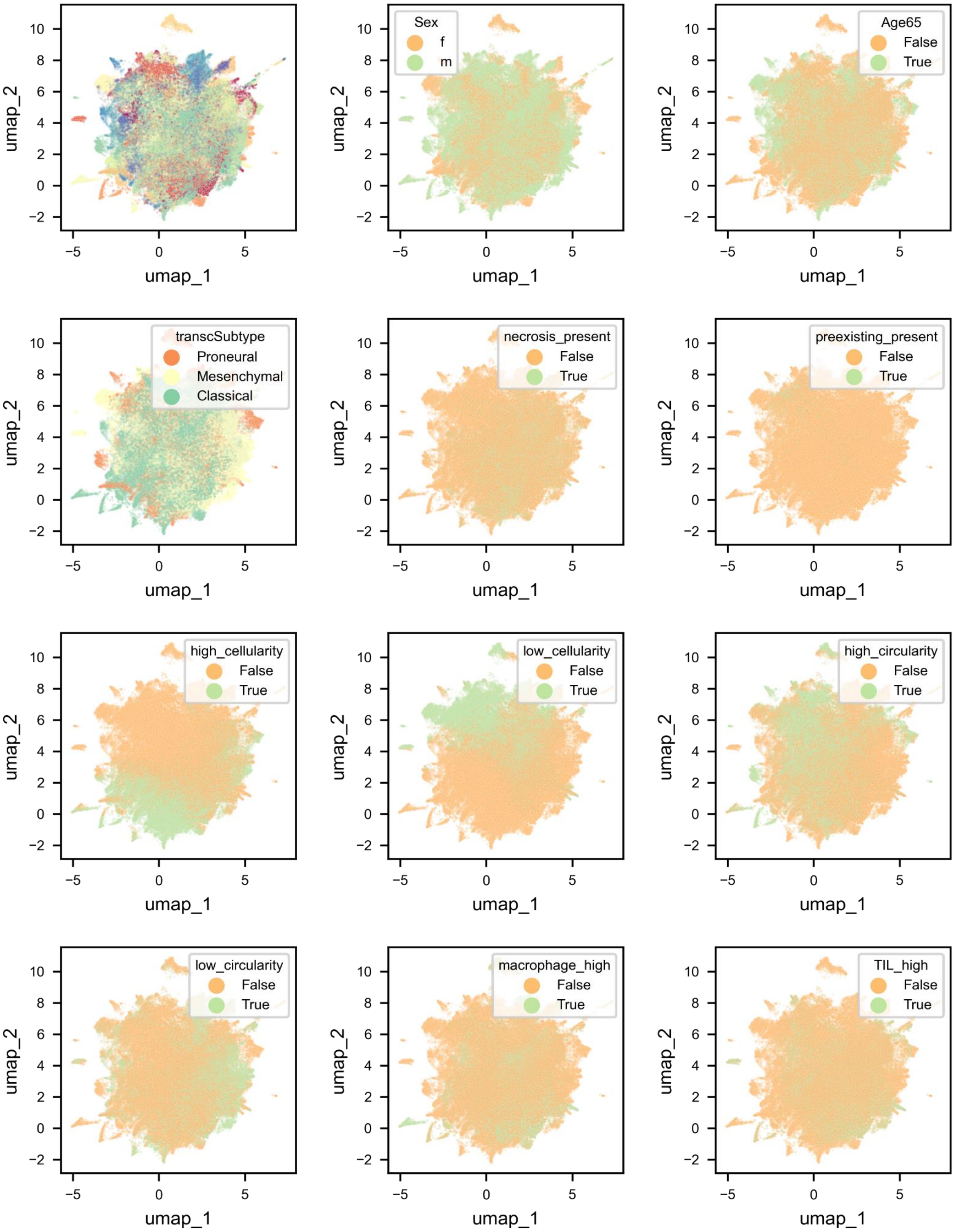

Fig S4 UMAPs with different grouping variables

Click here to access/download;Figure;Figure 1.png

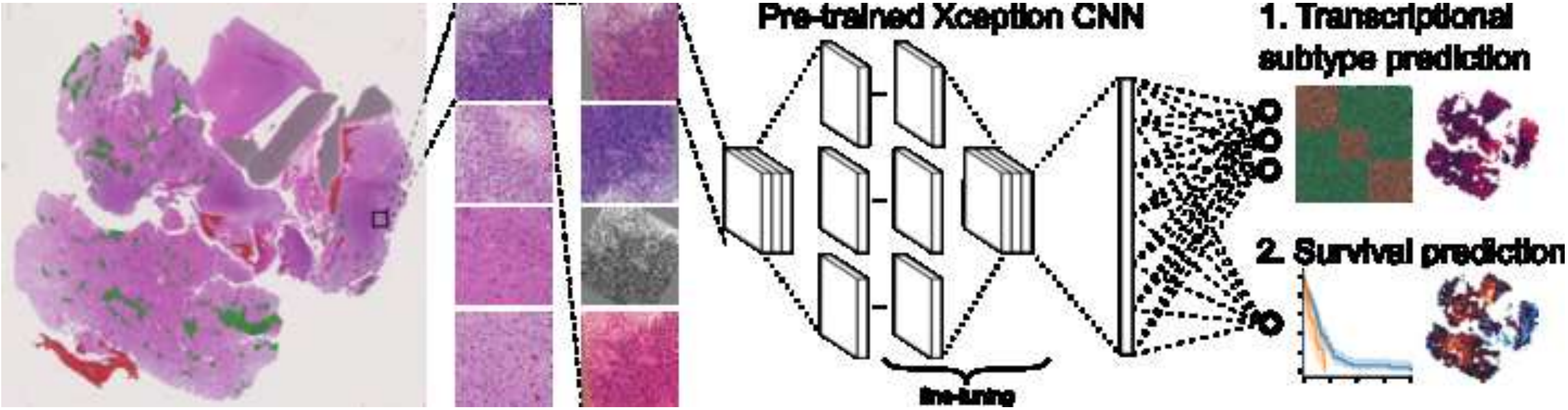

Click here to access/download;Figure;Figure 2.png

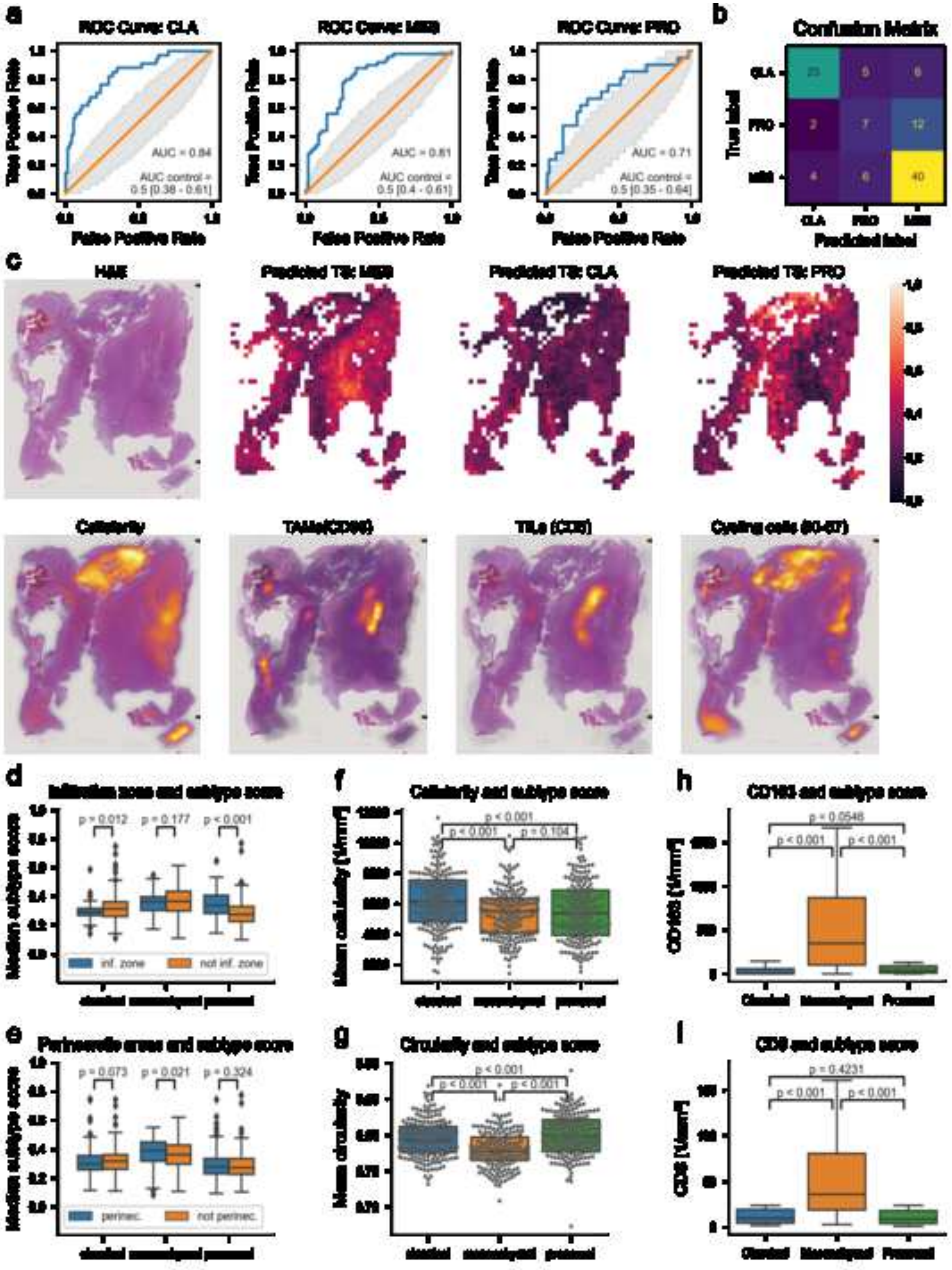

Click here to access/download;Figure;Figure 3.png

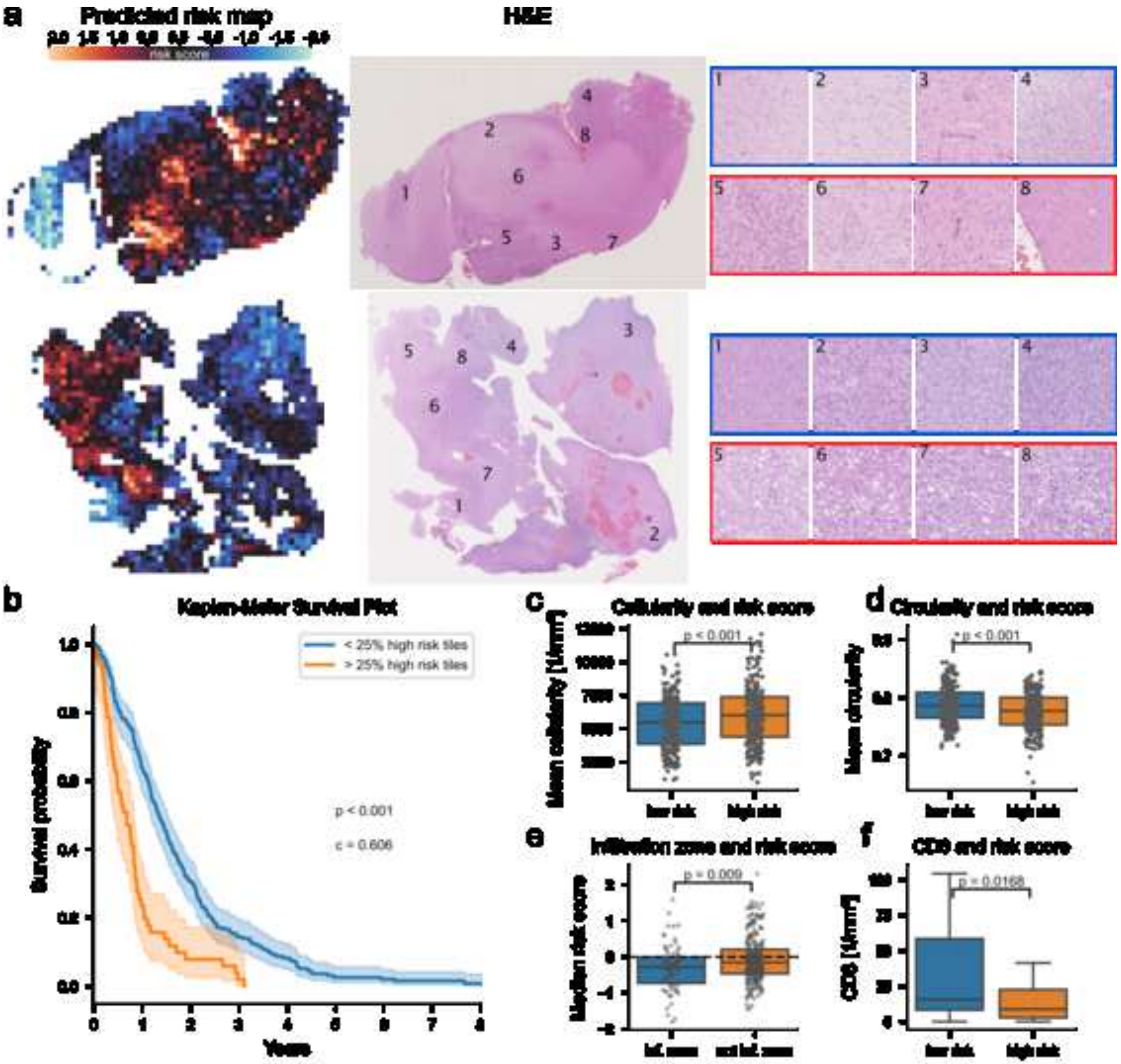

Click here to access/download;Figure;Figure 4.png

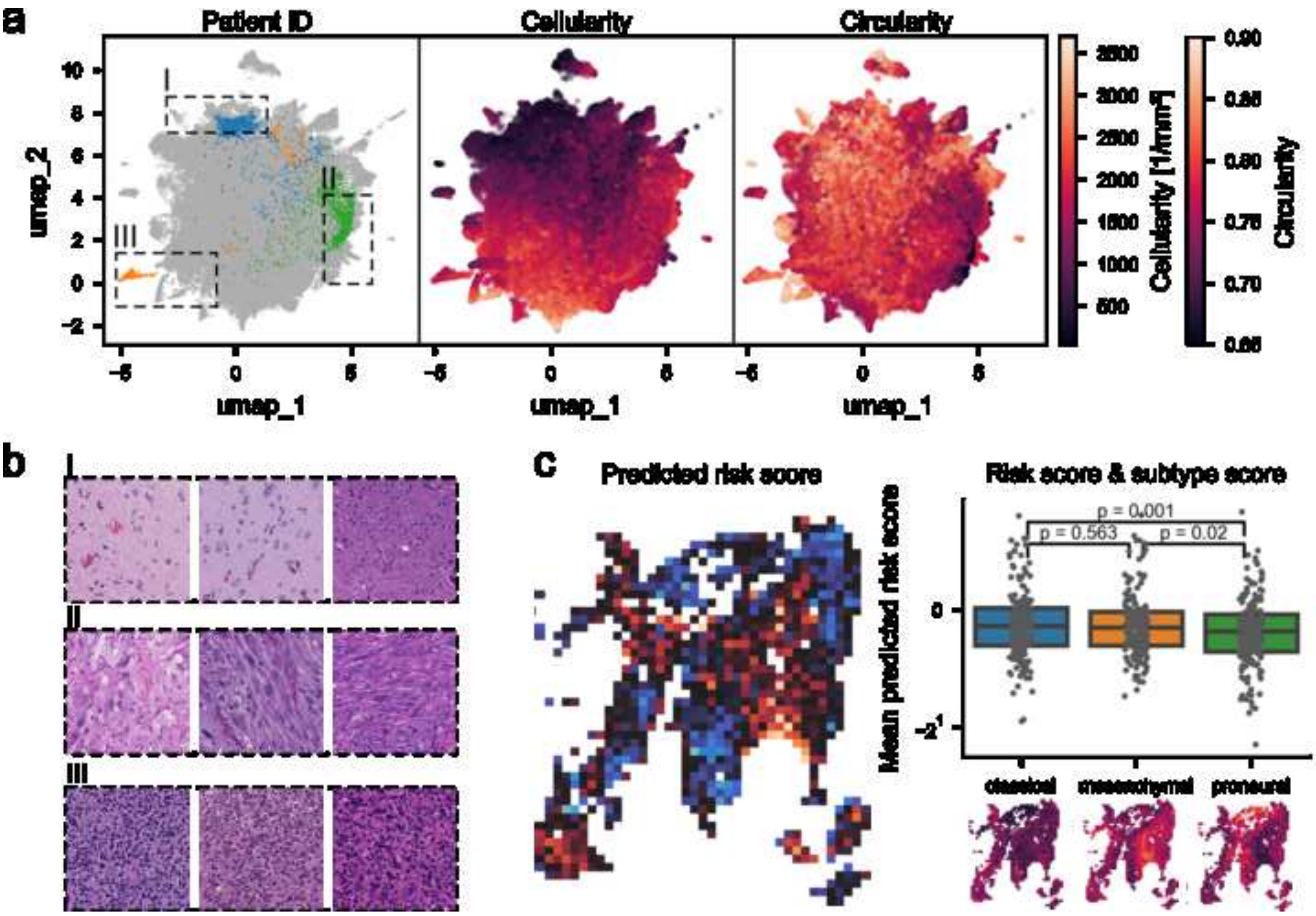

Click here to access/download;Figure;Figure 5.png

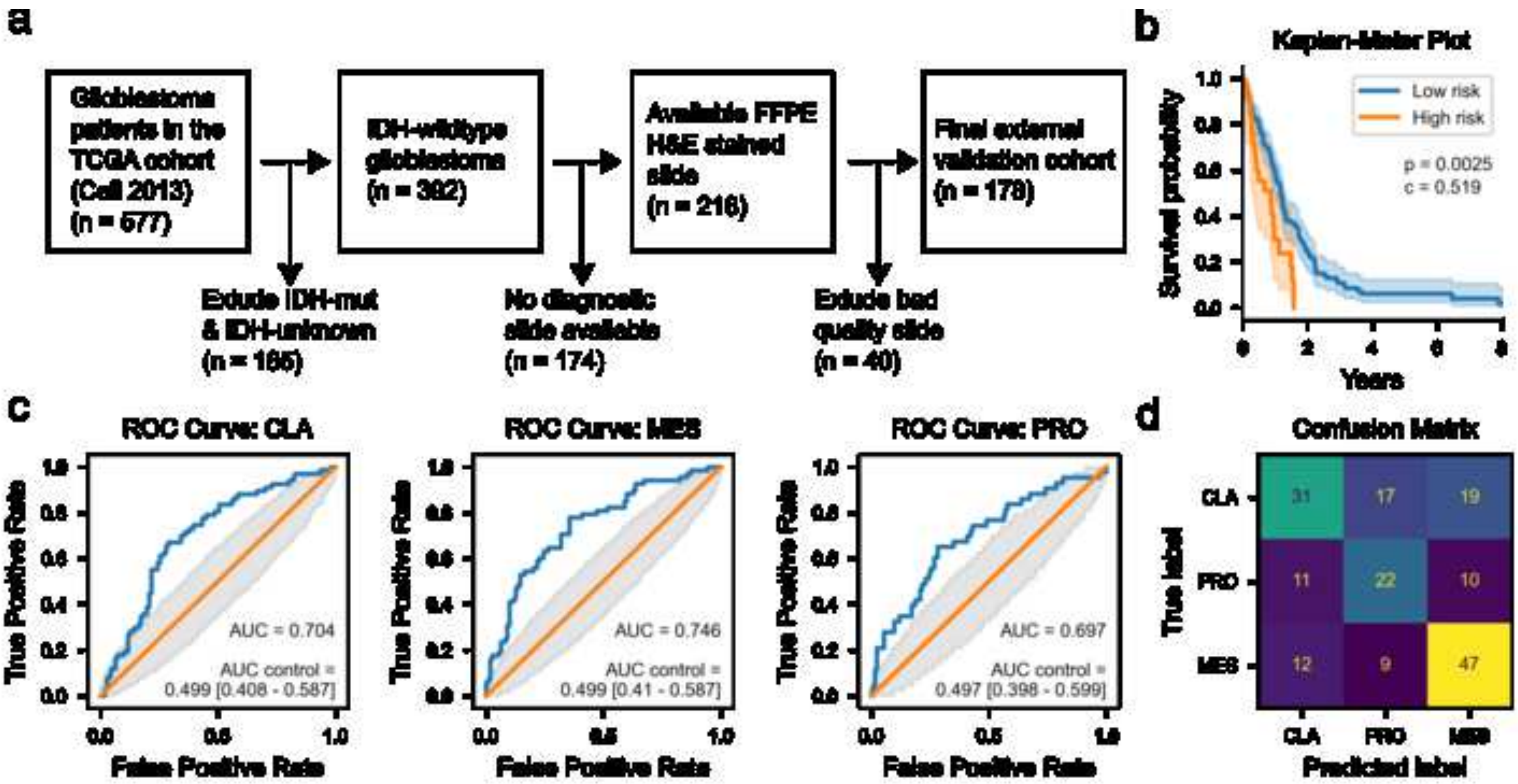

Click here to access/download;Figure;Figure 6.png

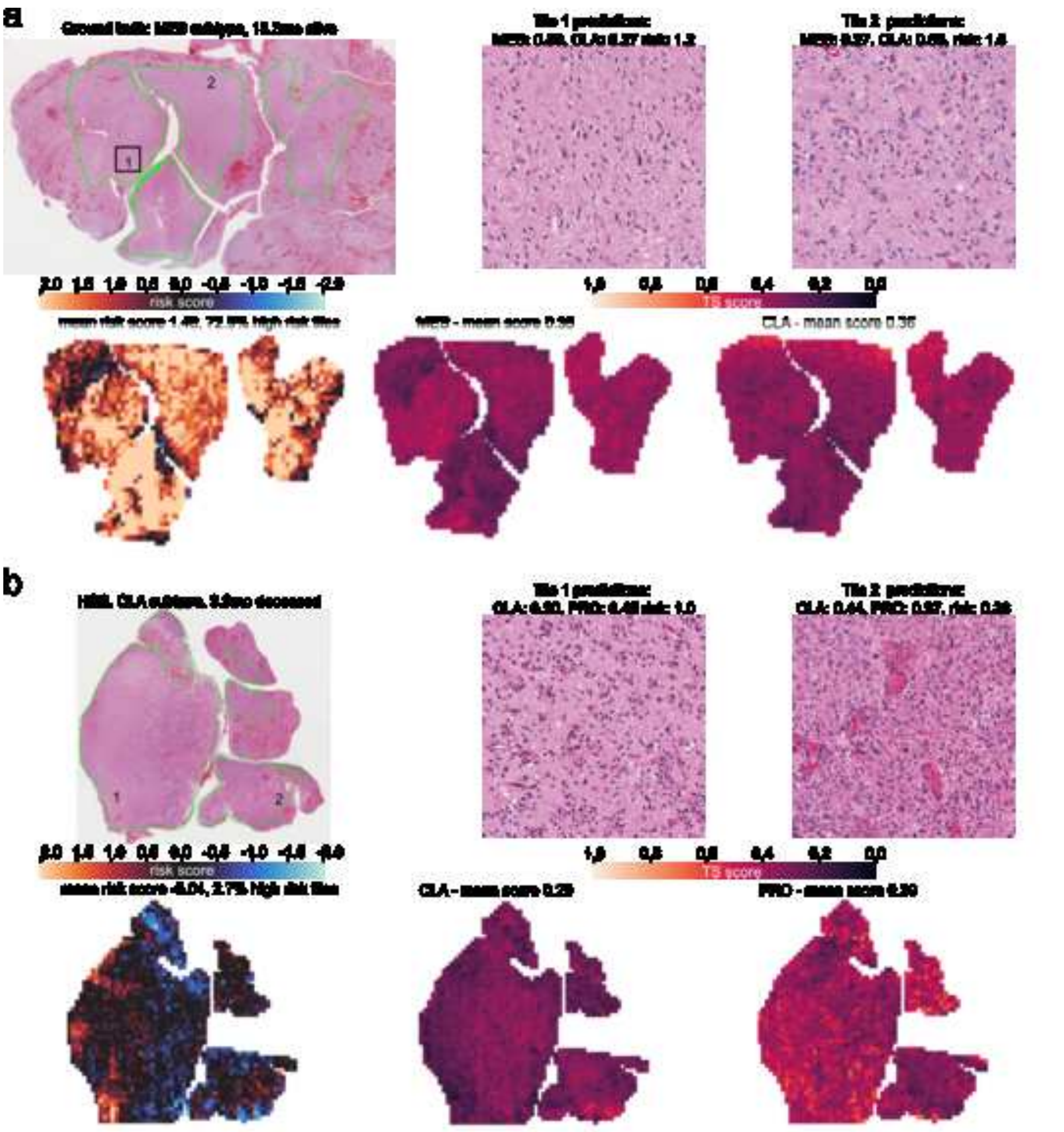

Click here to access/download
Supplementary Material
Table S1 supplementary material.csv

Click here to access/download
Supplementary Material
Figure S1 supplementary material.png

Click here to access/download
Supplementary Material
Figure S2 supplementary material.png

Click here to access/download
Supplementary Material
Figure S3 supplementary material.png

Click here to access/download
Supplementary Material
Figure S4 supplementary material.png
