# Supplementary material for: Deep learning links localized digital pathology phenotypes with transcriptional subtype and patient outcome in glioblastoma
Source: Gigascience. 2024 Aug 26;13:giae057. doi: 10.1093/gigascience/giae057 (PMC11345537; doi:10.1093/gigascience/giae057)
Supplement: giae057_Supplemental_Files [file giae057_supplemental_files.zip › Supplementary figure captions supplementary material.docx]

**Fig. S1** Glioblastoma intratumoral heterogeneity. (a) Overview of an H&E stained section of a FFPE sample. (b) Manual segmentation overlay (necrosis: green overlay, bleeding: red overlay). (c) Cellularity heatmap highlighting heterogeneously distributed, cell-dense tumor areas. (d) Proliferation as measured by Ki-67 antigen expression (e) Macrophages as CD68-expressing cells surrounding necrosis and in single hot-spots. (f) T-Lymphocytes (CD8-expressing cells) with similar patchy distribution.

**Fig. S2** (a-e) Boxplots of different immunohistochemical stainings in different predicted TS (all p-values calculated with MWU).

**Fig. S3** (a-f) Boxplots of different immunohistochemical stainings by risk class (p-values calculated with MWU). (g) Boxplot of the risk scores in perinecrotic / non-perinecrotic tissue (p-value calculated with MWU). (h) Kaplan-Meier survival plot using the median risk score of all patients as a cut-off for grouping.

**Fig S4** UMAPs with different grouping variables
