# Supplementary material for: Deep learning links localized digital pathology phenotypes with transcriptional subtype and patient outcome in glioblastoma
Source: Gigascience. 2024 Aug 26;13:giae057. doi: 10.1093/gigascience/giae057 (PMC11345537; doi:10.1093/gigascience/giae057)
Supplement: giae057_Supplemental_Files [file giae057_supplemental_files.zip › Tables S1 & S2 supplementary material.docx]

|  | **CD163** | **CD3** | **CD34** | **CD68** | **CD8** | **HLA-DR** | **MIB** |
| --- | --- | --- | --- | --- | --- | --- | --- |
| **Classical** | 26 | 27 | 29 | 26 | 27 | 24 | 28 |
| **Mesenchymal** | 46 | 45 | 50 | 45 | 44 | 43 | 46 |
| **Proneural** | 31 | 36 | 35 | 34 | 34 | 31 | 29 |
| **high risk** | 36 | 42 | 39 | 40 | 39 | 39 | 39 |
| **low risk** | 46 | 49 | 51 | 46 | 47 | 47 | 46 |

Table S1. Number of stained slides included in the analysis of the tumor microenvironment for each region and staining.

| N = 41 | **HR** | **p-value** |
| --- | --- | --- |
| **Age** | 1.029 (1.000 - 1.058) | 0.048 |
| **Male sex** | 0.54 (0.26 - 1.10) | 0.091 |
| **Radiochemotherapy (TMZ)** | 0.11 (0.04 - 0.34) | < 0.001 |
| **RS CNN** | 1.09 (0.42 - 2.82) | 0.855 |
| **Methylated MGMT promoter** | 0.49 (0.15 - 1.01) | 0.052 |

Table S2. Cox multivariable survival model including MGMT status in a subset of 41 patients. HR for age is calculated for each 1-year increase of patient age.
