# Supplementary figures and images for: Deep learning links localized digital pathology phenotypes with transcriptional subtype and patient outcome in glioblastoma
Source: Gigascience. 2024 Aug 26;13:giae057. doi: 10.1093/gigascience/giae057 (PMC11345537; doi:10.1093/gigascience/giae057)

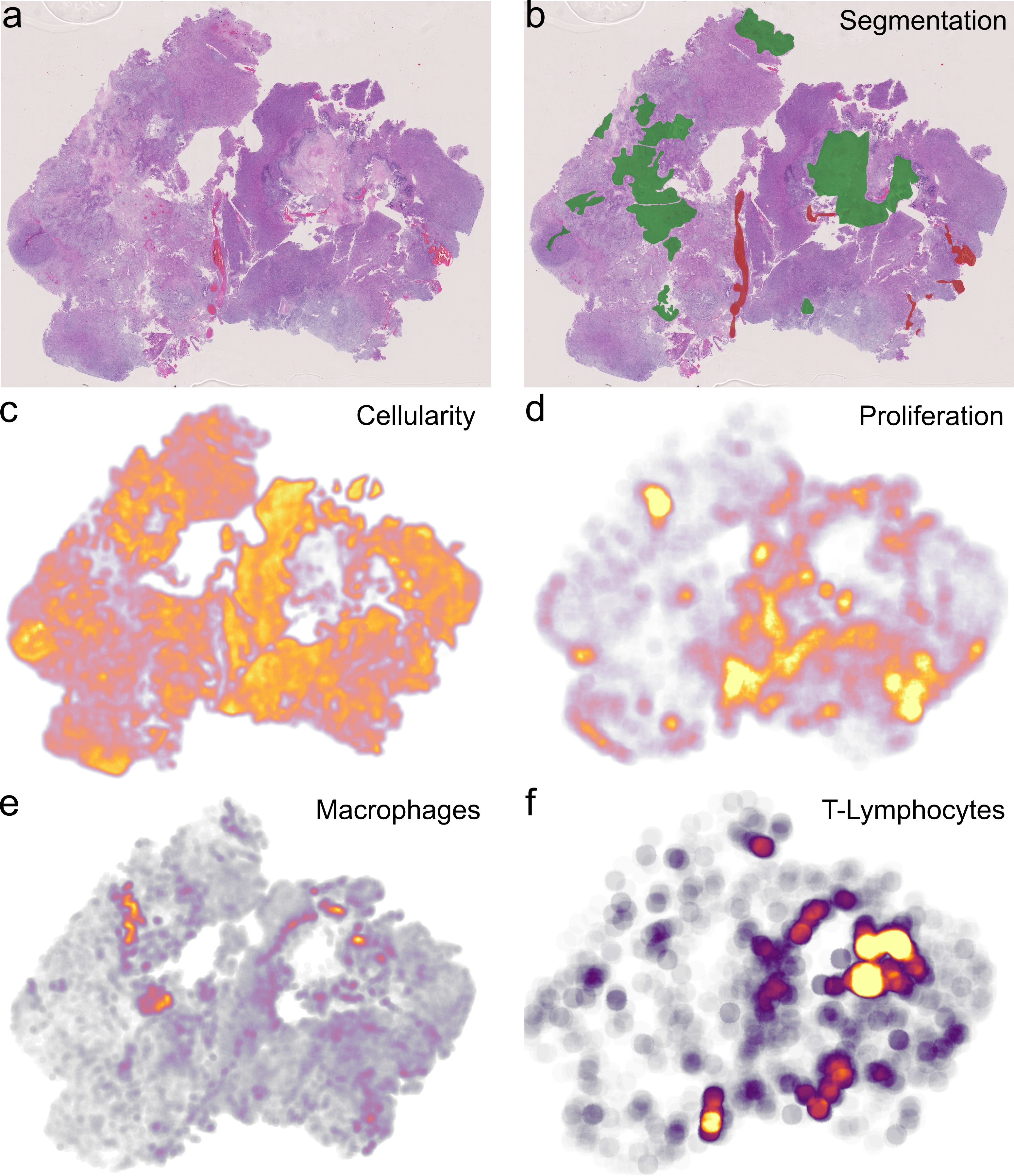

Supplement: giae057_Supplemental_Files [file giae057_supplemental_files.zip › Figure S1 supplementary material.png]

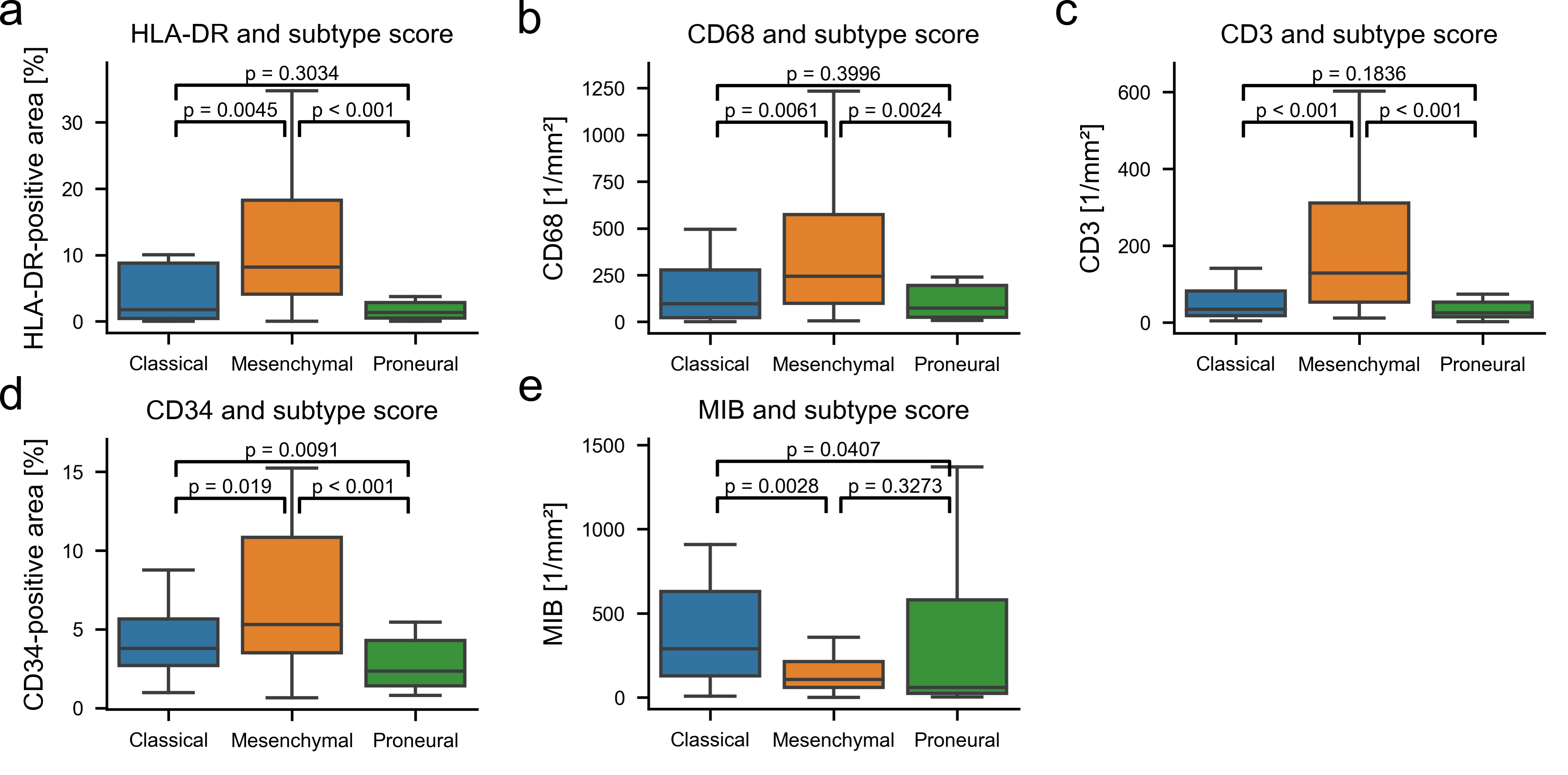

Supplement: giae057_Supplemental_Files [file giae057_supplemental_files.zip › Figure S2 supplementary material.png]

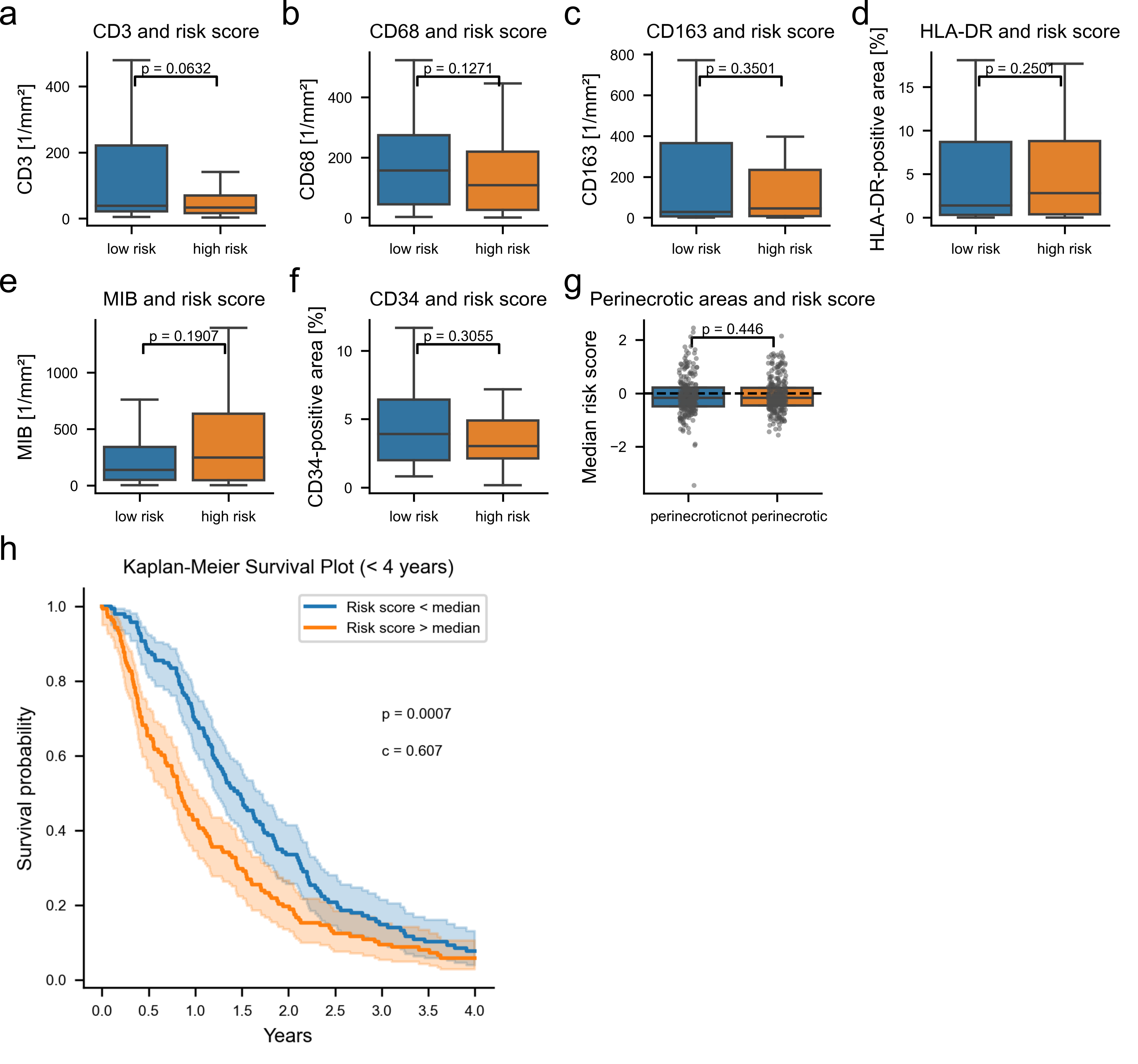

Supplement: giae057_Supplemental_Files [file giae057_supplemental_files.zip › Figure S3 supplementary material.png]

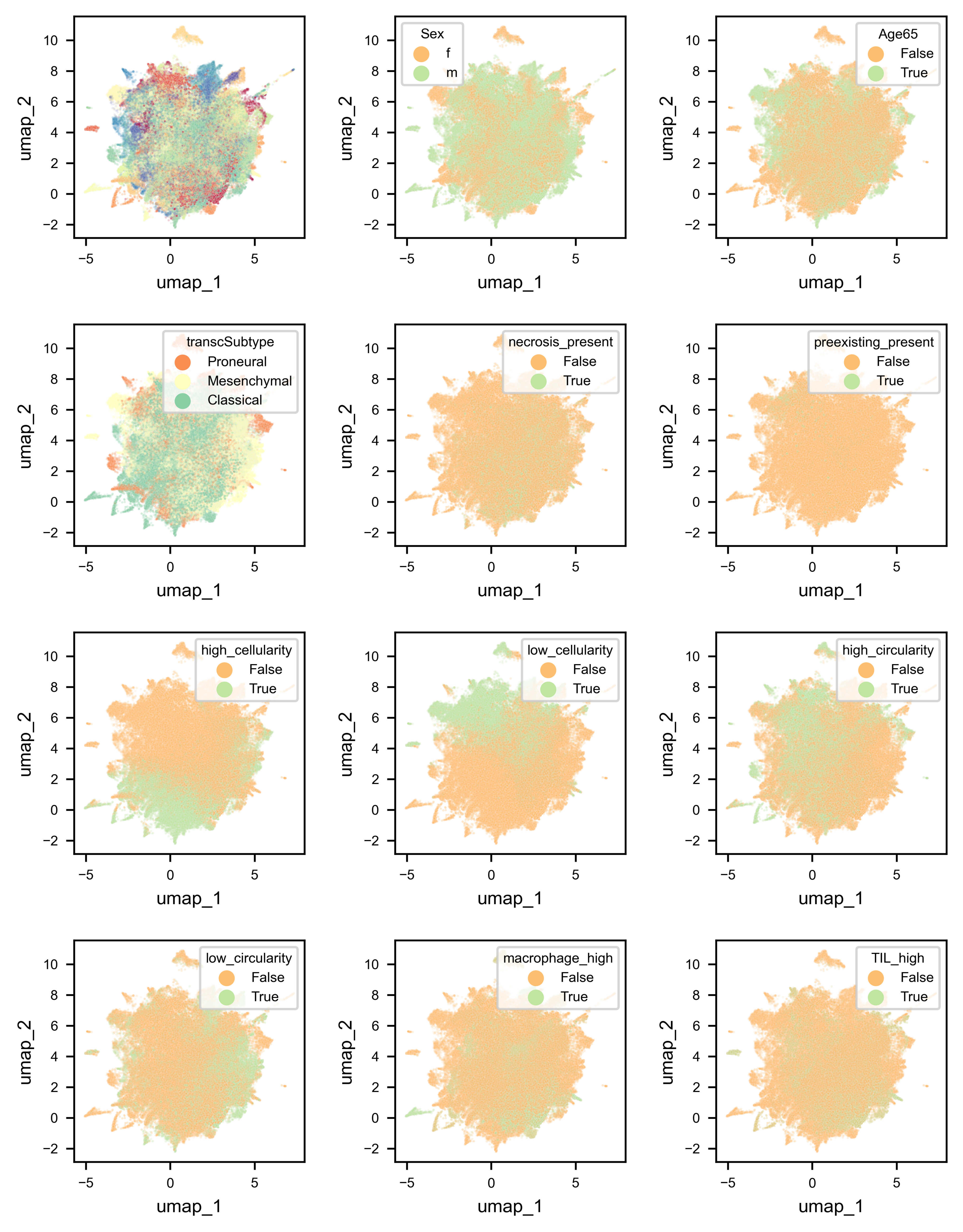

Supplement: giae057_Supplemental_Files [file giae057_supplemental_files.zip › Figure S4 supplementary material.png]
