# Supplementary material for: Deep learning links localized digital pathology phenotypes with transcriptional subtype and patient outcome in glioblastoma
Source: Gigascience. 2024 Aug 26;13:giae057. doi: 10.1093/gigascience/giae057 (PMC11345537; doi:10.1093/gigascience/giae057)
Supplement: giae057_Response_to_Reviewer_Comments_Original_Submission [file giae057_response_to_reviewer_comments_original_submission.pdf]

Author's Response To Reviewer Comments

Dear Mrs. Nogoy,

Thank you for considering our manuscript entitled “Deep learning links localized digital pathology phenotypes with transcriptional subtype and patient outcome in glioblastoma” for publication in GigaScience.

We have carefully revised our manuscript according to your and the reviewers’ comments. In particular, we’ve registered the computational workflow of our project on workflowhub.eu (https://doi.org/10.48546/WORKFLOWHUB.WORKFLOW.883.1) and GBMPredictor as a software application on bio.tools (biotools: gbmpredictor, https://bio.tools/gbmpredictor) and SciCrunch.org (RRID: SCR_025316) and included the identifiers in the manuscript in the “Availability of Source Code and Requirements” section.

Please find a point-to-point response to the reviewers’ questions and comments below. For better readability, we have also uploaded the "response to reviewers" as supplementary material, which includes one additional table and one figure.

Reviewer #1

1. The authors may need to review the literatures on the current state-of-the-arts in histology-based prognostic biomarker development in GBM. As mentioned in the Background section: "So far, no histology-based prognostic biomarker is available", this statement is incorrect. Many previous studies, reporting promising histology-related imaging prognostic biomarkers in GBM and different types of gliomas, can be found in PUBMED or using google search. Given this inaccurate statement, the reviewer is not fully convinced about the motivation and design of proposed study.

We thank the reviewer for catching this inaccuracy. Indeed, some recent studies have suggested histological prognostic biomarkers for diffuse gliomas. We have amended the quoted sentence as follows: “Furthermore, multiple studies have highlighted the potential for histology-based prognostic biomarkers for gliomas in general and glioblastoma in particular (Mobadersany et al., 2018; Liu et al., 2023; Luo et al., 2023; Zheng et al., 2023).”

2. The choice of Xception model was unjustified and the RS-CNN model development was insufficiently described. Xception is a great deep learning model developed a while back, the choice of Xception model for the proposed task needs to be justified.

We thank the reviewer for raising this important point. Indeed, there are more novel models available, most notably pathology-specific pre-trained models that may serve as efficient feature extractors. We chose the Xception architecture for this task as it is relatively light-weight, has been successfully applied previously for classification tasks in histopathology and may outperform the more commonly used ResNet architecture (Shaban et al., 2020; Hameed et al., 2022; Sharma and Kumar, 2022; Yan et al., 2023). Furthermore, (to our knowledge) all published pathology-specific pre-trained models have been pre-trained on TCGA data. However, in our study design, TCGA data is used as an external independent test cohort. Using a TCGA-pretrained model would result in mixing of the training and test data and we thus opted for the ImageNet-pretrained model.
Overall, we believe that while the Xception model is a few years old, it may still serve as a robust model for the development of deep learning workflows. In our revised manuscript, we discuss the issue of the deep learning architecture choice in more detail as follows:
“We used TensorFlow 2.1.0 / keras for developing our deep learning pipeline (Abadi et al., 2016). As a base model, we used an Xception architecture (Chollet, 2016) pre-trained on ImageNet available via the keras model applications (Chollet, 2015). This architecture was chosen because of its high efficiency with similar or increased performance compared to other more commonly used architectures like ResNet

(Chollet, 2016; Yan et al., 2023). We refrained from using other models that were pre-trained with histological data instead of the more generic ImageNet data as they incorporated data from TCGA for the training. Since we here use TCGA data for independent external validation, using the same data for pre-training and validation would result in mixing of training and test data.”

3. In addition, it is not clear how the RS-CNN model was trained towards the survival outcome (i.e., time and event data). Did the author add another cox-regression layer to the network?

We thank the reviewer for pointing out this unclarity. Indeed, we added a final cox-regression layer and used the negative partial log likelihood as the loss function, in a similar approach as Mobadersany et al. (Mobadersany et al., 2018) and have clarified this in the revised manuscript.

4. The multicox regression as shown in Table 3 is inappropriate. Important prognostic factors, including MGMT status, IDH mutation, G-CIMP, ATRX mutation, 1p/19q status, etc., were not included, without which, the independent value of RS CNN remains unjustified.

Including all relevant prognostic factors in the cox model is indeed an important issue. The cohort we used included only glioblastomas, IDH-wildtype (non-G-CIMP) and we’ve clarified this in the revised manuscript. For those bona fide IDH-wildtype glioblastomas, ATRX mutations and 1p/19q codeletions are exceedingly rare and do not have the prognostic relevance as in IDH-mutant gliomas. We concur that the MGMT status would be an important additional factor for the cox model. The retrospective cohort we used as a discovery cohort comprises patients diagnosed before 2016 and MGMT status is available only in only a subset of patients. Moreover, reduced representation bisulfite sequencing was used for the technically challenging task of MGMT status assessment, for which other methods such as pyrosequencing would nowadays be favoured due to their higher accuracy and validity. Finally, there is insufficient FFPE material left to re-assess MGMT status with more precise methods. Given these caveats, we have re-calculated the cox proportional hazards model, including patients with RRBS-derived MGMT promoter status (see table in "response to reviewers" in the supplementary material). In this model, only Radiochemotherapy and age remain statistically significant prognostic factors. The predicted risk score and MGMT promoter methylation status fail to reach statistical significance, most likely due to the very limited sample size (41 patients compared to 262 patients in the initial model).
We thus argue to keep our previous Cox model in the manuscript, particularly due to sample size issues. To ensure its clinical relevance, this model includes the two other most important prognostic factors, i.e., receipt of chemoradiotherapy and patient age.

5. The external validation on TCGA-GBM cohort did not support the major conclusion. Specifically, the risk factor did not provide independent and significant prognostic value, and the predictive power towards transcriptional subtypes (although better than random guess) is far from the state-of-the-arts that have already been reported in literature (see comment #1). Given the validation outcome, the proposed work seems provide very limited clinical value.

The reviewer raises an important issue, which we specifically pointed out and discussed in the manuscript. We also want to stress that the cut-off for the risk score defined in the training set still separates patients by survival in the independent test set (Fig 1b). Thus, even though the importance of the risk score does not hold true in the regression analysis, the main finding of diverging Kaplan Meier curves was successfully replicated in the external validation set.

Reviewer #3
1. In this study, the authors proposed RS/TS-CNNs and validated them using reasonably large "discovery cohort" and "validation cohort" collected from publicly available datasets for glioblastoma patients' prognostic and transcriptional subtype predictions. Both investigations are very clinically relevant. The study is well-executed, and the interpretations are supported by thoughtful post-hoc data analytics.

Glioblastoma, one of the most prevalent and lethal CNS tumors, exhibits high inter- and intra-tumoral heterogeneity. One major contribution of this work is the use of widely available histology images to infer transcriptomic markers for patients, since transcriptomic profiling remains expensive and is not widely adopted in routine examinations in many geographical regions. The authors showed the potential of deep learning to identify and map transcriptional subtypes—classical, mesenchymal, and proneural—to cellular morphological architecture, which could be useful for prognostic prediction and studies towards tumor microenvironment (Zheng et al., Spatial cellular architecture predicts prognosis in glioblastoma). While similar downstream tasks using histology images alone or in conjunction with biomarkers from gene panels have been explored (Chen et al., Pan-cancer integrative histology-genomic analysis via multimodal deep learning and subsequent works by other research groups in CVPR etc.), RS-CNN, built upon the Xception backbone pre-trained on ImageNet, captures clinically meaningful prognostic features, especially when generalized across different data sites.

We appreciate the concise summary of the relevant context and the positive comments.

2. To further provide insights to a broad audience, there are two major suggestions that the authors might consider including. First, the field of computational pathology seems to have shifted towards leveraging the benefits of pre-trained, self-supervised models as feature extractors. Considering the authors followed a similar two-step workflow (freezing the pre-trained network as a feature extractor, then training a head classifier), they could explore replacing the ImageNet pre-trained model with two published works, as literature has reported their advancements over ImageNet pre-trained extractors. This may further improve the performance of TS-CNN (Wang et al., Transformer-based Unsupervised Contrastive Learning for Histopathological Image Classification; Kang et al., Benchmarking Self-Supervised Learning on Diverse Pathology Datasets). A brief justification would also be valuable if the performance shows no significant difference.

The choice of the model architecture and the pre-training is indeed an important issue. We've initially chosen the Xception architecture as it is relatively light-weight and a few recent publications at the time of our project conception underscored its usefulness for histopathology (Shaban et al., 2020; Hameed et al., 2022; Sharma and Kumar, 2022; Yan et al., 2023).
Following the recommendation of the reviewer, we replaced the Xception backbone with the suggested feature extractors (ResNet and ViT/S) from Kang et al. (Kang et al., 2022). Using this setup, our model fails to meaningfully predict the transcriptional subtypes from H&E sections alone (see figure in "response to reviewers" - supplementary material).
This result is surprising, as one would have expected to achieve at least en-par performance and requires some justification. There are two main points that might explain this discrepancy. First, there are important architectural differences between Xception and ResNet (and of course even more so compared to visual transformers). In particular, Xception outperforms ResNet on the ImageNet dataset with fewer trainable parameters (Chollet, 2016) and has also shown better performance in some histopathology tasks (Yan et al., 2023). Moreover, while visual transformers excel at some tasks in comparison to CNNs, there are other contexts in which they don't provide additional value (Deininger et al., 2022). Second, in our initial setup, we did not only use Xception as "frozen" feature extractor, but also performed finetuning of the last layers to enable identification of meaningful histological features. In summary, replacing an ImageNet-trained feature extractor with one that was trained on histological data is in principle promising. However, in the present context, we propose to rely on using TCGA data as an independent external validation cohort. By using TCGA data for both, pre-training and testing, we would face the problem of mixing training and test data. Ultimately, we'd like to point out that there are very recent studies on deep learning for histopathology that use ImageNet-pretrained models (Yan et al., 2023; El Nahhas et al., 2024). We've revised the manuscript to discuss the choice of the ImageNet-pretrained Xception network in more detail and included the most important points mentioned above:
"We used TensorFlow 2.1.0 / keras for developing our deep learning pipeline (Abadi et al., 2016). As a base model, we used an Xception architecture (Chollet, 2016) pre-trained on ImageNet available via the keras model applications (Chollet, 2015). This architecture was chosen because of its high efficiency with similar or increased performance compared to other more commonly used architectures like ResNet (Chollet, 2016; Yan et al., 2023). We refrained from using other models that were pre-trained with histological data instead of the more generic ImageNet data as they incorporated data from TCGA for the training. Since we here use TCGA data for independent external validation, using the same data for pre-

training and validation would result in mixing of training and test data.”

3. Second, while the current exploration and discussion are engaging, discussing some broader impacts would be worthwhile, such as the correlation between the outputs from TS and RS on the same slide, potential integration or comparison with other omics like spatial transcriptomes or genomes, and generalizability to other slide types (e.g., frozen sections and smears).

We thank the reviewer for this important suggestion. In the discussion, we briefly discuss integrating RS and TS and we’ve added another paragraph to discuss promising ways in which histology-based biomarkers could be combined with molecular features at the spatial level and how cryosections could be used to bring integrated histopathological-molecular spatial analysis directly into the operating theatre:

“Predicting risk and transcriptional subtypes from H&E scans opens up many interesting perspectives for future work. Integrating histological features with spatially resolved transcriptomics will help in understanding how cell identity functionally shapes cell morphology; a concept, which can be elegantly extended to further modalities such as spatial proteomics or epigenomics (Dong et al., 2005; Zeng et al., 2022; Davis et al., 2023; Zhang et al., 2023; Zheng et al., 2023). Furthermore, as most spatial molecular profiling techniques are time and cost intensive, they have been mainly applied in research without immediate clinical implications. However, the ability to predict molecular markers directly from H&Es would fuel their translational impact paving the way towards broad and rapid clinical use of novel biomarkers. Importantly, this concept is not limited to FFPE-derived H&Es but could potentially include H&Es from cryosections as a means to support intraoperative integrated diagnostics (Ozyoruk et al., 2022; Nasrallah et al., 2023).”

4. Additionally, sharing the intermediate annotations prepared by the credible clinician team for training TS-CNN with the community would be beneficial, especially since these data are already public. This includes the manual segmentation described in the section "Handling of digital slides," as such efforts can further aid in building cellular quantification tools for evaluating deep learning models for CNS. The authors could consider presenting these annotations as a contribution, referencing Amgad and Cooper's recent work in Nature Medicine on invasive breast cancer.

We fully agree that those recent endeavours to make all raw and intermediate data publicly available are of invaluable importance for the whole community. The raw annotations are already available via the GBMatch webpage and the annotations at the tile level are available via the zenodo repository. Additionally, also the annotations for immunohistochemically stained slides are available via the zenodo repository. Taken together, all raw and intermediate data are publicly available. We have made sure to clarify this in the revised manuscript in the “Data Availability” section:

“Following recent efforts to make all raw and intermediate annotations publicly available for easy re-use (Amgad et al., 2024), The complete slide scan library, including H&E stained slides and intermediate annotations such as corresponding tissue segmentations as well as immunohistochemically stained slides, is available online via the GBMatch supplementary website (GBMatch Supplementary Website, no date; Klughammer et al., 2018). All pre-selected image tiles used for training with their corresponding annotations and segmentations for the immunohistochemically stained slides are available via an accompanying zenodo repository (Roetzer-Pejrimovsky, 2023). The external TCGA validation dataset is available via cBioPortal (cBioPortal for Cancer Genomics, no date) and the GDC Data Portal (GDC, no date).“

5. Besides the works listed above, here are some very relevant works that the authors could also include:
Zeng, Y. et al. Spatial transcriptomics prediction from histology jointly through Transformer and graph neural networks. Brief. Bioinform. 23, bbac297 (2022).
Davis S, Scott C, Oetjen J, Charles PD, Kessler BM, Ansorge O, Fischer R. Deep topographic proteomics of a human brain tumour. Nat Commun. 2023
Shumin Dong, Catherine L. Nutt, Rebecca A. Betensky, Anat O. Stemmer-Rachamimov, Nicholas C. Denko,

Keith L. Ligon, David H. Rowitch, David N. Louis, Histology-Based Expression Profiling Yields Novel Prognostic Markers in Human Glioblastoma, Journal of Neuropathology & Experimental Neurology
Nasrallah MP, Zhao J, Tsai CC, Meredith D, Marostica E, Ligon KL, Golden JA, Yu KH. Machine learning for cryosection pathology predicts the 2021 WHO classification of glioma. Med. 2023
Ozyoruk, K.B., Can, S., Darbaz, B. et al. A deep-learning model for transforming the style of tissue images from cryosectioned to formalin-fixed and paraffin-embedded. Nat. Biomed. Eng (2022)

We thank the reviewer for suggesting these important references and made sure to include and discuss them in our manuscript.

Thank you for your work on this submission!

Best wishes,

Adelheid Woehrer and Thomas Roetzer-Pejrimovsky
Division of Neuropathology and Neurochemistry
Department of Neurology
Medical University of Vienna

References

Abadi, M. et al. (2016) ‘TensorFlow: A System for Large-Scale Machine Learning’, in 12th USENIX symposium on operating systems design and implementation (OSDI 16), pp. 265–283.
Amgad, M. et al. (2024) ‘A population-level digital histologic biomarker for enhanced prognosis of invasive breast cancer’, Nature medicine, 30(1), pp. 85–97.
cBioPortal for Cancer Genomics (no date). Available at: https://www.cbioportal.org/ (Accessed: 3 August 2023).
Chollet, F. (2016) ‘Xception: Deep Learning with Depthwise Separable Convolutions’, arXiv [cs.CV]. Available at: http://arxiv.org/abs/1610.02357.
Chollet, F.A.O. (2015) Keras. Available at: https://keras.io (Accessed: 21 December 2022).
Davis, S. et al. (2023) ‘Deep topographic proteomics of a human brain tumour’, Nature communications, 14(1), p. 7710.
Deininger, L. et al. (2022) ‘A comparative study between vision transformers and CNNs in digital pathology’, arXiv [eess.IV]. Available at: http://arxiv.org/abs/2206.00389.
Dong, S. et al. (2005) ‘Histology-based expression profiling yields novel prognostic markers in human glioblastoma’, Journal of neuropathology and experimental neurology, 64(11), pp. 948–955.
El Nahhas, O.S.M. et al. (2024) ‘Regression-based Deep-Learning predicts molecular biomarkers from pathology slides’, Nature communications, 15(1), p. 1253.
GBMatch Supplementary Website (no date) The DNA methylation landscape of glioblastoma disease progression shows extensive heterogeneity in time and space - Supplementary Website. Available at: https://www.medical-epigenomics.org/papers/GBMatch/ (Accessed: 24 August 2023).
GDC (no date). Available at: https://portal.gdc.cancer.gov/ (Accessed: 3 August 2023).
Hameed, Z. et al. (2022) ‘Multiclass classification of breast cancer histopathology images using multilevel features of deep convolutional neural network’, Scientific reports, 12(1), p. 15600.
Kang, M. et al. (2022) ‘Benchmarking Self-Supervised Learning on Diverse Pathology Datasets’, arXiv [cs.CV]. Available at: http://arxiv.org/abs/2212.04690.
Klughammer, J. et al. (2018) ‘The DNA methylation landscape of glioblastoma disease progression shows extensive heterogeneity in time and space’, Nature medicine, 24(10), pp. 1611–1624.
Liu, X.-P. et al. (2023) ‘Clinical significance and molecular annotation of cellular morphometric subtypes in lower-grade gliomas discovered by machine learning’, Neuro-oncology, 25(1), pp. 68–81.
Luo, C. et al. (2023) ‘Predicting the recurrence and overall survival of patients with glioma based on histopathological images using deep learning’, Frontiers in neurology, 14, p. 1100933.
Mobadersany, P. et al. (2018) ‘Predicting cancer outcomes from histology and genomics using convolutional networks’, Proceedings of the National Academy of Sciences of the United States of America,

115(13), pp. E2970–E2979.
Nasrallah, M.P. et al. (2023) ‘Machine learning for cryosection pathology predicts the 2021 WHO classification of glioma’, Med (New York, N.Y.), 4(8), pp. 526–540.e4.
Ozyoruk, K.B. et al. (2022) ‘A deep-learning model for transforming the style of tissue images from cryosectioned to formalin-fixed and paraffin-embedded’, Nature biomedical engineering, 6(12), pp. 1407–1419.
Roetzer-Pejrimovsky, T. (2023) GBMatch_CNN - additional data. Available at: https://doi.org/10.5281/zenodo.8358673.
Shaban, M. et al. (2020) ‘Context-Aware Convolutional Neural Network for Grading of Colorectal Cancer Histology Images’, IEEE transactions on medical imaging, 39(7), pp. 2395–2405.
Sharma, S. and Kumar, S. (2022) ‘The Xception model: A potential feature extractor in breast cancer histology images classification’, ICT Express, 8(1), pp. 101–108.
Yan, R. et al. (2023) ‘Histopathological bladder cancer gene mutation prediction with hierarchical deep multiple-instance learning’, Medical image analysis, 87, p. 102824.
Zeng, Y. et al. (2022) ‘Spatial transcriptomics prediction from histology jointly through Transformer and graph neural networks’, Briefings in bioinformatics, 23(5). Available at: https://doi.org/10.1093/bib/bbac297.
Zhang, D. et al. (2023) ‘Spatial epigenome-transcriptome co-profiling of mammalian tissues’, Nature, 616(7955), pp. 113–122.
Zheng, Y. et al. (2023) ‘Spatial cellular architecture predicts prognosis in glioblastoma’, Nature communications, 14(1), p. 4122.
