# Supplementary material for: Deep learning links localized digital pathology phenotypes with transcriptional subtype and patient outcome in glioblastoma
Source: Gigascience. 2024 Aug 26;13:giae057. doi: 10.1093/gigascience/giae057 (PMC11345537; doi:10.1093/gigascience/giae057)
Supplement: giae057_Response_to_Reviewer_Comments_Revision_1 [file giae057_response_to_reviewer_comments_revision_1.pdf]

Author's Response To Reviewer Comments

Dear Mrs. Nogoy,

Thank you again for considering our manuscript entitled “Deep learning links localized digital pathology phenotypes with transcriptional subtype and patient outcome in glioblastoma” for publication in GigaScience. Please find our response to your and the reviewers' comments attached below or in the "Response to Reviewers 2.docx"-file.

We have carefully revised our manuscript to address your and the reviewers’ remaining suggestions. In particular, we have performed statistical power calculation for the suggested 41-patient cox regression model. We determined a power of 0.20 for the detection of an association of the predicted risk scores and survival, which we argue provides insufficient statistical power for the multi-cox evaluation. However, to ensure full transparency regarding negative results, we propose to include and discuss both models in the manuscript. That is, we would keep the primary larger model without MGMT status in the main text and provide the secondary smaller model with MGMT status in the supplement. We have also added another paragraph to address this issue:

“Of note, MGMT promoter methylation status was not included in the multivariable model as it was only available for a subset of 41 patients, which would have limited the statistical power for the detection of an association of survival and risk score (power of 0.20 (Hsieh and Lavori, 2000)). In this smaller subset, only age and radiochemotherapy remained as statistically significant prognostic factors, while MGMT status and the risk scores failed to reach statistical significance (table S2). ”

We’ve registered the computational workflow of our project on workflowhub.eu (https://doi.org/10.48546/WORKFLOWHUB.WORKFLOW.883.1) and GBMPredictor as a software application on bio.tools (biotools: gbmpredictor, https://bio.tools/gbmpredictor) and SciCrunch.org (RRID: SCR_025316) and included the identifiers in the manuscript in the “Availability of Source Code and Requirements” section.

Please find a point-to-point response to the reviewers’ questions and comments below.

Reviewer #1:

(1) Independent clinical value: The author mentioned that the predicted risk score does not provide significant clinical value after adjusting for MGMT status due to the limited sample size (41 patients). However, 41 patients should provide sufficient statistical power for the multi-cox evaluation. Please revisit this concern, and provide statistical justification if necessary.

We fully agree that ensuring sufficient statistical power of the performed tests is an important issue. Using the formula of Hsieh and Lavori (Hsieh and Lavori, 2000), we calculated the statistical power of a cox regression model at a significance level of 0.05 and an expected hazard ratio of 1.3 for a sample size of 41 patients to be power = 0.20. Conversely, using the same formula we calculated the needed sample size to ensure a power of 0.8 to be sample_size = 256 (which our initial cox model narrowly exceeds).
However, as this point has also been raised by reviewer #3 and we concur that full transparency of negative results is important, we have amended the manuscript to include a statement on the cox regression model including also MGMT status.

(2) As mentioned in the previous comments, the predictive power towards transcriptional subtypes is far from the state-of-the-arts. However, this comment remains unaddressed. Please review the literature, and provide proper justification on the reported performance.

There has indeed been previous work to address the relationship between histology and transcriptional subtypes. Most notably, one recently published deep learning algorithm achieves high predictive power on

the single cell level (Zheng et al., 2023). However, their study design and main scientific question are different from ours, first and foremost because we aimed to predict transcriptional subtypes on the patient (bulk) level. Other exemplary published works have not used only H&E stained sections, but included immunohistochemical stainings for transcriptional subtypes prediction (Orzan et al., 2020).
Thus, to the best of our knowledge, we believe that the algorithm presented in our manuscript (TS-CNN) contributes substantially to better understanding the interface between histology and molecular biology.

Reviewer #3:

(1) The authors have conducted additional experiments and provided justifications in their writing to address previous comments. This work contributes to the community in two significant ways: (1) defining and developing a prototype deep learning solution that uses histological images and transcriptional subtypes to predict patient outcomes; and (2) providing fine-grained annotations for enabling using histology features to interpret deep learning outputs for Glioblastoma. While I am generally positive, I recommend that the authors conduct another editorial round of revision. Notably, most of the negative experimental results are valuable but have not been presented or discussed in the manuscript. For example, when exploring using MGMT as a prognostic factor in a Cox model, the observations may inspire further investigation by others. The authors state: "Given these caveats, we have recalculated the Cox proportional hazards model, including patients with RRBS-derived MGMT promoter status. In this model, only radiochemotherapy and age remain statistically significant prognostic factors. The predicted risk score and MGMT promoter methylation status fail to reach statistical significance, most likely due to the very limited sample size."

We thank the reviewer for the positive comments. We fully agree that transparency regarding negative results is important. As discussed above, we have included the cox proportional hazards model including MGMT promoter methylation status for a subset of patients in the manuscript (with the caveat of relatively low statistical power for detection of and association between the risk score and survival).

(2) While I agree and do not necessarily have concerns about the model itself, I suggest that the authors discuss the variability in the model zoo and the potential usefulness for improving the performance, although the generalizability of recently proposed foundation models may not be encouraging (due to the limited availability of brain tissue samples compared to organs like the gastrointestinal tract, lungs, or breasts).

We agree that the choice of the model is an important issue, given the broad spectrum of available architectures. We aimed to highlight this in the discussion in our revised manuscript:
“Recent research efforts have given rise to many different deep learning models that may serve as an efficient backbone for computational pathology tasks (Deininger et al., 2022; Kang et al., 2022; Wang et al., 2022). The variability of pre-trained models can help to find solutions tailored to a wide range of tasks and requirements, and selecting and adapting the most appropriate model typically results in improved performance. While foundation models for computational pathology represent a significant advancement, the full potential of their generalizability across different organs and disease types requires thorough evaluation (Chen et al., 2024; Lu et al., 2024; Xu et al., 2024).”

(3) In addition, the authors may consider slightly toning down claims about the model in the presentation (as R1 also pointed out), such as "a novel CNN architecture". It would be beneficial to pivot the discussion towards the successful employment of a wellknown backbone model in this unique clinical case with customized modifications. This approach will help to avoid prolonged debates about why Xception might be more suitable than other models.
We concur that the approach suggested by this reviewer might benefit the discussion of our methods and results. As suggested, we have rephrased claims about the novelty of the model in the revised manuscript.

Thank you again for your work on this submission!

Best wishes,

Adelheid Woehrer and Thomas Roetzer-Pejrimovsky
Division of Neuropathology and Neurochemistry
Department of Neurology
Medical University of Vienna

References

Chen, R.J. et al. (2024) ‘Towards a general-purpose foundation model for computational pathology’, Nature medicine, 30(3), pp. 850–862.

Deininger, L. et al. (2022) ‘A comparative study between vision transformers and CNNs in digital pathology’, arXiv [eess.IV]. Available at: http://arxiv.org/abs/2206.00389.

Hsieh, F.Y. and Lavori, P.W. (2000) ‘Sample-size calculations for the Cox proportional hazards regression model with nonbinary covariates’, Controlled clinical trials, 21(6), pp. 552–560.

Kang, M. et al. (2022) ‘Benchmarking Self-Supervised Learning on Diverse Pathology Datasets’, arXiv [cs.CV]. Available at: http://arxiv.org/abs/2212.04690.

Lu, M.Y. et al. (2024) ‘A visual-language foundation model for computational pathology’, Nature medicine, 30(3), pp. 863–874.

Orzan, F. et al. (2020) ‘A simplified integrated molecular and immunohistochemistry-based algorithm allows high accuracy prediction of glioblastoma transcriptional subtypes’, Laboratory investigation; a journal of technical methods and pathology, 100(10), pp. 1330–1344.

Wang, X. et al. (2022) ‘Transformer-based unsupervised contrastive learning for histopathological image classification’, Medical image analysis, 81, p. 102559.

Xu, H. et al. (2024) ‘A whole-slide foundation model for digital pathology from real-world data’, Nature [Preprint]. Available at: https://doi.org/10.1038/s41586-024-07441-w.

Zheng, Y. et al. (2023) ‘Spatial cellular architecture predicts prognosis in glioblastoma’, Nature communications, 14(1), p. 4122.
