# Supplementary material for: Deep learning links localized digital pathology phenotypes with transcriptional subtype and patient outcome in glioblastoma
Source: Gigascience. 2024 Aug 26;13:giae057. doi: 10.1093/gigascience/giae057 (PMC11345537; doi:10.1093/gigascience/giae057)
Supplement: giae057_Reviewer_1_Report_Original_Submission — Hang Chang -- 11/18/2023 Reviewed [file giae057_reviewer_1_report_original_submission.pdf]

Reviewer Report

Title: Deep learning links localized digital pathology phenotypes with transcriptional subtype and patient outcome in glioblastoma

Version: Original Submission **Date:** 11/18/2023

Reviewer name: Hang Chang

Reviewer Comments to Author:

The authors in this manuscript deployed a deep learning model (i.e., Xception convolutional neural network) for the prediction of transcriptional subtype of prognosis from digital pathology slides in Glioblastoma. Deep learning has demonstrated power in digital pathology, and the development of deep learning models for the management of GBM patients has translational potentials. The major concerns are listed below,

1. The authors may need to review the literatures on the current state-of-the-arts in histology-based prognostic biomarker development in GBM. As mentioned in the Background section: "So far, no histology-based prognostic biomarker is available", this statement is incorrect. Many previous studies, reporting promising histology-related imaging prognostic biomarkers in GBM and different types of gliomas, can be found in PUBMED or using google search. Given this inaccurate statement, the reviewer is not fully convinced about the motivation and design of proposed study.
2. The choice of Xception model was unjustified and the RS-CNN model development was insufficiently described. Xception is a great deep learning model developed a while back, the choice of Xception model for the proposed task needs to be justified. In addition, it is not clear how the RS-CNN model was trained towards the survival outcome (i.e., time and event data). Did the author add another cox-regression layer to the network?
3. The multicox regression as shown in Table 3 is inappropriate. Important prognostic factors, including MGMT status, IDH mutation, G-CIMP, ATRX mutation, 1p/19q status, etc., were not included, without which, the independent value of RS CNN remains unjustified.
4. The external validation on TCGA-GBM cohort did not support the major conclusion. Specifically, the risk factor did not provide independent and significant prognostic value, and the predictive power towards transcriptional subtypes (although better than random guess) is far from the state-of-the-arts that have already been reported in literature (see comment #1). Given the validation outcome, the proposed work seems provide very limited clinical value.

In summary, the authors in this study tried to address an important question in GBM study. However, the study design and experiment outcomes make clinical value of proposed work insufficiently justified.

Methods

Are the methods appropriate to the aims of the study, are they well described, and are necessary controls included? Choose an item.

Conclusions

Are the conclusions adequately supported by the data shown? Choose an item.

Reporting Standards

Does the manuscript adhere to the journal's guidelines on minimum standards of reporting? Choose an item.

Choose an item.

Statistics

Are you able to assess all statistics in the manuscript, including the appropriateness of statistical tests used? Choose an item.

Quality of Written English

Please indicate the quality of language in the manuscript: Choose an item.

Declaration of Competing Interests

Please complete a declaration of competing interests, considering the following questions:

- Have you in the past five years received reimbursements, fees, funding, or salary from an organisation that may in any way gain or lose financially from the publication of this manuscript, either now or in the future?
- Do you hold any stocks or shares in an organisation that may in any way gain or lose financially from the publication of this manuscript, either now or in the future?
- Do you hold or are you currently applying for any patents relating to the content of the manuscript?
- Have you received reimbursements, fees, funding, or salary from an organization that holds or has applied for patents relating to the content of the manuscript?
- Do you have any other financial competing interests?
- Do you have any non-financial competing interests in relation to this paper?

If you can answer no to all of the above, write 'I declare that I have no competing interests' below. If your reply is yes to any, please give details below.

I declare that I have no competing interests

I agree to the open peer review policy of the journal. I understand that my name will be included on my report to the authors and, if the manuscript is accepted for publication, my named report including any attachments I upload will be posted on the website along with the authors' responses. I agree for my

report to be made available under an Open Access Creative Commons CC-BY license (http://creativecommons.org/licenses/by/4.0/). I understand that any comments which I do not wish to be included in my named report can be included as confidential comments to the editors, which will not be published.

Choose an item.

To further support our reviewers, we have joined with Publons, where you can gain additional credit to further highlight your hard work (see: https://publons.com/journal/530/gigascience). On publication of this paper, your review will be automatically added to Publons, you can then choose whether or not to claim your Publons credit. I understand this statement.

Yes Choose an item.
