# Supplementary material for: Deep learning links localized digital pathology phenotypes with transcriptional subtype and patient outcome in glioblastoma
Source: Gigascience. 2024 Aug 26;13:giae057. doi: 10.1093/gigascience/giae057 (PMC11345537; doi:10.1093/gigascience/giae057)
Supplement: giae057_Reviewer_2_Report_Original_Submission — Chris Armit -- 11/20/2023 Reviewed [file giae057_reviewer_2_report_original_submission.pdf]

Reviewer Report

Title: Deep learning links localized digital pathology phenotypes with transcriptional subtype and patient outcome in glioblastoma

Version: Original Submission **Date:** 11/20/2023

Reviewer name: Chris Armit

Reviewer Comments to Author:

This interesting Research Article utilises Deep Learning in the context of glioblastoma. The core concept here is the utility of an end-to-end Convolutional Neural Network (CNN) that generates a histology-based risk score to estimate patient prognosis (RS-CNN), and maps the spatial distribution of transcriptional subtypes (TS-CNN). I have been asked to provide a Data Review of the supporting data for the manuscript and I report below on this:
The complete slide scan library, including H&E stained slides and corresponding tissue segmentations as well as immunohistochemically stained slides, are available from the GBMatch supplementary website: https://www.medical-epigenomics.org/papers/GBMatch/#histo
All pre-selected image tiles used for training and segmentations for the immunohistochemically stained slides are available via the accompanying Zenodo repository: https://doi.org/10.5281/zenodo.8358673 [Creative Commons Zero v1.0 Universal Public Domain Dedication]
This Zenodo repository very helpfully includes all H&E image patches used for training.
The external TCGA validation dataset is available via cBioPortal and the GDC Data Portal, as detailed below:
-cBioPortal [https://www.cbioportal.org]
-GDC Data Portal [https://portal.gdc.cancer.gov];
The GBMatch_CNN software tool is publicly available on GitHub:
https://github.com/tovaroe/GBMatch_CNN [GPL-3.0 license]
In support of the Deep Learning study, the authors additionally submitted tabular data files to the GigaScience Database that report on the following:
-Basic clinical annotation
-Prediction results
-Nucleus phenotype for each single image tile
-Results of immunohistochemically stained slide for each present region for each patient
-First two UMAP (Uniform Manifold Approximation and Projection) dimensions for each patient
For Machine Learning studies, GigaScience requests that authors provide supporting annotations following the DOME-ML (Data, Optimisation, Model and Evaluation in Machine Learning) recommendations, and I was very impressed by the DOME annotation provided by the author. This includes detail of the following:
-Data Splits
-Pixel Size of Image Tiles
-Model (i.e. black box for single image tiles, but semi-interpretable for whole slide scan images)

-Performance Measures (e.g. AUC for prediction of transcriptional subtype)
The supporting DOME annotation can be found at the following link:
https://dome.ds-wizard.org/wizard/projects/a1a578d8-57cc-4706-ba8a-ae09c2bf423e

Methods

Are the methods appropriate to the aims of the study, are they well described, and are necessary controls included? Choose an item.

Conclusions

Are the conclusions adequately supported by the data shown? Choose an item.

Reporting Standards

Does the manuscript adhere to the journal's guidelines on minimum standards of reporting? Choose an item.

Choose an item.

Statistics

Are you able to assess all statistics in the manuscript, including the appropriateness of statistical tests used? Choose an item.

Quality of Written English

Please indicate the quality of language in the manuscript: Choose an item.

Declaration of Competing Interests

Please complete a declaration of competing interests, considering the following questions:

- Have you in the past five years received reimbursements, fees, funding, or salary from an organisation that may in any way gain or lose financially from the publication of this manuscript, either now or in the future?
- Do you hold any stocks or shares in an organisation that may in any way gain or lose financially from the publication of this manuscript, either now or in the future?
- Do you hold or are you currently applying for any patents relating to the content of the manuscript?
- Have you received reimbursements, fees, funding, or salary from an organization that holds or has applied for patents relating to the content of the manuscript?
- Do you have any other financial competing interests?
- Do you have any non-financial competing interests in relation to this paper?

If you can answer no to all of the above, write 'I declare that I have no competing interests' below. If your reply is yes to any, please give details below.

I work for GigaScience where I perform checks on the quality of image data and the image analysis approach. My funding is not dependent on the outcome of this review, and I declare that I have no competing interests.

I agree to the open peer review policy of the journal. I understand that my name will be included on my report to the authors and, if the manuscript is accepted for publication, my named report including any attachments I upload will be posted on the website along with the authors' responses. I agree for my report to be made available under an Open Access Creative Commons CC-BY license (http://creativecommons.org/licenses/by/4.0/). I understand that any comments which I do not wish to be included in my named report can be included as confidential comments to the editors, which will not be published.

Choose an item.

To further support our reviewers, we have joined with Publons, where you can gain additional credit to further highlight your hard work (see: https://publons.com/journal/530/gigascience). On publication of this paper, your review will be automatically added to Publons, you can then choose whether or not to claim your Publons credit. I understand this statement.

Yes Choose an item.
