# Supplementary material for: Deep learning links localized digital pathology phenotypes with transcriptional subtype and patient outcome in glioblastoma
Source: Gigascience. 2024 Aug 26;13:giae057. doi: 10.1093/gigascience/giae057 (PMC11345537; doi:10.1093/gigascience/giae057)
Supplement: giae057_Reviewer_3_Report_Original_Submission — Junhan Zhao -- 2/13/2024 Reviewed [file giae057_reviewer_3_report_original_submission.pdf]

Reviewer Report

Title: Deep learning links localized digital pathology phenotypes with transcriptional subtype and patient outcome in glioblastoma

Version: Original Submission **Date:** 2/13/2024

Reviewer name: Junhan Zhao

Reviewer Comments to Author:

In this study, the authors proposed RS/TS-CNNs and validated them using reasonably large "discovery cohort" and "validation cohort" collected from publicly available datasets for glioblastoma patients' prognostic and transcriptional subtype predictions. Both investigations are very clinically relevant. The study is well-executed, and the interpretations are supported by thoughtful post-hoc data analytics. Glioblastoma, one of the most prevalent and lethal CNS tumors, exhibits high inter- and intra-tumoral heterogeneity. One major contribution of this work is the use of widely available histology images to infer transcriptomic markers for patients, since transcriptomic profiling remains expensive and is not widely adopted in routine examinations in many geographical regions. The authors showed the potential of deep learning to identify and map transcriptional subtypesâ€"classical, mesenchymal, and proneuralâ€"to cellular morphological architecture, which could be useful for prognostic prediction and studies towards tumor microenvironment (Zheng et al., Spatial cellular architecture predicts prognosis in glioblastoma). While similar downstream tasks using histology images alone or in conjunction with biomarkers from gene panels have been explored (Chen et al., Pan-cancer integrative histology-genomic analysis via multimodal deep learning and subsequent works by other research groups in CVPR etc.), RS-CNN, built upon the Xception backbone pre-trained on ImageNet, captures clinically meaningful prognostic features, especially when generalized across different data sites.

To further provide insights to a broad audience, there are two major suggestions that the authors might consider including. First, the field of computational pathology seems to have shifted towards leveraging the benefits of pre-trained, self-supervised models as feature extractors. Considering the authors followed a similar two-step workflow (freezing the pre-trained network as a feature extractor, then training a head classifier), they could explore replacing the ImageNet pre-trained model with two published works, as literature has reported their advancements over ImageNet pre-trained extractors. This may further improve the performance of TS-CNN (Wang et al., Transformer-based Unsupervised Contrastive Learning for Histopathological Image Classification; Kang et al., Benchmarking Self-Supervised Learning on Diverse Pathology Datasets). A brief justification would also be valuable if the performance shows no significant difference. Second, while the current exploration and discussion are engaging, discussing some broader impacts would be worthwhile, such as the correlation between the outputs from TS and RS on the same slide, potential integration or comparison with other omics like spatial transcriptomes or genomes, and generalizability to other slide types (e.g., frozen sections and smears). Additionally, sharing the intermediate annotations prepared by the credible clinician team for training TS-CNN with the community would be beneficial, especially since these data are already public. This includes the manual segmentation described in the section "Handling of digital slides," as such

efforts can further aid in building cellular quantification tools for evaluating deep learning models for CNS. The authors could consider presenting these annotations as a contribution, referencing Amgad and Cooper's recent work in Nature Medicine on invasive breast cancer. Besides the works listed above, here are some very relevant works that the authors could also include:
Zeng, Y. et al. Spatial transcriptomics prediction from histology jointly through Transformer and graph neural networks. Brief. Bioinform. 23, bbac297 (2022).
Davis S, Scott C, Oetjen J, Charles PD, Kessler BM, Ansorge O, Fischer R. Deep topographic proteomics of a human brain tumour. Nat Commun. 2023
Shumin Dong, Catherine L. Nutt, Rebecca A. Betensky, Anat O. Stemmer-Rachamimov, Nicholas C. Denko, Keith L. Ligon, David H. Rowitch, David N. Louis, Histology-Based Expression Profiling Yields Novel Prognostic Markers in Human Glioblastoma, Journal of Neuropathology & Experimental Neurology
Nasrallah MP, Zhao J, Tsai CC, Meredith D, Marostica E, Ligon KL, Golden JA, Yu KH. Machine learning for cryosection pathology predicts the 2021 WHO classification of glioma. Med. 2023
Ozyoruk, K.B., Can, S., Darbaz, B. et al. A deep-learning model for transforming the style of tissue images from cryosectioned to formalin-fixed and paraffin-embedded. Nat. Biomed. Eng (2022)

Methods

Are the methods appropriate to the aims of the study, are they well described, and are necessary controls included? Choose an item.

Conclusions

Are the conclusions adequately supported by the data shown? Choose an item.

Reporting Standards

Does the manuscript adhere to the journal's guidelines on minimum standards of reporting? Choose an item.

Choose an item.

Statistics

Are you able to assess all statistics in the manuscript, including the appropriateness of statistical tests used? Choose an item.

Quality of Written English

Please indicate the quality of language in the manuscript: Choose an item.

Declaration of Competing Interests

Please complete a declaration of competing interests, considering the following questions:

- Have you in the past five years received reimbursements, fees, funding, or salary from an organisation that may in any way gain or lose financially from the publication of this manuscript, either now or in the future?
- Do you hold any stocks or shares in an organisation that may in any way gain or lose financially from the publication of this manuscript, either now or in the future?
- Do you hold or are you currently applying for any patents relating to the content of the manuscript?
- Have you received reimbursements, fees, funding, or salary from an organization that holds or has applied for patents relating to the content of the manuscript?
- Do you have any other financial competing interests?
- Do you have any non-financial competing interests in relation to this paper?

If you can answer no to all of the above, write 'I declare that I have no competing interests' below. If your reply is yes to any, please give details below.

I declare that I have no competing interests.

I agree to the open peer review policy of the journal. I understand that my name will be included on my report to the authors and, if the manuscript is accepted for publication, my named report including any attachments I upload will be posted on the website along with the authors' responses. I agree for my report to be made available under an Open Access Creative Commons CC-BY license (http://creativecommons.org/licenses/by/4.0/). I understand that any comments which I do not wish to be included in my named report can be included as confidential comments to the editors, which will not be published.

Choose an item.

To further support our reviewers, we have joined with Publons, where you can gain additional credit to further highlight your hard work (see: https://publons.com/journal/530/gigascience). On publication of this paper, your review will be automatically added to Publons, you can then choose whether or not to claim your Publons credit. I understand this statement.

Yes Choose an item.
