# Supplementary material for: Deep learning links localized digital pathology phenotypes with transcriptional subtype and patient outcome in glioblastoma
Source: Gigascience. 2024 Aug 26;13:giae057. doi: 10.1093/gigascience/giae057 (PMC11345537; doi:10.1093/gigascience/giae057)
Supplement: giae057_Reviewer_3_Report_Revision_1 — Junhan Zhao -- 5/24/2024 Reviewed [file giae057_reviewer_3_report_revision_1.pdf]

Reviewer Report

Title: Deep learning links localized digital pathology phenotypes with transcriptional subtype and patient outcome in glioblastoma

Version: Revision 1 **Date:** 5/24/2024

Reviewer name: Junhan Zhao

Reviewer Comments to Author:

The authors have conducted additional experiments and provided justifications in their writing to address previous comments. This work contributes to the community in two significant ways: (1) defining and developing a prototype deep learning solution that uses histological images and transcriptional subtypes to predict patient outcomes; and (2) providing fine-grained annotations for enabling using histology features to interpret deep learning outputs for Glioblastoma. While I am generally positive, I recommend that the authors conduct another editorial round of revision. Notably, most of the negative experimental results are valuable but have not been presented or discussed in the manuscript. For example, when exploring using MGMT as a prognostic factor in a Cox model, the observations may inspire further investigation by others. The authors state: "Given these caveats, we have recalculated the Cox proportional hazards model, including patients with RRBS-derived MGMT promoter status. In this model, only radiochemotherapy and age remain statistically significant prognostic factors. The predicted risk score and MGMT promoter methylation status fail to reach statistical significance, most likely due to the very limited sample size."
While I agree and do not necessarily have concerns about the model itself, I suggest that the authors discuss the variability in the model zoo and the potential usefulness for improving the performance, although the generalizability of recently proposed foundation models may not be encouraging (due to the limited availability of brain tissue samples compared to organs like the gastrointestinal tract, lungs, or breasts). In addition, the authors may consider slightly toning down claims about the model in the presentation (as R1 also pointed out), such as "a novel CNN architecture". It would be beneficial to pivot the discussion towards the successful employment of a wellknown backbone model in this unique clinical case with customized modifications. This approach will help to avoid prolonged debates about why Xception might be more suitable than other models.

Methods

Are the methods appropriate to the aims of the study, are they well described, and are necessary controls included? Choose an item.

Conclusions

Are the conclusions adequately supported by the data shown? Choose an item.

Reporting Standards

Does the manuscript adhere to the journal’s guidelines on [minimum standards of reporting?] Choose an item.

Choose an item.

Statistics

Are you able to assess all statistics in the manuscript, including the appropriateness of statistical tests used? Choose an item.

Quality of Written English

Please indicate the quality of language in the manuscript: Choose an item.

Declaration of Competing Interests

Please complete a declaration of competing interests, considering the following questions:

- Have you in the past five years received reimbursements, fees, funding, or salary from an organisation that may in any way gain or lose financially from the publication of this manuscript, either now or in the future?
- Do you hold any stocks or shares in an organisation that may in any way gain or lose financially from the publication of this manuscript, either now or in the future?
- Do you hold or are you currently applying for any patents relating to the content of the manuscript?
- Have you received reimbursements, fees, funding, or salary from an organization that holds or has applied for patents relating to the content of the manuscript?
- Do you have any other financial competing interests?
- Do you have any non-financial competing interests in relation to this paper?

If you can answer no to all of the above, write 'I declare that I have no competing interests' below. If your reply is yes to any, please give details below.

J. Zhao declares consulting service for Alvus Health.

I agree to the open peer review policy of the journal. I understand that my name will be included on my report to the authors and, if the manuscript is accepted for publication, my named report including any attachments I upload will be posted on the website along with the authors' responses. I agree for my report to be made available under an Open Access Creative Commons CC-BY license (http://creativecommons.org/licenses/by/4.0/). I understand that any comments which I do not wish to be included in my named report can be included as confidential comments to the editors, which will not be published.

Choose an item.

To further support our reviewers, we have joined with Publons, where you can gain additional credit to further highlight your hard work (see: https://publons.com/journal/530/gigascience). On publication of this paper, your review will be automatically added to Publons, you can then choose whether or not to claim your Publons credit. I understand this statement.

Yes Choose an item.
